# Analysis of critical protein–protein interactions of SARS-CoV-2 capping and proofreading molecular machineries towards designing dual target inhibitory peptides

**DOI:** 10.1038/s41598-022-26778-8

**Published:** 2023-01-07

**Authors:** Fatemeh Arabi-Jeshvaghani, Fatemeh Javadi‐Zarnaghi, Mohamad Reza Ganjalikhany

**Affiliations:** Department of Cell and Molecular Biology & Microbiology, Faculty of Biological Science and Technology, University of Isfahan, Isfahan, Iran

**Keywords:** Structural biology, Molecular modelling, Computational biology and bioinformatics, Biophysics, Computational biophysics

## Abstract

In recent years, the emergence of severe acute respiratory syndrome coronavirus 2 (SARS-CoV-2), as the cause of the coronavirus disease (COVID-19) global pandemic, and its variants, especially those with higher transmissibility and substantial immune evasion, have highlighted the imperative for developing novel therapeutics as sustainable solutions other than vaccination to combat coronaviruses (CoVs). Beside receptor recognition and virus entry, members of the SARS-CoV-2 replication/transcription complex are promising targets for designing antivirals. Here, the interacting residues that mediate protein–protein interactions (PPIs) of nsp10 with nsp16 and nsp14 were comprehensively analyzed, and the key residues’ interaction maps, interaction energies, structural networks, and dynamics were investigated. Nsp10 stimulates both nsp14’s exoribonuclease (ExoN) and nsp16’s 2′O-methyltransferase (2′O-MTase). Nsp14 ExoN is an RNA proofreading enzyme that supports replication fidelity. Nsp16 2′O-MTase is responsible for the completion of RNA capping to ensure efficient replication and translation and escape from the host cell’s innate immune system. The results of the PPIs analysis proposed crucial information with implications for designing SARS-CoV-2 antiviral drugs. Based on the predicted shared protein–protein interfaces of the nsp16-nsp10 and nsp14-nsp10 interactions, a set of dual-target peptide inhibitors was designed. The designed peptides were evaluated by molecular docking, peptide–protein interaction analysis, and free energy calculations, and then further optimized by in silico saturation mutagenesis. Based on the predicted evolutionary conservation of the interacted target residues among CoVs, the designed peptides have the potential to be developed as dual target pan-coronavirus inhibitors.

## Introduction

The novel human coronavirus disease 2019 (COVID-19), as a result of the infection by the severe acute respiratory syndrome coronavirus 2 (SARS-CoV-2)^1^, has caused a large number of confirmed deaths worldwide and a global economic crisis in recent years. SARS-CoV-2 is an enveloped spherical betacoronavirus belonging to the RNA virus family *Coronaviridae*^2^. The SARS-CoV-2 genome shares 96.2%, 79%, and 50% sequence identity with bat coronavirus, severe acute respiratory syndrome coronavirus (SARS-CoV), and Middle East respiratory syndrome coronavirus (MERS-CoV), respectively. The emergence of SARS-CoV-2 and then its variants, particularly the variants of concern (VOCs), together with the earlier emergencies of SARS-CoV in 2002–2003 with 8096 cases and 774 deaths (~ 10% fatality rate) and MERS-CoV in 2012 with 1728 confirmed cases and 624 deaths (~ 36% fatality rate)^3^ proved that coronaviruses (CoVs) have long been a major threat to humans. The pathogenicity of other human CoVs that cause the common cold should also be considered, particularly in infants and children^4^. The accelerated host cell entry of SARS-CoV-2 comparable to other CoVs^5^, and the advent of the Omicron variant (B.1.1.529) with higher transmissibility (3.2 times greater than Delta) and substantial immune evasion^6^, as well as its recent emerging sublineages, like BA.4 and BA.5, the efficacy of existing vaccines has been diminished, thereby promoting re-infections and vaccine evasion^7^. Therefore, the initial COVID-19 vaccines and therapeutics could not be prolonged solutions. Thus, in addition to developing diagnostic and surveillance technologies for SARS-CoV-2^8–10^, it is imperative to develop novel therapeutics to combat CoVs as sustainable solutions.

The open reading frames (ORFs) 1a/b are the largest ORFs in the SARS-CoV-2 genome. These ORFs are located at the 5′ end of the genome and encode two very large replicase polyprotein precursors, pp1a and pp1ab, which are cleaved post-translationally by viral proteases into 16 non-structural proteins (nsps)^11^ (Fig. S1). Nsp12, nsp13, nsp16, nsp14, nsp10, nsp7, and nsp8 are essential members of the SARS-CoV-2 replication and transcription complex (RTC), which is responsible for viral survival, evolution, and propagation. RTC promotes RNA replication, transcription, proofreading, and capping through complex assembly of nsp-nsp and nsp-viral RNA interactions^11–14^.

**Figure 1:**
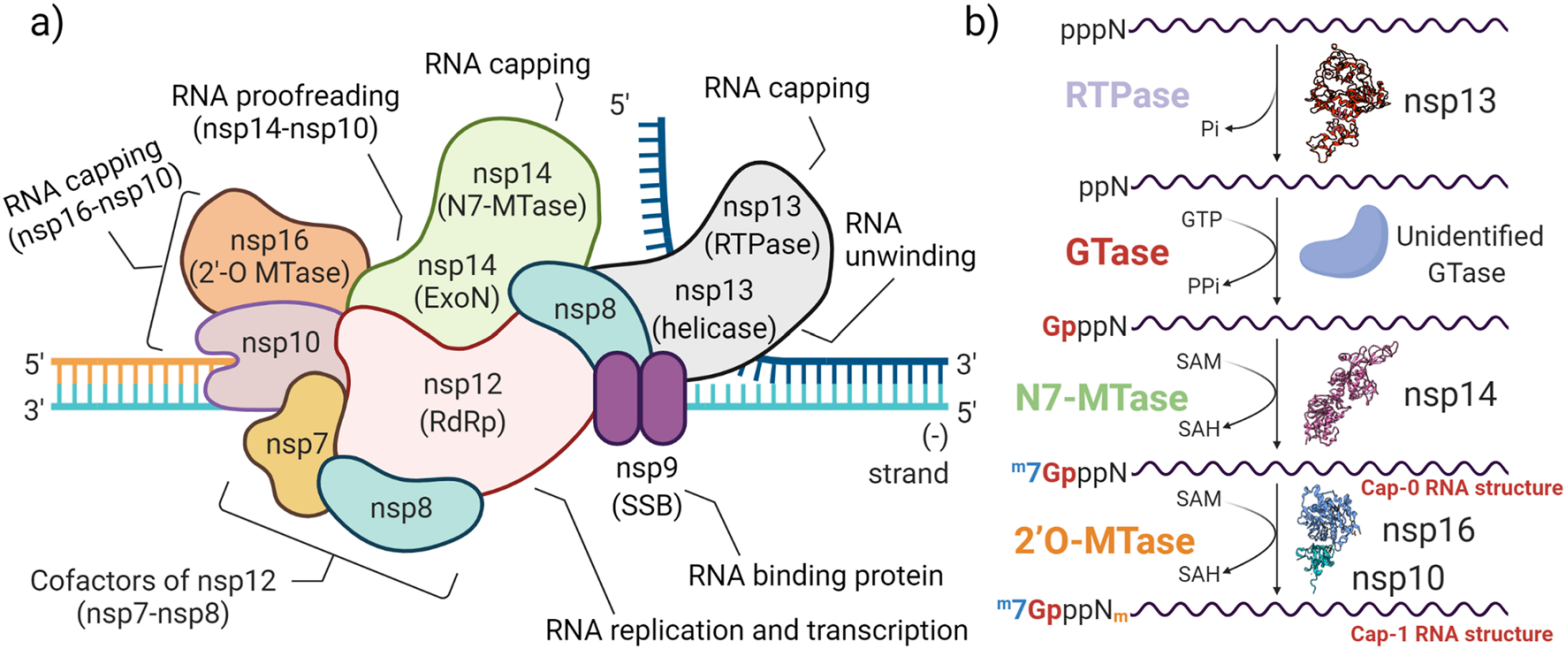
Schematic depiction of the SAR-CoV-2 replication and transcription complex (RTC) and its mRNA capping process. (**a**) SARS-CoV-2 RTC main members include RNA-dependent RNA polymerase (RdRp; nsp12), a multi-functional protein with helicase and RNA 5′ triphosphatase activities (Hel-RTPase; nsp13), methyltransferase enzymes (MTase; nsp16 and the C-terminal of nsp14), exoribonuclease (ExoN; the N-terminal of nsp14), and nsp10 as a critical activator for activating both nsp14 ExoN and nsp16 MTase. (**b**) The steps of SARS-CoV-2 mRNA capping process: First, the interphosphate bond at the 5′ end of the nascent triphosphorylated RNA (pppN) is hydrolyzed by nsp13 as RTPase, forming the diphosphate 5′-ppN transcript end. Second, guanosine triphosphate (GTP) is cleaved to guanosine monophosphate (GMP) by an unknown guanylyltransferase (GTase) and transferred to 5′-diphosphate mRNA to produce GpppN. Third, the cap-0 structure (7mGpppN) is formed by nsp14 N7-guanine-methyltransferase (N7-MTase) in the presence of S-adenosylmethionine (SAM). Last, the cap-1 structure is produced by the nsp16-nsp10 complex with 2′O-methyltransferase (2′O-MTase) activity.

An evolutionarily conserved co-transcriptional process in the nucleus of all eukaryotic cells assures modification of the 5′ end of nascent mRNAs with a cap. The mRNA cap performs as a preservative molecular group against exonuclease cleavage, stabilizes RNA molecules, ensures efficient nucleocytoplasmic transport, vigorous cap-dependent protein translation, and pre-mRNA processing, and also prevents non-self RNA distinction upon the innate immune response^15,16^. Capping of RNAs has been hijacked by viruses to overcome responses of the host immune system through diverse mechanisms. The *Coronaviridae* members have evolved their own specific capping process^17^. The capping mechanism of SARS-CoV-2 consists of four steps executed by a series of nsps, including nsp13 as RNA 5′ triphosphatase (RTPase), an unknown guanylyltransferase (GTase), the C-terminal of nsp14 for N7-guanine-methyltransferase (N7-MTase) activity, and nsp16 and its critical cofactor nsp10 for 2′O-methyltransferase (2′O-MTase) activity^12,18,19^ (Fig. 1b).

Studying the RTC members and, in particular, protein–protein interactions (PPIs) of nsps can be a productive means to design promising interfering inhibitors for CoVs, especially after viral entry into host cells. The vital role of PPIs in almost all biological processes makes them increasingly appealing for therapeutic approaches^20^. Recently, interfering peptides (IPs) have been more commonly acknowledged^21^, and some therapeutic peptides that target PPIs have been approved by the US Food and Drug Administration (FDA)^22^. Several studies have demonstrated the potency of derived peptides in inhibiting PPIs in CoVs^23–27^. In addition, clinical data and computational approaches have proposed SARS-CoV-2 nsps vaccines as immunodominant peptides with the capacity to confer global immunity towards COVID-19^28^.

We have recently investigated the protein–protein binding pattern of SARS-CoV-2 entry^29^. The overall objective of the current study was to shed light on the interactions of nsp10, as a common activator with both nsp16 capping MTase and nsp14 ExoN in the SARS-CoV-2 RTC, and then design peptide inhibitors to target these critical PPIs (Fig. S2). Initially, the critical interacting residues involved in these PPIs were investigated and then analyzed from the perspectives of free energy decomposition, interaction maps and energies, structural networks, and molecular dynamics (Fig. S3). These comprehensive analyses provide a whole picture of the critical residues that are of paramount importance for mediating capping and proofreading mechanisms in SARS-CoV-2. These key residues serve as druggable residues, with implications for SARS-CoV-2 drug design. Next, according to the obtained results, a set of dual target inhibitory peptides was designed based on the predicted shared protein–protein interfaces of nsp16-nsp10 and nsp14-nsp10 interactions. Thus, these peptides open up a novel route to simultaneously disturb SARS-CoV-2 RNA capping and proofreading machineries, with the potential to inhibit viral escape from host immune recognition, increase the rate of mutation, and consequently lead to lethal effects on SARS-CoV-2. The predicted evolutionary conservation across the broad spectrum of CoVs for the target residues that interacted with the designed peptides indicates the great potency of the designed peptides to be developed as new pan-coronavirus antivirals for current or future possible outbreaks related to CoVs.

## Methods

### Structures preparation

The Protein Data Bank (rcsb.org/)^30^ was used to obtain the nsp16-nsp10 complex structures of SARS-CoV-2 (PDB ID 6W75)^31^, SARS-CoV (PDB ID 2XYQ)^32^, MERS-CoV (PDB ID 5YNB), and HCoV-OC43 (PDB ID 7NH7)^33^. The SARS-CoV-2 nsp14-nsp10 complex structure was modeled by selecting the SARS-CoV nsp14-nsp10 complex (PDB ID 5C8S)^14^ as a template using SWISS-MODEL^34^.

### Protein–protein interface and hotspot residues prediction

The residues at the interfaces of the investigated protein–protein complexes were predicted using three in silico tools, including, PPCheck ^35^, PISA (Protein Interfaces, Surfaces and Assemblies) service at the European Bioinformatics Institute (http://www.ebi.ac.uk/pdbe/prot_int/pistart.html)^36^, and ISPRED4^37^. Next, the hotspot residues were predicted using KFC2 (Knowledge-based FADE and Contacts)^38^, DrugScore^PPI^^39^, and Robetta^40^. To improve the accuracy of the results, a combination of tools was used to identify protein–protein interfaces and hotspots. A residue was considered a key residue (interface and/or hotspot) if it was present at least in the results of two of the three tools. Moreover, for comparative analysis, the PPIs interface and hotspot residues of the nsp16-nsp10 complex of SARS-CoV, MERS-CoV, and HCoV-OC43 were also predicted based on the above approach. The key residues were mapped to the structures of proteins by UCSF Chimera^41^. Secondary structure plot for both complexes were predicted using PDBsum^42^. Next, computational alanine scanning (CAS) of the predicted key interacting residues of the SARS-CoV-2 nsp16-nsp10 and nsp14-nsp10 complexes was performed by the mCSM-PPI2 (http://biosig.unimelb.edu.au/mcsm_ppi2/), and the impact of each mutation was assessed by predicting the difference in protein–protein binding affinities between the wild-type and the mutant, which is defined as ΔΔG_Affinity_ (ΔΔG = ΔG_Mutant_ − ΔG_wild-type_)^43^. Also, the interactions between the wild-type and mutant residues with the most negative ΔΔG_Affinity_ and their nearby residues were analyzed. Then, per-residue free energy decomposition analysis by Molecular Mechanics-Generalized Born Surface Area (MM-GBSA) calculations was performed by the HawkDock server (cadd.zju.edu.cn/hawkdock)^44^ for the SARS-CoV-2 nsp16-nsp10 and nsp14-nsp10 complexes.

### Investigation of residue interactions

The interactions of the residues were analyzed and displayed in 2D maps by employing DIMPLOT from Ligplot^+^^45^. Also, the Protein Interactions Calculator (PIC) server (pic.mbu.iisc.ernet.in)^46^ was used to analyze different types of interactions. In addition, COCOMAPS (molnac.unisa.it/BioTools/cocomaps)^47^ was utilized to analyze the investigated PPIs interfaces at an 8 Å cut-off distance by providing the distance and property contact maps. In addition, the accessible surface areas of the complexes were predicted by COCOMAPS. Moreover, the interaction energies (IEs) between residues were predicted by the Amino Acid Interactions (INTAA) web server (bioinfo.uochb.cas.cz/INTAA/)^48^ with the help of the AMBER parm99 force field, water-like (OBC-II) environment, and the add hydrogen mode. Heat maps were generated using THE Heatmapper^49^. The entire procedure was performed for both SARS-CoV-2 nsp16-nsp10 and nsp14-nsp10 complexes.

### Structural networks analysis

The Network Analysis of Protein Structures (NAPS) server (bioinf.iiit.ac.in/NAPS/index.php)^50^ was used for the structural network analysis of the SARS-CoV-2 nsp16-nsp10 and nsp14-nsp10 complexes. The node centrality parameters, including degree centrality (DC), betweenness centrality (BC), and closeness centrality (CC) of residues in the network, were predicted by selecting the C-alpha unweighted network type with an upper threshold of 7 Å in the protein complex mode of NAPS.

### Molecular dynamics simulations

Molecular dynamics (MD) simulations of nsp14-nsp10, nsp16-nsp10 complexes, as well as nsp14, nsp16, and nsp10 proteins were performed by the AMBER14 package^51^ using the ff14SB force field^52^. The systems were solvated by the explicit water TIP3P model in a truncated octahedral box at a 12 Å hydration layer using *xleap.* Five chloride ions were added to neutralize the systems. All covalent bonds for the hydrogen atoms were added to the systems using the SHAKE algorithm^53^. The electrostatic interactions were calculated using the PME (Particle Mesh Ewald) algorithm^54^ with a threshold of 10 Å for the Lennard–Jones potential interactions under periodic boundary conditions. The minimization step was performed to remove high energy contacts using 2000 steps of the steepest-descent and 3000 steps of the conjugate gradient methods, respectively. Each system was then completely heated from 0 to 300 K over 400 ps in a constant volume. The equilibrium of the systems was carried out under the constant pressure and temperature conditions for 1 ns. After equilibration, the simulations were performed using *pmemd* over a period of 100 ns. The Langevin algorithm^55^ was used to control the temperature within the simulations. Finally, the trajectories were analyzed using *cpptraj*^56^. XMGRACE^57^ was used to generate root mean square deviation (RMSD) and root mean square fluctuation (RMSF) plots.

### Computational design of peptide inhibitors

In this study, the inhibitory peptides were designed based on two methodologies. In the first approach, the inhibitory peptides were designed manually based on the predicted overlapping interacting residues of nsp10, which interact with both the nsp16 and nsp14 in SARS-CoV-2. In the second approach, PPIs at the interfaces of the SARS-CoV-2 nsp16-nsp10 and nsp14-nsp10 complexes were analyzed individually using the Peptiderive^58^ server to design peptide inhibitors to target nsp16/nsp14. Peptiderive systematically separated peptides from nsp10 with a sliding window (with 5–15 lengths at each prediction stage), and then evaluated the contributions of these isolated peptides to the overall interaction.

### Molecular docking of the designed peptides

In the first step, molecular docking was performed using the HPEPDOCK server (http://huanglab.phys.hust.edu.cn/hpepdock/)^59^ for local docking of the designed peptides with their respective target(s). The binding site residues of the target were specified as a reference based on the target predicted key interacting residues. The best model selection criteria from the top 10 predicted models for each peptide-target complex were as follows: (i) the model that was bound to the target’s key residues with a similar pose relative to the reference interaction, and interacted with more key residues of the target; (ii) the model with relatively low RMSD among all the predicted models, which was examined by superimposing with the reference structure; and (iii) the model with the lowest docking energy score. Each model of the peptide–protein complex was analyzed using DIMPLOT^45^ and UCSF Chimera^41^. Based on the HPEPDOCK results, the best peptides were subjected to further docking analysis. Then, the 3D structures of the selected peptides were modeled using MODPEP (http://huanglab.phys.hust.edu.cn/modpep/)^60^. The best model was chosen, and this step was followed by peptide–protein docking using HADDOCK 2.4^61^. The predicted key interacting residues from the previous steps were specified as active residues, which were directly involved in the peptide–protein interaction, and all the surrounding surface residues within the 6.5 Å radius around the active residues were defined as passive residues. Of note, the ExoN domain of nsp14 was used for molecular docking. Here, the same best model selection criteria were used as in the previous step. The binding free energy (ΔG) and dissociation constant (K_d_) of the best complex of the designed peptide-target obtained by the second step of docking were predicted using Prodigy (wenmr.science.uu.nl/prodigy)^62^. Furthermore, MM-GBSA calculations using the ff02 force field, 2000 cycles of steepest descent, and 3000 cycles of conjugate gradient minimizations in the HawkDock (cadd.zju.edu.cn/hawkDock)^44^ were used to compute the binding free energies of peptide–protein complexes and break them down into per-residue contributions.

### Free energy calculations by the molecular mechanics Poisson–Boltzmann Surface Area (MM-PBSA) method

The MM-PBSA^63^ method was used to estimate the binding free energy (ΔG_binding_) of the best scoring designed peptides to their respective target(s). For 50 ns, MD simulations of the best scoring peptide–protein complexes were done with AMBER14^51^. Then, 200 snapshots of peptide–protein complexes were taken from the trajectory at various time steps to calculate the average values of ΔG_binding_ by *mmpbsa.py*^64^.

### Optimization of the best designed peptides

The best scoring designed peptides were optimized using in silico saturation mutagenesis analysis by mutating each residue of the designed peptides as lead sequences to the other 19 amino acids to generate a new peptide library. Then, the change in binding affinity of the new mutant peptide-target relative to the lead peptide-target complex (ΔΔG_Affinity_), and the impact of each mutation on peptide-target binding affinity were predicted using mCSM-PPI2^43^. To further assess the designed peptides, their general properties, including molecular weight, isoelectric point, and net charge at pH 7, were calculated using the Pep-CaLc^65^. Also, the pharmacokinetic properties of the designed peptides were predicted by pkCSM (http://biosig.unimelb.edu.au/pkcsm/)^66^. The half maximal inhibitory concentration (IC_50_) of the designed peptides was predicted by the AVP-IC_50_Pred (crdd.osdd.net/servers/ic50avp/) using the Random Forest (RFs) machine learning technique^67^. The allergenicity and toxicity of the peptides were predicted by AllergenFP v.1.0^68^ and ToxinPred^69^, respectively. The target protein-peptide complexes were simulated using CABS-flex 2.0 (biocomp.chem.uw.edu.pl/CABSflex2/index)^70^ with default parameters to analyze the fluctuations of residues and generate the RMSF plots.

### Conservation analysis among CoVs

Multiple sequence alignments of nsp10, nsp16, and nsp14 sequences from seven human CoVs, including SARS-CoV-2, SARS-CoV, MERS-CoV, HCoV-OC43, HCoV-HKU1, HCoV-NL63, and HCoV-229E, were conducted using MultAlin^71^. ESPrip was used to render the sequences^72^. To explore the evolutionary conserved residues of the nsp10 and nsp16 proteins in all the reported structures of the nsp16-nsp10 complex, including SARS-CoV-2, SARS-CoV, MERS-CoV, and HCoV-OC43, the ConSurf web server (consurf.tau.ac.il/)^73^ was employed based on the Bayesian method. Furthermore, the amino acid conservation of the SARS-CoV-2 nsp14 protein model was analyzed.

## Results

### Predicted protein–protein interfaces and hotspot residues of nsp16-nsp10 and nsp14-nsp10 interactions

**Figure 2:**
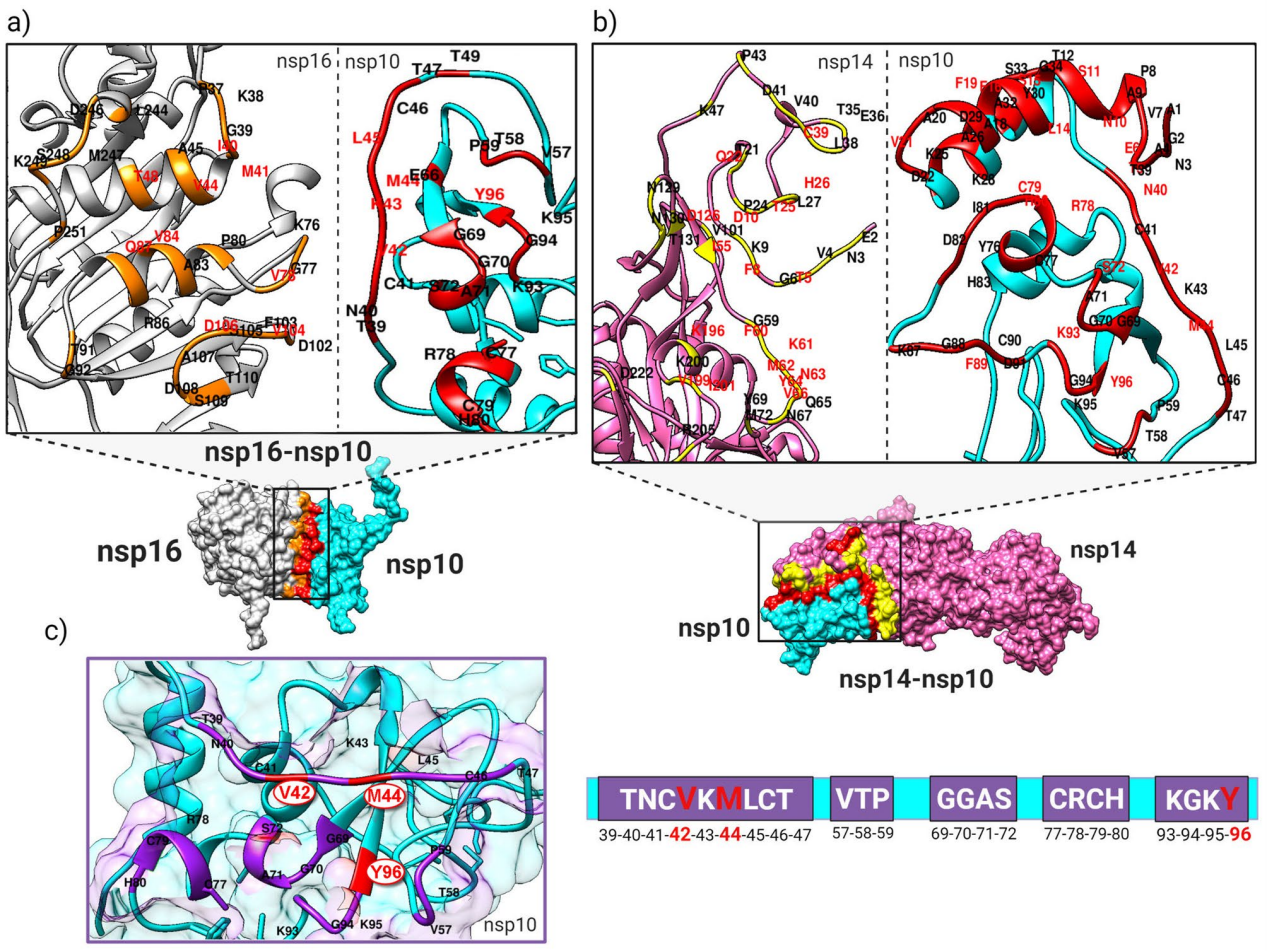
Analysis of the SARS-CoV-2 nsp16-nsp10 and nsp14-nsp10 protein–protein interfaces and hotspot residues. (**a**) The interfaces and hotspots were mapped to the nsp16-nsp10 structure at the protein–protein interfaces of nsp16 (gray) and nsp10 (cyan), which are colored orange and red, respectively. (**b**) The interfaces and hotspots at the protein–protein interfaces of nsp14 (magenta) and nsp10 (cyan) are colored yellow and red, respectively. Hotspot residues are shown in red labels. (**c**) The overlapping key interface (purple) and hotspot (red) residues of nsp10 (cyan), which interacted with both the nsp16 and nsp14 proteins.

In this study, first, the key residues at the interface of protein–protein complexes mediating PPIs were predicted. A depiction of the workflow of this study is presented at Fig. S3. The final predicted protein–protein interface and hotspot residues of the SARS-CoV-2 nsp16-nsp10 and nsp14-nsp10 complexes are listed in Table 1. The key interacting residues predicted using each tool are listed in Tables S1. For the nsp16-nsp10 complex, 33 residues were predicted as nsp16 interface residues, and 26 residues were predicted as nsp10 interfacial residues. V42, K43, M44, L45, and Y96 of nsp10, and I40, M41, V44, T48, V78, V84, Q87, V104, and D106 of nsp16 were predicted as hotspot residues for the nsp16-nsp10 complex. For the nsp14-nsp10 complex, 106 residues were identified at the protein–protein interface, 45 and 61 residues at the interfaces of nsp14 and nsp10, respectively. T5, E6, N10, S11, L14, S15, F16, F19, V21, N40, V42, M44, S72, R78, C79, H80, F89, K93, and Y96, were predicted as nsp10 hotspots in the nsp14-nsp10 complex. The key interacting residues for both complexes were mapped to their structures (Fig. 2a,b). Also, the secondary structure plots for the SARS-CoV-2 nsp16-nsp10 and nsp14-nsp10 complexes are shown in Figs S4 and S5, respectively.

**Table 1:** The predicted protein–protein interface and hotspot residues of the SARS-CoV-2 nsp16-nsp10 and nsp14-nsp10 complexes.

Protein	Predicted interface residues	Predicted hotspot residues
nsp10 (nsp16-nsp10 interaction)	T39, N40, C41, V42, K43, M44, L45, C46, T47, T49, V57, T58, P59, E66, G69, G70, A71, S72, C77, R78, C79, H80, K93, G94, K95, Y96	V42, K43, M44, L45, Y96
nsp16	P37, K38, G39, I40, M41, V44, A45, T48, K76, G77, V78, P80, A83, V84, R86, Q87, T91, G92, D102, F103, V104, S105, D106, A107, D108, S109, T110, L244, D246, M247, S248, K249, P251	I40, M41, V44, T48, V78, V84, Q87, V104, D106
nsp10 (nsp14-nsp10 interaction)	A1, G2, N3, A4, T5, E6, V7, P8, A9, N10, S11, T12, L14, S15, F16, A18, F19, A20, V21, D22, K25, A26, K28, D29, Y30, A32, S33, G34, T39, N40, C41, V42, K43, M44, L45, C46, T47, V57, T58, P59, G69, G70, A71, S72, Y76, C77, R78, C79, H80, I81, D82, H83, K87, G88, F89, C90, D91, K93, G94, K95, Y96	T5, E6, N10, S11, L14, S15, F16, F19, V21, N40, V42, M44, S72, R78, C79, H80, F89, K93, Y96
nsp14	E2, N3, V4, T5, G6, F8, K9, D10, T21, Q22, P24, T25, H26, L27, T35, E36, L38, C39, V40, D41, P43, K47, I55, G59, F60, K61, M62, N63, Y64, Q65, V66, N67, Y69, M72, V101, D126, N129, N130, T131, K196, V199, K200, I201, R205, D222	T5, F8, D10, Q22, T25, H26, C39, I55, F60, K61, M62, N63, Y64, V66, D126, K196, V199, I201

Notably, among the key interacting residues of nsp10, 24 interface residues and three hotspot residues were shared between the two complexes in SAR-CoV-2. These key residues of nsp10 interacted with both nsp16 (2′O-MTase capping) and nsp14 (ExoN proofreading). The overlapping common interface residues of nsp10 in these two complexes were T39, N40, C41, V42, K43, M44, L45, C46, T47, V57, T58, P59, G69, G70, A71, S72, C77, R78, C79, H80, K93, G94, K95, and Y96. Common hotspots were V42, M44, and Y96 (Fig. 2c). In addition, the analysis of PPIs interfacial and hotspot residues indicated the interactions between the residues in the N-terminal loop and H1 helix of the nsp10 and nsp14 ExoN domain. Moreover, to elucidate the similarities and differences in the interactions of nsp10 with nsp16 among CoVs, the protein–protein interfaces and hotspots of the nsp16-nsp10 complex in SARS-CoV, MERS-CoV, and HCoV-OC43 were also predicted by applying the same approach (Table S1). Residues, including V42, K43, M44, and L45, were common hotspots of nsp10 in all these CoVs. The differences lied in T47, R78, and Y96 of nsp10 in SARS-CoV; K58, H80, and F96 of nsp10 in MERS-CoV; and N40, C41, C46, D47, V57, K58, S72, C77, R78, H80, L89, C90, R93, K95, and F96 of nsp10 in HCoV-OC43. V84, Q87, V104, and D106 were the shared hotspots of nsp16 among these CoVs (Figs. S6–S8).

Next, the predicted key interacting residues were analyzed by CAS using mCSM-PPI2. In total, 153 mutations were analyzed to predict the impact of alanine substitution on the affinity of protein–protein binding (Table S2). As shown in Fig. S9, the mutations, respectively including Y96A, L45A, V42A, M44A, and G70A of nsp10; V84A, D106A, V44A, I40A, and R86A of nsp16 in the nsp16-nsp10 complex; F16A, Y96A, H80A, E6A, and F19A of nsp10, and F8A, P24A, F60A, Q22A, and D10A of nsp14 in the nsp14-nsp10 complex; showed the most negative ΔΔG_Affinity_, with the greatest decreasing impacts on the nsp16-nsp10 and the nsp14-nsp10 binding affinities. These results revealed the critical role of these residues in mediating PPIs. All of these residues, except G70 of nsp10, R86 of nsp16, and P24 of nsp14, were predicted as hotspots in the preceding step. However, mutations, including T49A, V57A, and C79A of nsp10, and T91A, G92A, and S248A of nsp16 in the nsp16-nsp10 complex, P8A, S33A, V57A, and C79A of nsp10, and T21A, C39A, and K47A of nsp14 in the nsp14-nsp10 complex, showed small positive ΔΔG_Affinity_ values (< 1.3 kcal/mol), indicating the least importance of such residues for complex formation. The interactions between the wild-type and mutant residues with the most negative ΔΔG_Affinity_ and their nearby residues are shown in Figs. S10–S13. Moreover, the results of per-residue free energy decomposition analysis using the MM-GBSA method for the nsp16-nsp10 and nsp14-nsp10 complexes are shown in Table S3. These results indicated that the key interacting residues significantly contributed to the PPIs binding free energy compared to other residues. The lowest estimated binding free energies of L45, V42 and M44 of nsp10, and Q87, I40, and V104 of nsp16 in the nsp16-nsp10 complex; and F19, V21, and H80 of nsp10, and N130, H26, and I201 of nsp14 in the nsp14-nsp10 complex indicated their greatest contributions in these two PPIs, respectively. The MM-GBSA results were consistent with the hotspot prediction and CAS results; however, the N130 of nsp14 was not predicted as a hotspot in the preceding step.

### Investigation of the residues interactions

According to analysis of the 2D maps of residue-residue interactions in the SARS-CoV-2 nsp16-nsp10 complex, the hydrophobic interactions were the most abundant interactions (Fig. S14a). N40, V42, K43, M44, L45, P59, A71, K93, and Y96 of nsp10 contributed to several hydrophobic interactions with the nsp16 residues. In addition, K43, L45, A71, K93, G94, and Y96 of nsp10 formed H-bonds with the residues of nsp16. A71 and G94 of nsp10 participated in H-bonds with D106 of nsp16. Moreover, K43, L45, K93, and Y96 of nsp10 formed H-bonds with K38, Q87, S105, and A83 of nsp16, respectively. Analysis of the nsp16-nsp10 interaction by PIC generated the same results as DIMPLOT (Table S4), indicating that the hydrophobic interactions were predominant. Moreover, PIC analysis showed that E66 and H80 of nsp10 formed ionic interactions with K38 and D102 of nsp16, respectively. Also, to perform a comparative analysis, the 2D map plots of residue interactions for the nsp16-nsp10 complex in other CoVs (SARS-CoV, MERS-CoV, and HCoV-OC43) were generated, and the results are represented in Figs. S15–S17.

According to the DIMPLOT results for the SARS-CoV-2 nsp14-nsp10 complex (Fig. S14b), the interactions of the residues were predominantly hydrophobic at the protein–protein interfaces, similar to what was observed for the nsp16-nsp10 complex. H-bond analysis revealed that K43 and L45 of nsp10 formed H-bonds with the C39 of nsp14. Other H-bonds were found between T5, E6, and S15 of nsp10 and S28, T5, and F60 of nsp14, respectively. K93 of nsp10 contributed to the interactions with D126 and T127 of nsp14 through two H-bonds. Also, H-bonds were formed between N40, G94, and Y96 of nsp10 and H29, K47, and D41 of nsp14, respectively. Similarly, the results of DIMPLOT and PIC were in the same line for the nsp14-nsp10 complex. Additionally, other different types of interactions, i.e., ionic, aromatic-sulfur, aromatic-aromatic, and cation-pi were predicted by PIC for the nsp14-nsp10 complex (Table S4). The differences in the interactions of SARS-CoV-2 nsp10 with nsp16 and nsp14 mainly lied in the interactions of residues at the N-terminal of nsp10 with nsp14, the interactions like, H-bonds between A1, A18, F19, V21, and D29 of nsp10 and K9, K196, I201, I201, and Y69 of nsp14, respectively. In addition, N3 of nsp10 formed two H-bonds with D10 of nsp14.

The intermolecular contacts with an 8 Å cut-off distance were analyzed using COCOMAPS to represent the nsp16/nsp14 regions that are in contact with the SARS-CoV-2 nsp10. In the distance range contact maps (Fig. S18a), the intermolecular contacts of both complexes are colored according to increasing distance. Residue pairs from the nsp10 and nsp16, including Y96-A83, K43-K38, K93-S105, L45-Q87, and G94-D106, exhibited a minimum distance of < 2.82 Å. In addition, the residue pairs including, F60-S15, K9-A1, and Y69-D29 of nsp14 and nsp10 were observed in the nsp14-nsp10 complex with a minimum distance of < 2.64 Å. The contact map of the nsp14-nsp10 complex may indicate the role of nsp10 interacting residues in activating the nsp14 ExoN at the N-terminal. No intermolecular contact was observed between nsp10 and the C-terminal of nsp14. The physicochemical nature of the interactions is indicated by the property contact maps (Fig. S18b). As elucidated in the property maps, the hydrophobic interactions contributed more than the hydrophilic interactions for both complexes. These results were in good agreement with the DIMPLOT and PIC results. Moreover, COCOMAPS showed that a large area of approximately 1852.3 Å^2^ was buried upon the formation of the SARS-CoV-2 nsp16-nsp10 complex, and its large interface area was approximately 926.75 Å^2^. Similarly, the buried area and interface area of the SARS-CoV-2 nsp14-nsp10 upon complex formation were both large, measuring 4358.47 and 2180.15 Å^2^, respectively.

Next, the residue-residue interaction energies (IEs) of the SARS-CoV-2 nsp16-nsp10 and nsp14-nsp10 complexes were analyzed using INTAA. According to the results, D106 of nsp10 and D125 of nsp16 showed the most negative net IEs (− 190.6 and − 518.33 kJ/mol, respectively). Among the predicted key interacting residues, E66, Y96, and S72 of nsp10 as well as D106, D108, and S105 of nsp16 showed the most negative net IEs. Figure S19 shows a heat map for residue-residue pairwise IEs between the predicted key interacting residues of nsp10 and nsp16. The most negative pairwise IEs with stabilizing roles in PPIs included the interactions between K93 and D106, K93 and S105, A71 and D106, G94 and D106 of nsp10 and nsp16, respectively. As aforementioned, the distances of the pair of residues, including K93 and S105, as well as G94 and D106, were predicted among the minimum distances in the previous step by COCOMAPS. IEs analysis of the nsp14-nsp10 complex revealed that E6 of nsp10 and E284 of nsp14 showed the most negative net IEs (− 504.75 and − 296.05 kJ/mol, respectively). E6, D29, K25, and D22 of nsp10 and D10, D126, E2, and F60 of nsp14 showed the most negative net IEs among the predicted key interacting residues in the nsp14-nsp10 complex. The interactions between D29 and Y69, S15 and F60, F19 and K200, E6 and V4 of nsp10 and nsp14, respectively, were the most stabilizing pairwise IEs (Fig. S20). All these residues with the most stabilizing roles in PPIs, were predicted as the key interacting residues in the foregoing steps.

### Structural network analysis of the SARS-CoV-2 nsp16-nsp10 and nsp14-nsp10 complexes

Protein contact network (PCN) analyses of the SARS-CoV-2 nsp16-nsp10 and nsp14-nsp10 complexes were performed to determine the topological significance of each residue as a node in the protein–protein network. The node centrality parameters of the residues, including the degree centrality (DC), betweenness centrality (BC), and closeness centrality (CC) of the residues in the network were predicted for both complexes (Table S5). In the nsp16-nsp10 complex, G69, G70, Y96, A71, and K95 of nsp10, and V44, A45, V84, and D106 of nsp16 showed the highest DC among the predicted key residues. The results revealed that G69, M44, G70, G94, and K95 of nsp10, and V44, D106, D108, T91, and I40 of nsp16 showed the highest BC among the critical PPI residues. High CC values of the key residues were predicted for M44, G69, V57, K43, and G94 of nsp10, and V44, D106, V84, and A107 of nsp16 (Fig. S21). PCN analysis of the predicted key interacting residues of the nsp14-nsp10 interaction showed that among the critical interacting residues, G70, Y96, A71, K95, and A20 of nsp10, and K9, D10, I55, and T131 of nsp14 showed high DC. Notably, G70, A71, K95, and Y96 were also predicted to have a high DC in the nsp16-nsp10 complex, indicating that these networks were common in both complexes. Moreover, F19, S15, A20, A18, and V21 of nsp10, and G59, F60, and Y69 of nsp14 showed the highest CC among the key residues. High BC values of the key residues were predicted for T5, A18, C79, A20, and F19 of nsp10, and G6, T25, G59, Y69, and F60 of nsp14 (Fig. S22). The 3D networks of the nsp16-nsp10 and nsp14-nsp10 complexes and their highlighted interfaces are shown in Fig. S23.

### MD simulations of the capping and proofreading components of SARS-CoV-2

**Figure 3:**
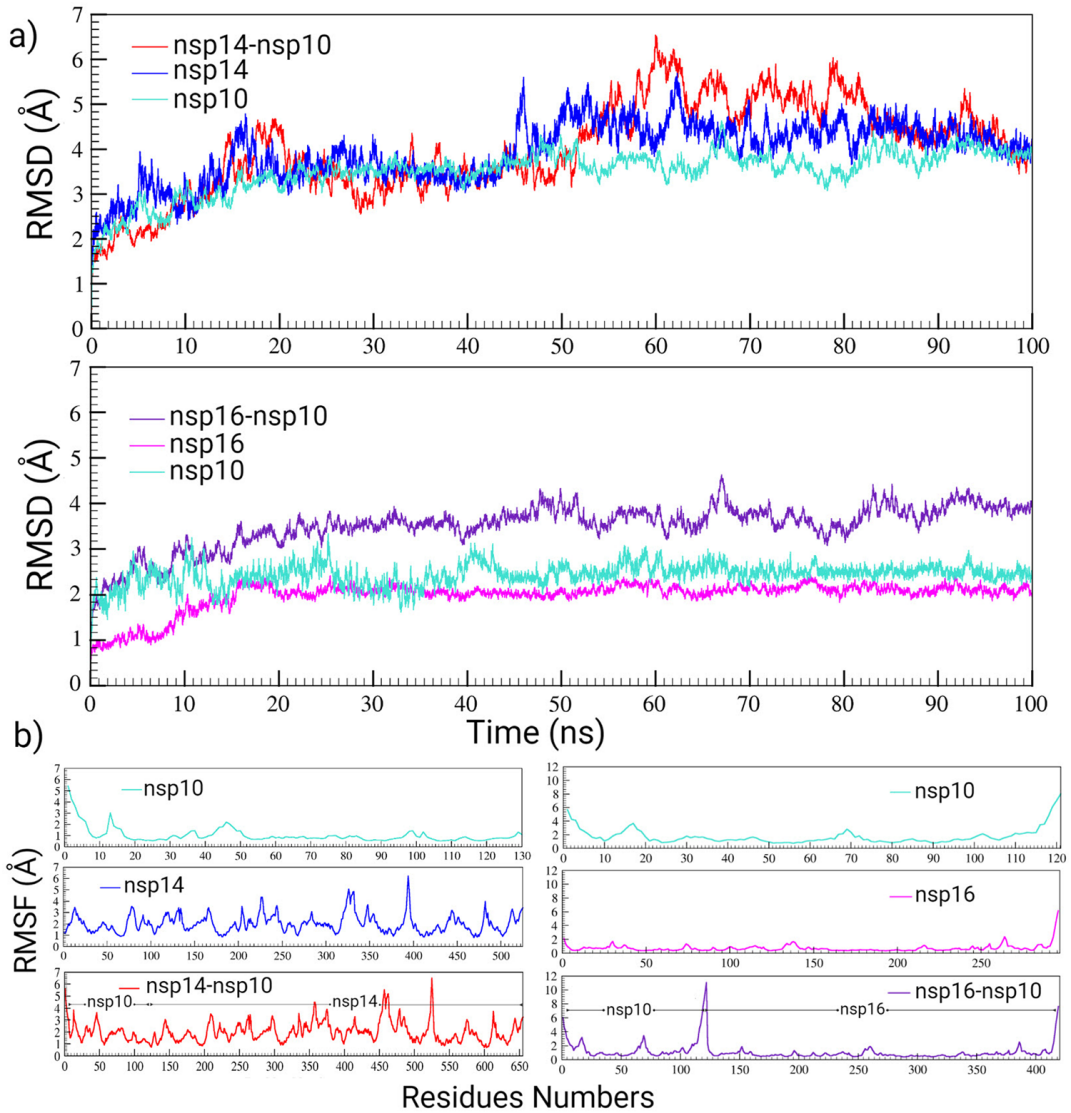
RMSD and RMSF plots for the SARS-CoV-2 nsp14-nsp10 and nsp16-nsp10 complexes during 100 ns of MD simulations. (**a**) RMSD plot for nsp14-nsp10, nsp14, nsp10, nsp16-nsp10, and nsp16, and (**b**) RMSF plots for nsp10, nsp14, nsp14-nsp10, nsp16, and nsp16-nsp10.

To investigate the stability, fluctuations of residues, and dynamic behavior of proteins, 100 ns MD simulations of the capping and proofreading components of SARS-CoV-2 were analyzed. The RMSDs of the backbones, as one of the criteria for structural stability during the simulations, were calculated in comparison with the reference structure. As implied in Fig. 3a, the RMSDs of the structures had an initial rise, increased during the first 45 ns, and then remained stable. These results indicated that the conformational changes were minor, and the nsp14 and nsp10 binding was stable. Nsp14 had greater RMSD values than nsp10, indicating that it was more flexible in the free form. To analyze the fluctuations of residues, RMSFs of the Cα atoms were calculated. Higher values of RMSFs in nsp10 were observed mainly in the N-terminal loop (A1-V7), the H1 helix (S11-F19), and in the coil and strand region (V42-T47) (Fig. 3b). Higher fluctuations of the H1 helix in nsp10 may be considered as an indication of its role in nsp14 ExoN activation. In the nsp14 structure, the regions mediating the interactions with nsp10 showed more fluctuations which were located at the protein N-terminal domain. MD simulations showed that two particular domains of nsp14 were connected via a hinge region. This hinge region may separate the ExoN and N7-MTas activity of nsp14. The RMSDs of the backbone were calculated with respect to the reference structure for the nsp16-nsp10 complex as well as free forms of the nsp16 and nsp10 proteins (Fig. 3a). The RMSD values were relatively stable after 30 ns. The average RMSD values for nsp16-nsp10, nsp16, and nsp10 were 3.6, 2.4, and 2.05 Å, respectively. Next, the RMSF values for the nsp16-nsp10 complex, nsp16, and nsp10 proteins were calculated (Fig. 3b).

### Computational design of peptide inhibitors to target the SARS-CoV-2 nsp16-nsp10 and nsp14-nsp10 interactions

The results of the PPIs analysis establish an appropriate context for drug design. The results of the aforementioned analyses prompted us to design peptide inhibitors by targeting the investigated interactions to disrupt SARS-CoV-2 capping and proofreading mechanisms. To this end, in an initial approach, given the predicted shared protein–protein interfaces of the nsp16-nsp10 and nsp14-nsp10 interactions (Fig. 2c), the inhibitory peptides targeting both the SARS-CoV-2 nsp16 2′O-MTase and nsp14 ExoN were manually designed. A set of dual-target peptide inhibitors (19 peptides), hereafter coined as overlapping peptides (OLPs) with lengths ranging from 4 to 8 (Table S6), were designed based on the predicted overlapping key interacting residues of nsp10 in SAR-CoV-2, i.e. T39-T47, G69-S72, C77-H80, and K93-Y96. Meanwhile, in a parallel approach, four sets of peptides as hot segments with the significant binding energies were predicted individually for each complex using the Peptiderive server. In total, 22 linear (P-16-5 to P-16-15 and P-14-5 to P-14-15) (Table S7) and 16 cyclic peptides (Table S8) with the highest relative interface scores (percent) were designed. It is remarkable that comparison of the peptides designed by the two methodologies revealed five similar peptide sequences. The OLPs, including OLP-11, OLP-13, OLP-16, OLP-18, and OLP-19, and the peptides designed by Peptiderive to target nsp16, including P-16-5, P-16-6, P-16-7, P-16-8, and P-16-9, have the same sequences. Importantly, only the linear peptides were considered in this study and were further evaluated. To assess the predicted cyclic peptides another research study is required.

### Molecular docking analysis of the designed inhibitory peptides

Molecular docking was used to investigate the binding poses and energies of the designed inhibitory peptides with the target protein. The docking procedure included two steps. In the first step, peptide–protein docking was performed by HPEPDOCK using its local docking algorithm and by specifying the key interacting residues of the target. Based on the best model selection criteria, the docking energy scores of the 36 designed peptides ranged from − 152.081 to − 49.141, and from –226.821 to − 67.438 for docking with nsp16 and nsp14, respectively (Tables S6 and S7). Among OLPs, OLP-13 when the target was nsp16, and OLP-18 when the target was nsp14 showed the lowest docking energy scores (-148.518 and -135.334, respectively). The OLPs with the lowest docking energy scores for both targets were selected. For the second step of docking, 14 out of 36 designed peptides with the most negative docking energy scores, including OLP-13, OLP-16, OLP-17, OLP-18, P-16-11, P-16-12, P-16-13, P-16-14, P-16-15, P-14-11, P-14-12, P-14-13, P-14-14, and P-14-15 were selected.

The results of the peptide–protein docking by HADDOCK, including the HADDOCK score, RMSD from the overall lowest-energy structure, Van der Waals energy, electrostatic energy, desolvation energy, and buried surface area for each peptide-target complex, are reported in Table S9. All the peptides studied interacted with nsp16 at almost the same poses with which nsp10 interacted. Among the OLPs-nsp16 interactions, OLP-18 and OLP-13 showed the lowest HADDOCK scores of − 65.8 ± 4.6 and − 50 ± 2.1 with the large buried surface areas of 1213.3 ± 29.8 and 950 ± 16.9 Å^2^, respectively. P-16-11 and P-16-13 showed the most negative HADDOCK scores among the peptides specifically designed to target the nsp16. Among OLPs-nsp14 interactions, OLP-18 and OLP-13 showed the lowest HADDOCK scores (− 41.1 ± 1.9 and − 39.1 ± 11.3, respectively). The lowest HADDOCK score (− 99.4 ± 2.5) and the largest buried surface area (1918.3 ± 24.4 Å^2^) were predicted for the P-14-15-nsp14 complex. The HADDOCK scores of nsp10 docking with nsp16 and nsp14 were predicted as reference (− 117.1 ± 2 and -123.4 ± 3, respectively).

### Binding free energy analysis

In the next step, the ΔG and K_d_ of the best peptide-target complex model were calculated for each peptide (Table S9). The ΔG of each complex was compared with the ΔG of the reference structure. The ΔG and K_d_ predicted values for the SARS-CoV-2 nsp16-nsp10 were − 12.8 kcal/mol and 4.3 × 10^−10^ M, respectively. Among the peptides designed to target nsp16, P-16-15, P-16-14, P-16-12, OLP-18, and P-16-13, respectively, showed the lowest ΔG values (the most negative, the best affinity). The ΔG and K_d_ predicted values for nsp14 were − 21.6 kcal/mol and 1.4 × 10^−16^ M, respectively. The peptides designed to target nsp14, i.e. P-14-15, P-14-14, P-14-13, P-14-12, and P-14-11, respectively, showed the most negative ΔG values. The ΔG values of OLP-13 and OLP-18 for interactions with nsp14 was the same (-8.9 kcal/mol). The designed peptides with lower K_d_ values had the potential to bind more strongly to their respective protein target. Moreover, Table S9 shows the results of the MM-GBSA free energy decomposition analysis of the peptide-target complexes. Inhibitory peptides of nsp16, including OLP-18, P-16-12, and P-16-13, as well as peptide inhibitors of nsp14, including P-14-15, P-14-14, and P-14-12, showed the most negative MM-GBSA free energies, respectively. The energy contribution of the designed peptides per-residue is listed in Table S9. The lower binding energy of a residue indicates its critical role in the peptide-target interaction. In OLP-13-nsp16, OLP-13-nsp14, OLP-18-nsp16, and OLP-16-nsp14, methionine at position 5 showed the lowest energy relative to other residues of the peptides. Also, the top 10 residues of the target proteins with the lowest energies are reported in Table S9, and the top 5 are shown in Figs. S24–S27. In OLPs-nsp16 interactions, I40, M247, V44, and A83 were the common residues of nsp16 with the lowest energy contributions and, consequently, more critical roles. T25, P24, and F8 of nsp14 were common critical residues (with the lowest energies) in all OLPs-nsp14 interactions (Table S9). Table 2 summarizes the designed inhibitory peptides, their predicted docking scores, and binding energies.

**Table 2:** List of the designed inhibitory peptides targeting nsp16 and nsp14 with their sequences, lengths, HADDOCK scores, binding free energies (ΔG), and MM-GBSA free energies.

Peptide name	Length	Sequence	Target	HADDOCK score	ΔG (kcal/mol)	ΔG MM/GBSA (kcal/mol)
OLP-13 (P-16-6)	6	NCVKML	nsp16	− 50 ± 2.1	− 7.6	− 33.83
OLP-16 (P-16-7)	7	NCVKMLC	nsp16	− 32.3 ± 5.6	− 8.1	− 24.25
OLP-17	7	CVKMLCT	nsp16	− 43.4 ± 1.3	− 6.8	− 31.32
OLP-18 (P-16-8)	8	NCVKMLCT	nsp16	− 65.8 ± 4.6	− 9.4	− 45.51
P-16-11	11	TNCVKMLCTHT	nsp16	− 68.7 ± 1.6	− 9.1	− 37.21
P-16-12	12	TNCVKMLCTHTG	nsp16	− 48.7 ± 3.2	− 9.5	− 46.06
P-16-13	13	TNCVKMLCTHTGT	nsp16	− 68.2 ± 3.4	− 9.3	− 48.32
P-16-14	14	TNCVKMLCTHTGTG	nsp16	− 67.7 ± 4	− 9.7	− 36.44
P-16-15	15	ITNCVKMLCTHTGTG	nsp16	− 51.9 ± 5.5	− 9.9	− 40.19
OLP-13 (P-16-6)	6	NCVKML	nsp14	− 39.1 ± 11.3	− 8.9	− 33.84
OLP-16 (P-16-7)	7	NCVKMLC	nsp14	− 32.7 ± 4.1	− 8.7	− 30.44
OLP-17	7	CVKMLCT	nsp14	− 26.2 ± 3.5	− 8.7	− 35.47
OLP-18 (P-16-8)	8	NCVKMLCT	nsp14	− 41.1 ± 1.9	− 8.9	− 34.08
P-14-11	11	STVLSFCAFAV	nsp14	− 54.7 ± 1.3	− 9.6	− 39.09
P-14-12	12	NSTVLSFCAFAV	nsp14	− 59.3 ± 3.5	− 11	− 41.36
P-14-13	13	NSTVLSFCAFAVD	nsp14	− 66.9 ± 4.3	− 11.6	− 38.81
P-14-14	14	PANSTVLSFCAFAV	nsp14	− 82.3 ± 3.9	− 11.9	− 46.39
P-14-15	15	VPANSTVLSFCAFAV	nsp14	− 99.4 ± 2.5	− 13.4	− 50.06

### Peptide–protein interactions analysis

Following the docking and binding energy analyses of the designed peptides, the interactions of the designed peptides in Table 2 with the target protein were analyzed to gain better insights into the key interacting residues and their interaction types. The residues of the target protein at the interface involved in each peptide-target interaction are listed in Table S9. The residues of nsp16, including I40, M41, V44, T48, V78, A79, P80, A83, V84, Q87, V104, S105, D106, L244, and M247, were common residues that were involved in all the OLPs-nsp16 interactions. For P-16-11, P-16-12, P-16-13, P-16-14, and P-16-15, K38, G39, I40, V44, G77, V78, A79, A83, V84, Q87, V104, S105, D106, L244, and M247 of nsp16 were common residues at the interfaces of peptide-nsp16 complexes. The common interacting residues of nsp14 in all OLPs-nsp14 interactions were F8, A23, Q22, P24, T25, D126, T127, and T131. Also, V4, L7, F8, P20, T21, A23, P24, T25, H26, C39, V40, D41, F60, K61, M62, N63, Y64, and I201 of nsp14 were common for mediating the interactions of P-14-11, P-14-12, P14-13, P14-14, and P-14-15 with nsp14.

**Figure 4:**
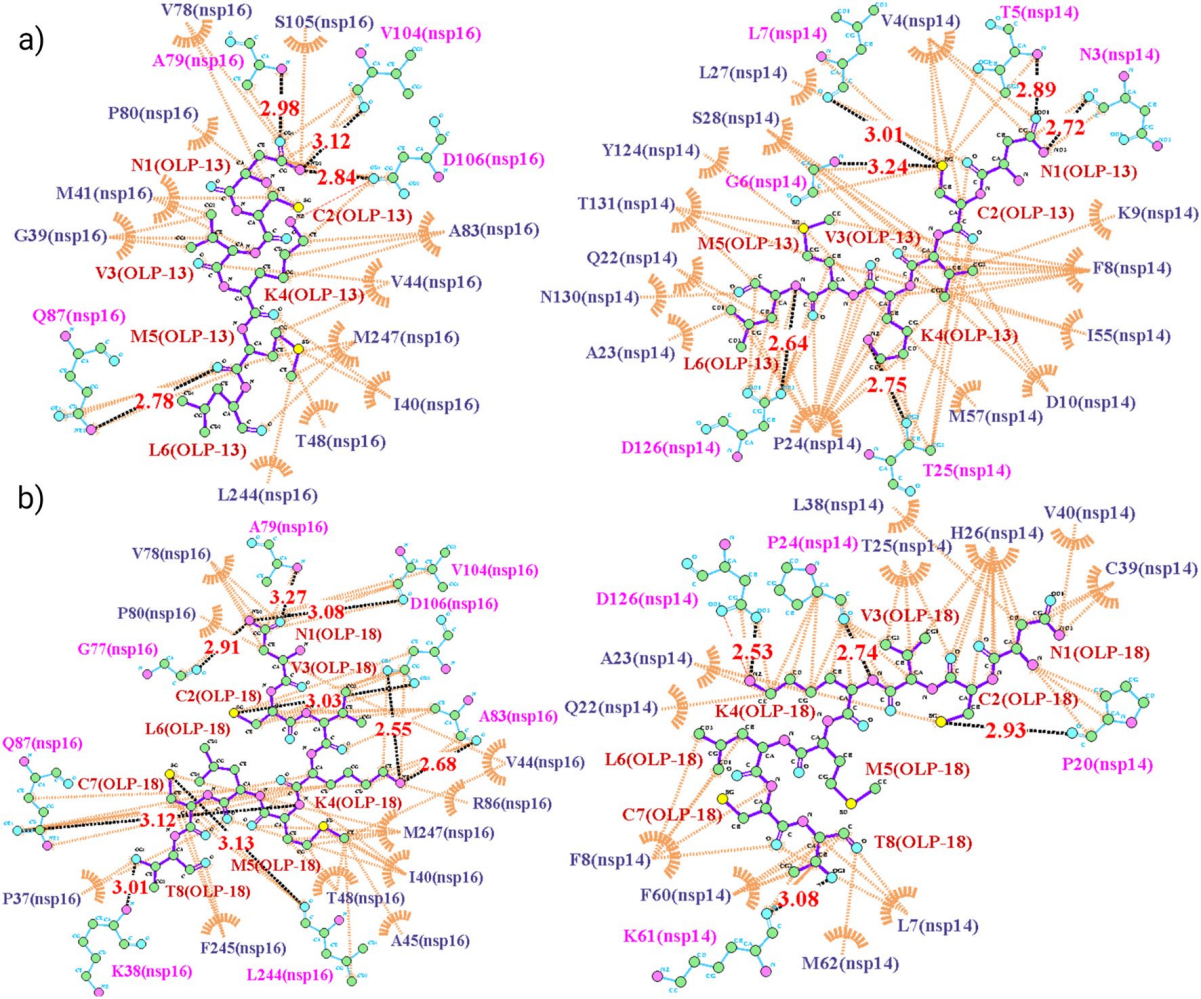
The maps of peptide–protein interactions for the OLP-13 and OLP-18 peptides and their targets (nsp16 and nsp14). (**a**) Peptide–protein interactions of OLP-13 with nsp16 (left) and nsp14 (right) as the target proteins. (**b**) Peptide–protein interactions of OLP-18 with nsp16 (left) and nsp14 (right) as the target proteins. The black dashed lines and the brown arcs with spokes represent the hydrogen bonds and the hydrophobic contacts, respectively.

The maps of interactions for OLP-13 and OLP-18 with their targets (nsp16 and nsp14) indicated that the hydrophobic interactions were the most abundant interactions in these OLP-target complexes (Fig. 4). The N1 of OLP-13 participated in three H-bonds with A79, D106, and V104 of nsp16. In addition, the M5 of OLP-13 formed an H-bond with Q87 of nsp16. The N1 of OLP-13 formed two H-bonds with T5 and N3 of nsp14. Also, C2 of OLP-13 participated in two H-bonds with G6 and L7 of nsp14. Other H-bonds were found between K4 and L6 of OLP-13 and T25 and D126 of nsp14, respectively (Fig. 4a). The N1 of OLP-18 formed three H-bonds with A79, G77, and V104 of nsp16. C2 and K4 of OLP-18 participated in H-bonds with D106 of nsp16. K4, M5, C7, and T8 of OLP-18 formed H-bonds with A83, Q87, L244, and K38 of nsp16, respectively. In the OLP-18-nsp14 interaction, K4 formed two H-bonds with D126 and P24 of nsp14. T8 and C2 were involved as donors in the H-bonds with K61 and P20 of nsp14, respectively (Fig. 4b). The maps of interactions for the other peptides are shown in Figs. S28–S31. The hydrophobic interactions were predominant in these peptide–protein complexes. Moreover, the fluctuations of residues in each target protein-peptide complex during the simulations are shown in Fig. S32.

### MM-PBSA analysis of the best designed peptides

**Figure 5:**
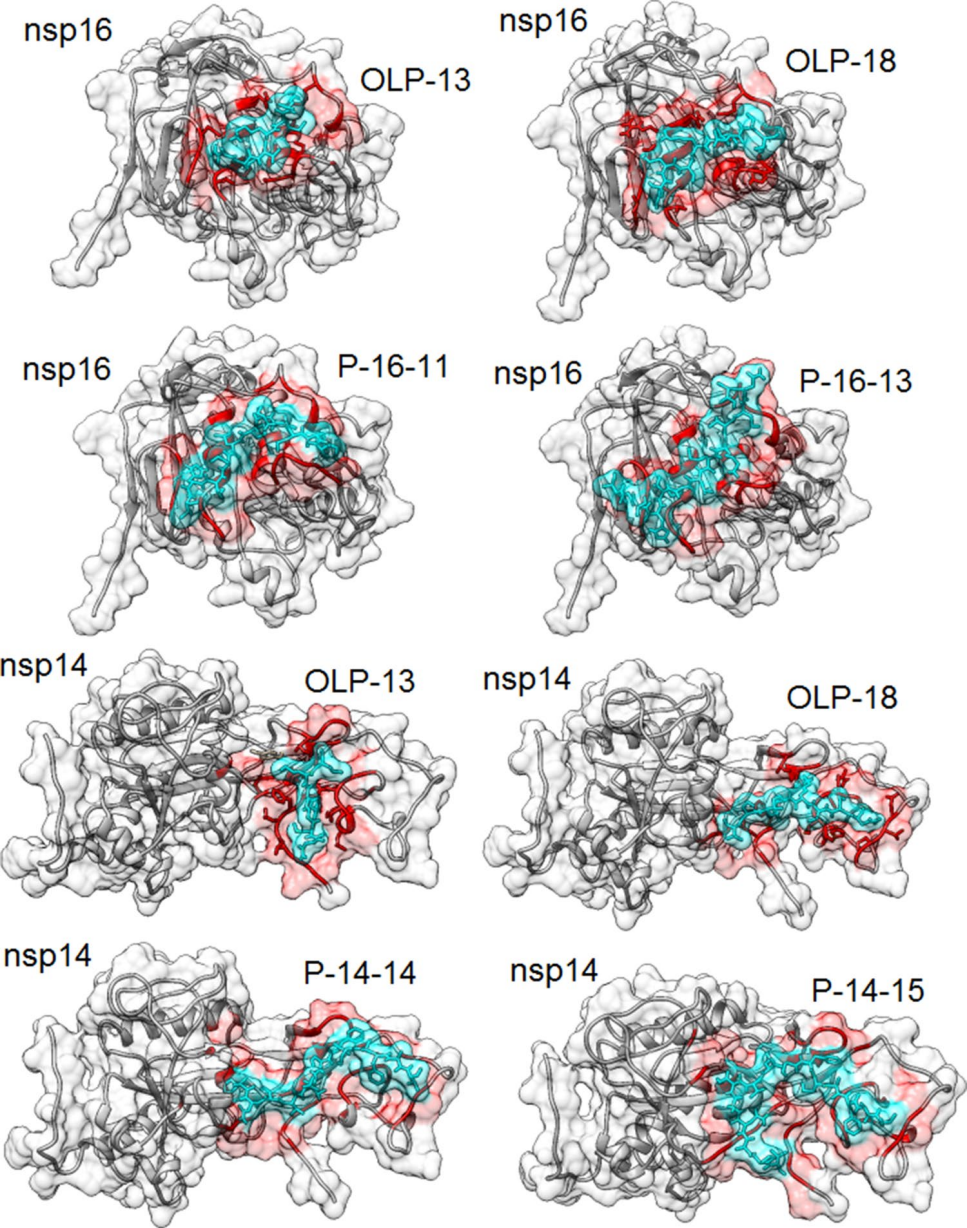
3D structure representations of the best scoring designed peptides to the target proteins (nsp16 and nsp14). The key interacting residues and peptides are colored in red and turquoise, respectively.

Among the designed peptides, OLP-13, OLP-18, P-16-11, P-16-13, P-14-14, and P-14-15 with the most negative HADDOCK scores, were selected for further analysis. (Fig. 5). These best scoring peptides were subjected to further evaluation following 50 ns MD simulations using the MM-PBSA method to investigate the binding free energies and select the most promising peptides. The MM-PBSA results are presented in Table 3. The negative values of ΔG_binding_ from MM-PBSA for all the analyzed peptides indicated their favorable binding affinities to the respective target(s). P-16-11 and P-14-14 showed the lowest ΔG_binding_ values for nsp16 and nsp14 (− 30.4218 and − 33.6353 kcal/mol, respectively). Both OLP-13 and OLP-18 showed better binding affinity, with more favorable ΔG_binding_ to nsp16 (− 22.4568 and − 24.1671 kcal/mol) than nsp14 (− 17.3694 and − 19.6176 kcal/mol).

**Table 3:** The binding free energies of the designed peptides calculated by MM-PBSA method.

Peptide name	Peptide sequence	Target	ΔG_binding_ (kcal/mol)	EEL (kcal/mol)	EPB (kcal/mol)	VDWAALS (kcal/mol)
OLP-13	NCVKML	nsp16	− 22.4568	− 141.3623	167.5499	− 38.9689
OLP-18	NCVKMLCT	nsp16	− 24.1671	− 148.6379	171.4235	− 40.6208
P-16-11	TNCVKMLCTHT	nsp16	− 30.4218	− 204.2514	226.6015	− 45.6772
P-16-13	TNCVKMLCTHTGT	nsp16	− 27.0281	− 48.5884	80.9416	− 51.4337
OLP-13	NCVKML	nsp14	− 17.3694	− 148.9625	150.3642	− 15.6923
OLP-18	NCVKMLCT	nsp14	− 19.6176	− 152.1808	157.6861	− 16.6782
P-14-14	PANSTVLSFCAFAV	nsp14	− 33.6353	− 57.5389	75.5983	− 45.2555
P-14-15	VPANSTVLSFCAFAV	nsp14	− 32.2159	− 56.0246	70.6485	− 42.3789

### Optimization of the designed peptides

**Figure 6:**
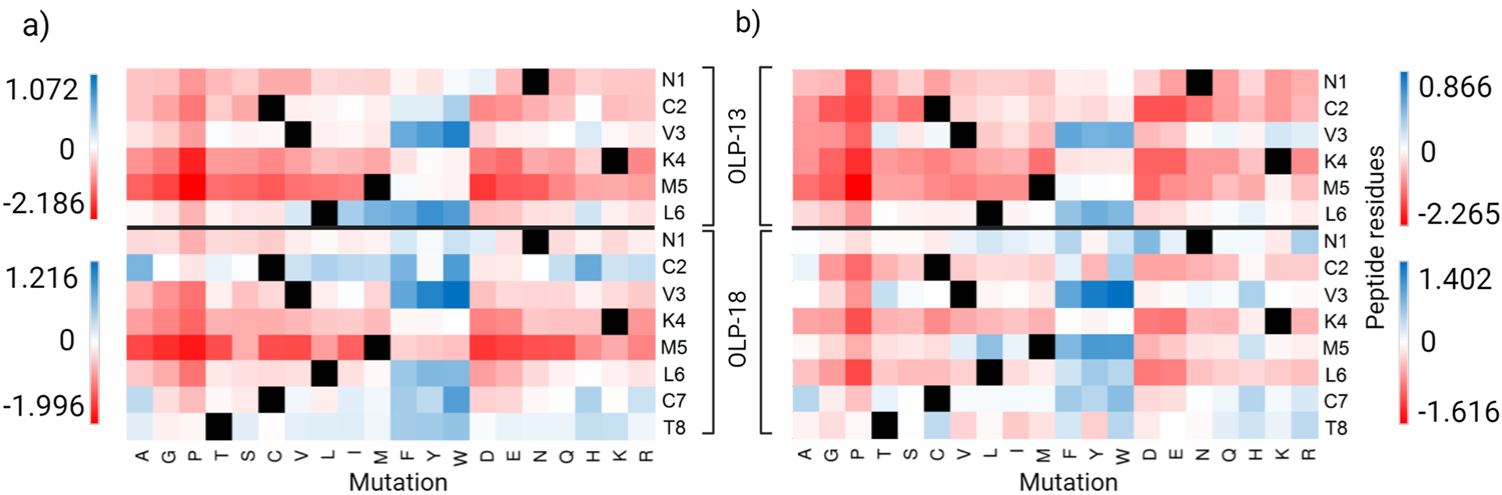
Heat maps representing in silico saturation mutagenesis analysis of OLP-13 and OLP-18 inhibitory peptides. (a) OLP-13-nsp16 and OLP-18-nsp16 interactions, and (b) OLP-13-nsp14 and OLP-18-nsp14 interactions. Mutations with improving (positive ΔΔGAffinity) and decreasing (negative ΔΔGAffinity) impacts on the peptide-target binding affinity are shown in blue and red, respectively.

Following the two steps of molecular docking and then binding free energy analysis by MM-PBSA, the six best scoring peptides (Fig. 5) were selected as lead peptide sequences for comprehensive in silico saturation mutagenesis analysis to identify the peptides with improved binding affinities by investigating the effects of each mutation on the designed peptide-target binding affinity. In this regard, a large library of peptide inhibitors with 1539 new peptide sequences was generated by mutating each residue of six lead sequences to the other 19 amino acids (Table S10). The positive ΔΔG_Affinity_ of the mutant relative to the wild-type indicated an improved impact of this mutation on the peptide-target affinity. For OLP-13 and OLP-18, 17.5% and 34.8% of substitutions showed positive ΔΔG_Affinity_ with improving impacts on peptide-nsp16 interactions, respectively. However, only 0.06% and 0.07% showed considerable ΔΔG_Affinity_ (> 0.5 kcal/mol). For OLP-13-nsp14 and OLP-18-nsp14 interactions, 15% and 40% of mutations showed improving impacts with positive ΔΔG_Affinity_ respectively. Mutation of the peptide residues to phenylalanine, tryptophan, and tyrosine showed the highest improving impacts of these variations on peptide-target affinity with the most positive ΔΔG_Affinity_ (blue color). However, these amino acids decreased the predicted binding affinity at some positions, like substitutions at N1, K4, and M5 of OLP-13 or K4 and M5 of OLP-18 in the interaction with nsp16 (Fig. 6a). Mutating C2 and K4 of OLP-13 in complex with nsp14 to all the other 19 amino acids resulted in negative ΔΔG_Affinity_ (red colors) with decreasing impacts, demonstrating the critical roles of these residues in the OLP-13-nsp14 interaction (Fig. 6b). Heat maps representing in silico saturation mutagenesis of other lead peptides are shown in Fig. S33. Moreover, to obtain the optimized inhibitory peptides, the physicochemical, pharmacokinetic, and toxicity properties of the designed peptides were predicted. These properties are given in detail in Table S11. Allergenicity prediction classified the designed peptides as probable allergens and probable non-allergens. In addition, toxicity analysis classified all the designed peptides as non-toxic, except P-16-11, P-16-12, and P-16-13.

### Conservation analysis to identify the pan-coronavirus peptide inhibitors

To identify the pan-coronavirus peptide inhibitors, the conservation of the target residues that were identified to interact with the designed peptides was analyzed among CoVs. Multiple sequence alignment analysis of the nsp16 from seven human CoVs, i.e. SARS-CoV-2, SARS-CoV, MERS-CoV, HCoV-OC43, HCoV-HKU1, HCoV-NL63, HCoV-229E (Fig. S34a), revealed that the residues of nsp16, including G39, A79, P80, V84, V104, S105, and D106 that interact with peptides, were identical in these CoVs. I40, M41, T48, G77, V78, A83, Q87, L244, and M247 of nsp16 were similar residues. I40 was replaced with cysteine in both HCoV-OC43 and HCoV-HKU1 nsp16, and with valine in MERS-CoV nsp16. In nsp16 of MERS-CoV, M41, T48, V78, and M247 were replaced with histidine, methionine, isoleucine, and leucine, respectively. In HCoV-HKU1, G77 was replaced with glutamic acid. A83 was replaced with serine in MERS-CoV, and with threonine in HCoV-NL63. In nsp16 of HCoV-NL63 and HCoV-229E, L244, and M247 were replaced with valine and leucine, respectively. Next, the conservation of the nsp16-nsp10 complex among four CoVs (SARS-CoV-2, SARS-CoV, MERS-CoV, and HCoV-OC43), was analyzed (Table S12). G39, T48, V84, S105, and D106 of nsp16 were the most highly conserved residues with a conservation score of 9 (dark magenta). Other key residues of nsp16, including I40, M41, V44, G77, V78, A79, P80, Q87, V104, and L244, were well conserved, with conservation scores of 6–8 (pink). Among the investigated residues, K38 was a highly variable residue with a score of 1 (turquoise). A83 and M247 of nsp16 were intermediately conserved or variable (from white to turquoise) (Fig. S34b). Multiple sequence alignment analysis of nsp14 among the seven CoVs showed that L7, F8, P20, A23, V40, and F60 of nsp14 were identical residues. V4, Q22, P24, T25, C39, D41, K61, M62, N63, T127, and I201 of nsp14 were similar residues (Fig. S35a). L7, P20, A23, T25, and F60 were the highly conserved residues with a score of 9 in the nsp14. F8, C39, V40, D41, K61, M62, N63, and T127 were well conserved (with scores of 6–8). V4, Q22, P24, H26, Y64, D126, T131, I201, D41 of nsp14 were variable residues. T21 was an intermediate residue (with a score of 5) (Fig. S35b; Table S12). Also, the results of the conservation analysis for the nsp10 protein across CoVs are shown in Fig. S36 and Table S12.

## Discussion

In this study, we considered the PPIs in RTC, focusing particularly on the roles of nsp14 and nsp16 and their common critical cofactor, nsp10. Interactions of nsp10 with both nsp14 and nsp16 demonstrated its central activatory role in the survival and pathogenesis of CoVs (Fig. 1a). Targeting these two PPIs is important in several aspects. The large size of the genome of CoVs justifies the requirement for ExoN activity. The capability of RdRp to correctly select nucleotides is insufficient to provide replication fidelity, confirming the importance of nsp14 ExoN proofreading activity^74^. The interaction of nsp14 with nsp10 strengthens its ExoN activity up to 35-fold in vitro^75^. In addition, interference with the last step of the capping process in SARS-CoV-2 (Fig. 1b) has the potential to block replication, translation, and viral escaping mechanisms. Studies have showed the inhibition of the enzymatic activity of nsp16 in CoVs by peptides derived from the nsp10 of SARS-CoV^26^ and mouse hepatitis virus (MHV)^27^.

To design the peptide inhibitors at the starting point of the workflow (Fig. S3), the key residues mediating the interactions of nsp10 with nsp14 ExoN and nsp16 2'-O-MTase were predicted (Fig. 2 and Table 1). All key interacting residues of nsp14 were spread towards its ExoN N-terminal domain. This indicates that nsp10 may not participate in activating N7-MTase and may also reveal the separate roles of the nsp14 domains. These results were in consonance with the studies that showed the independent function of nsp14 ExoN and N7-MTase by the hydrolysis analysis^76^, as well as the biochemical analysis^77^. Moreover, the results of the MD simulations showed that a hinge region separated the two domains of nsp14. In addition, higher flexibilities of these regions may reflect their roles in mediating PPIs. Owing to the dynamic nature of the loop, the loops at both the N-terminal and C-terminal of nsp10 showed greater flexibility compared to other residues. Importantly, comparison of the key interacting residues in these two PPIs indicated that 24 residues in nsp10 interacted with both nsp16 and nsp14 in SARS-CoV-2 (Fig. 2c). Mutagenesis and BRET analyses have also been used to map nsp10 overlapping interactions in SARS-CoV^78^. The unique feature of these overlapping key interacting residues serves as a window for targeting two key proteins (nsp16 and nsp14) with one inhibitor (OLP). Bouvet et al.^78^ showed that K43A and Y96F mutations in nsp10, named “mutator phenotype” rise in SARS-CoV mutations. In addition, nsp10 mutations decreased or put an end to ExoN activation^75^. The results of CAS analysis (Fig. S9) are consistent with these reports. Studies have shown that nsp14 ExoN inactivation in CoVs mitigates the fidelity of replication up to 20-fold and the absence of ExoN activity has improved the sensitivity to lethal mutagenesis in the presence of RNA mutagenesis^79^. The explanation of similarities and distinctions of the interfacial and hotspot residues between SARS-CoV-2 and other CoVs (Figs. S6–S8) will contribute to the future preparation for possible new emerging CoVs. In addition, this study is the first that predicted the key interacting residues (Fig. S8), and their map of interactions (Fig. S17) and also performed the conservation analysis (Table S12) on the recently reported structure of the HCoV-OC43 nsp16-nsp10 complex^33^.

The large interface areas were buried upon the formation of the nsp14-nsp10 and nsp16-nap10 complexes. Therefore, these large interface areas with a large number of key interacting residues demonstrated the targeting of these PPIs by interfering peptides instead of small molecules. Small molecules face challenges in participating in these large areas to compete with the interaction of the original partner (nsp10). Furthermore, maps of the residue interactions revealed the complicated hydrogen bonding and hydrophobic interactions (Fig. S14), implying that targeting these hydrophobic interactions with small molecules may be more challenging and even improbable. On the other hand, the smaller size of the designed peptides relative to the nsp10 may provide a benefit in the competitive binding due to being effectively greater in the concentration at the interface and consequently improving their affinity.

Next, based on the results of the PPIs analysis, the peptide inhibitors were computationally designed to target the RNA capping and proofreading mechanisms in SARS-CoV-2 (Tables S6–S8). Then, the designed peptides were evaluated (Table S9). Among OLPs, OLP-18 (NCVKMLCT) and OLP-13 (NCVKML) showed the lowest docking scores and binding energies, engaged a large number of critical target residues in the interactions, showed proper binding poses, and acceptable properties. Peptide–protein interactions were mostly hydrophobic (Fig. 4), similar to the native interactions (Fig. S14). There was no general correlation between peptide length and binding free energy for the designed peptides when nsp16 was the target protein. However, the binding free energies of the peptides when nsp14 was the target showed a direct correlation with the peptide length (Table 2). Moreover, the HADDOCK scores of all analyzed peptides were higher than those of native interactions. Thus, to identify the peptides that bind more strongly with near or lower ΔG_binding_ than the native partner (nsp10), the designed peptides were optimized by in silico saturation mutagenesis analysis and a new library of peptides was generated (Table S10). Using this computational mutational analysis, the substitutions with the most positive ΔΔG_Affinity_ were revealed (Figs. 6 and S33). These mutants, as the shortlisted candidates, launch a new round of complementary computational studies by employing the methods described in this study to evaluate the newly designed peptides. Furthermore, conservation analysis revealed the conservation of the target residues that interacted with the designed peptides (Figs. S34 and S35; Table S12). Therefore, according to the conserved nature of the target residues, the designed peptides have the potential to function as pan-coronavirus inhibitors and may be useful for possible newly emerged CoVs. In addition, the reported cyclic peptides in this study are worthy of further computational research.

**Figure 7:**
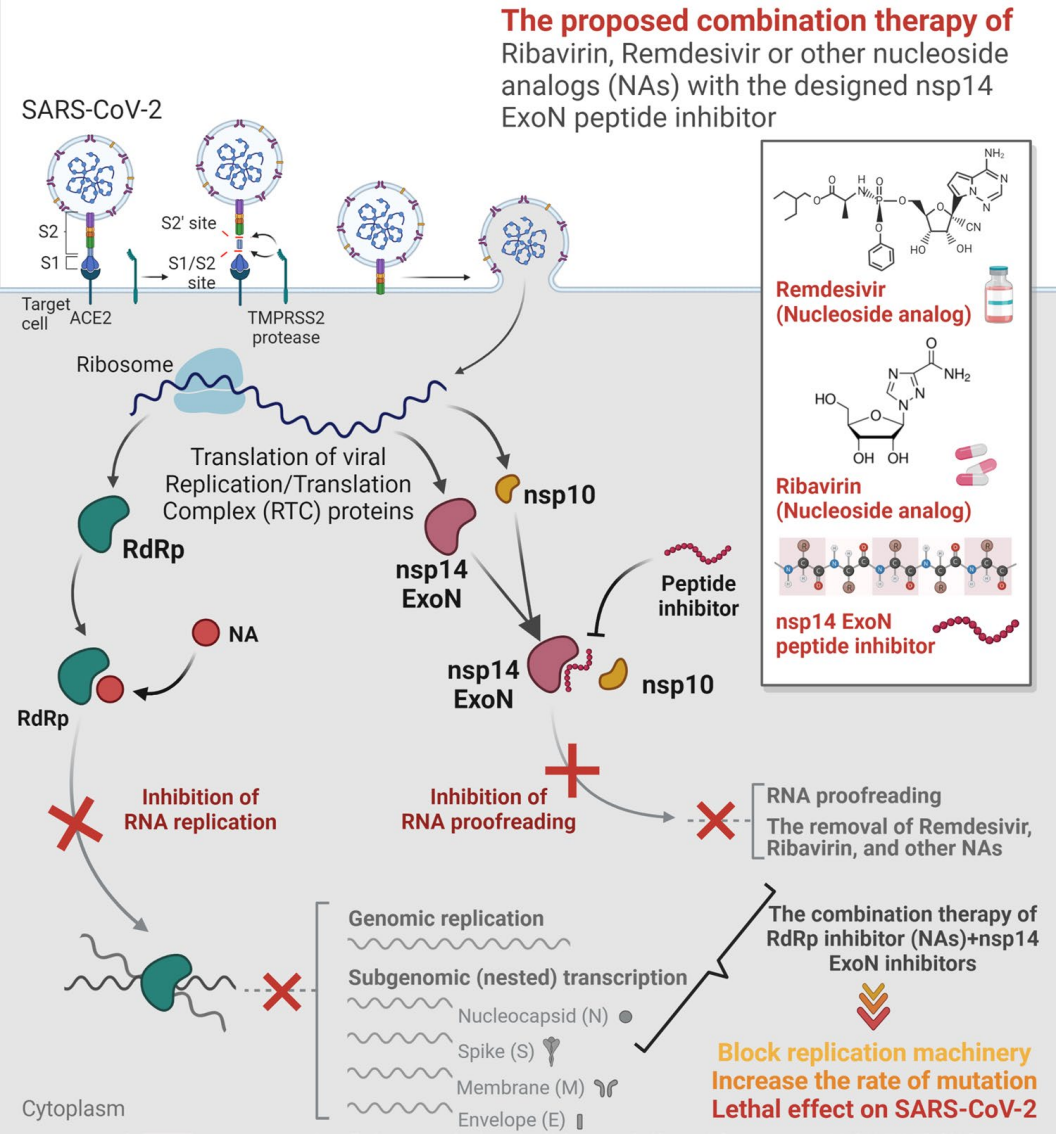
Schematic depicting the proposed strategy for combination therapy of nucleoside analogs (NAs) with the nsp14 ExoN designed peptide inhibitors. The combination therapy of Ribavirin, Remdesivir or other NAs with the nsp14 ExoN peptide inhibitors designed in this study is proposed to complete the NAs antiviral effects to block both RNA replication and RNA proofreading mechanisms.

S-adenosyl-l-homocysteine as the co-product of the MTase reaction, sinefungin, and aurintricarboxylic acid (ATA) were identified as nsp16 substrate binding inhibitors of CoVs. SAM is used as a methyl donor by various host cellular MTases. The SAM-binding site inhibitors impact on the host 2'-O-MTase activity, leading to induced cytotoxic effects^80^. In this study, the unique viral interaction site was targeted. Thus, the designed peptides may be more selective and accomplished than SAM mimetics. To the best of our knowledge, no nsp14 peptide inhibitor for CoVs is currently available, and this study represents the first computational effort. Excising Ribavirin 5'-monophosphate as a guanosine analog from RNA substrates by the nsp14-nsp10 complex explains its poor activity against CoVs. In the mutator phenotype of nsp14 with ExoN inactivation, Ribavirin showed 200^81^ and Remdesivir showed 4.5 fold increased sensitivity^82^. Alongside these studies, here the combination therapy of nucleoside analogs (NAs) with this first-generation of nsp14 designed peptide inhibitors is suggested (Fig. 7). Irrespective of the peptide limitations, this proposed strategy is worth exploring. In addition, the proposed therapy is anticipated to complement the antiviral effects of NAs and might trigger inhibition of both RNA replication and RNA proofreading mechanisms. Moreover, when Remdesivir resistance develops as a result of RdRp mutations, it is envisaged that the designed peptides might likely boost Remdesivir's effectiveness without raising the risk of resistance^83^. However, some important aspects of the molecular and biochemical points of view are still unknown, and the designed peptides require more computational and experimental studies. Further research is required to study the activities of the proposed peptides in vitro and in vivo as well as to evaluate their specificity, efficacy, and pharmacokinetics. Additionally, when considering the designed peptides as prospective therapeutic agents for COVID-19 patients, it is suggested to investigate their delivery system, particularly the preferred strategies of administration, such as subcutaneous or intravenous, similar to the peptides recently approved by the FDA^84^. Also, in order to evade host immunity, the peptides could be generated in COVID-19 patients using in vitro transcribed (IVT) mRNA^85^.

## Conclusion

CoVs have been the cause of human respiratory syndromes for many years. The COVID-19 global pandemic has led to many health care and economic complications in recent years. This highlights the importance of studying the structural and molecular basis of SARS-CoV-2 with high infection rate for the development of vaccines and therapeutics. Besides receptor recognition, studying each point of the RTC and remarkably PPIs of nsps leads to the design of specific interfering antivirals, like peptide inhibitors, with the potential to target a unique viral interaction site. The overlapping of protein–protein interfaces between nsp16-nsp10 with capping activity and nsp14-nsp10 as a guardian of replication with proofreading activity makes these PPIs promising targets for designing antivirals for CoVs. Disturbing the SARS-CoV-2 cap-1 formation step has the potential to disturb 2′-O-MTase activity and possibly make viral mRNAs more likely to be influenced by key protein sensors to initiate the earliest immune responses against the viral infection. Cellular sensors, such as toll-like receptors (TLR), retinoic acid-inducible gene I (RIG-I), and melanoma differentiation-associated protein 5 (MDA5) recognize RNAs lacking 2′O-methyl at the first nucleotide and induce signaling cascades that result in the expression and release of cytokines and type I interferon^16^. This study provides comprehensive information about the key residues mediating the interactions of nsp10 as a dual cofactor with nsp16 2′O-MTase and nsp14 ExoN in the SARS-CoV-2 and other CoVs, that are useful for designing new therapeutics to combat current or future CoVs. The PPIs results showed that 24 interacting residues are common at the PPIs interfaces of these complexes. Thus, OLPs with dual inhibitory potencies were designed, evaluated and then optimized. The predicted conservation of target key residues offers a new direction for designing pan-coronavirus inhibitors. Efforts to successfully develop and optimize the peptides from the current study may turn them into a new generation of antiviral candidates for the treatment of COVID-19 or future possible related outbreaks.

## Supplementary Information

Supplementary Information 1.

Supplementary Information 2.

## Data Availability

All data generated or analyzed during this study are included in this published article and its supplementary information files.
