# Supplementary material for: Analysis of critical protein–protein interactions of SARS-CoV-2 capping and proofreading molecular machineries towards designing dual target inhibitory peptides
Source: Sci Rep. 2023 Jan 7;13:350. doi: 10.1038/s41598-022-26778-8 (PMC9825083; doi:10.1038/s41598-022-26778-8)
Supplement: Supplementary file 1 — Supplementary Information 1. [file 41598_2022_26778_MOESM1_ESM.pdf]

Supplementary Information File 1

Analysis of critical protein-protein interactions of SARS-CoV-2 capping and proofreading molecular machineries towards designing dual target inhibitory peptides

Fatemeh Arabi-Jeshvaghani[1], Fatemeh Javadi-Zarnaghi[1, *], Mohamad Reza Ganjalikhany[1, *]

[1]Department of Cell and Molecular Biology & Microbiology, Faculty of Biological Science and Technology, University of Isfahan, Isfahan, Iran

Email: fa.javadi@sci.ui.ac.ir, m.ganjalikhany@sci.ui.ac.ir

Table of contents

Figure S1: Schematic depiction of the genomic organization of SARS-CoV-2.
Figure S2: Schematic illustration of an overview of the study.
Figure S3: The workflow of the study.
Figure S4: Secondary structure plot for the SARS-CoV-2 nsp16-nsp10 complex.
Figure S5: Secondary structure plot for the SARS-CoV-2 nsp14-nsp10 complex.
Figure S6: Analysis of the SARS-CoV nsp16-nsp10 protein-protein interface and hotspot residues.
Figure S7: Analysis of the MERS-CoV nsp16-nsp10 protein-protein interface and hotspot residues.
Figure S8: Analysis of the HCoV-OC43 nsp16-nsp10 protein-protein interface and hotspot residues.
Figure S9: The results of computational alanine scanning (CAS) calculations for the predicted key interacting residues of the SARS-CoV-2 nsp16-nsp10 and nsp14-nsp10 complexes.
Figure S10: Interactions between wild-type (V42, L45, and Y96 of nsp10) and mutant (alanine) residues and their nearby residues in the nsp16-nsp10 complex.
Figure S11: Interactions between wild-type (V84, D106, and V44 of nsp16) and mutant (alanine) residues and their nearby residues in the nsp16-nsp10 complex.
Figure S12: Interactions between wild-type (F16, H80, and Y96 of nsp10) and mutant (alanine) residues and their nearby residues in the nsp14-nsp10 complex.
Figure S13: Interactions between wild-type (F8, P24, and F60 of nsp14) and mutant (alanine) residues and their nearby residues in the nsp14-nsp10 complex.
Figure S14: Two-dimensional residue interaction maps for the SARS-CoV-2 nsp16-nsp10 and nsp14-nsp10 complexes.
Figure S15: Two-dimensional residue interaction map for the SARS-CoV nsp16-nsp10 complex.
Figure S16: Two-dimensional residue interaction map for the MERS-CoV nsp16-nsp10 complex.
Figure S17: Two-dimensional residue interaction map for the HCoV-OC43 nsp16-nsp10 complex.
Figure S18: Distance range and property contact maps for the SARS-CoV-2 nsp16-nsp10 and nsp14-nsp10 complexes.
Figure S19: Heat map for residue-residue pairwise interaction energies (IEs) between the predicted key interacting residues of nsp10 and nsp16 in the SAR-CoV-2 nsp16-nsp10 complex.

Figure S20: Heat map for residue-residue pairwise interaction energies (IEs) between the predicted key interacting residues of nsp10 and nsp14 in the SAR-CoV-2 nsp14-nsp10 complex.
Figure S21: Node centrality parameters in the network, including degree centrality (DC), betweenness centrality (BC), and closeness centrality (CC) of the predicted key interacting residues of the SARS-CoV-2 nsp16-nsp10 complex.
Figure S22: Node centrality parameters in the network, including degree centrality (DC), betweenness centrality (BC), and closeness centrality (CC) of the predicted key interacting residues of the SARS-CoV-2 nsp14-nsp10 complex.
Figure S23: 3D networks of the residue-residue interactions for the SARS-CoV-2 nsp16-nsp10 and nsp14-nsp10 complexes.
Figure S24: The top five nsp16 residues with the greatest energy contributions (the lowest energy) in the interactions of OLP-13, OLP-16, OLP-17, OLP-18 and nsp16 as target protein.
Figure S25: The top five nsp16 residues with the greatest energy contributions (the lowest energy) in the interactions of P-16-11, P-16-12, P-16-13, P-16-14, P-16-15 and nsp16 as target protein.
Figure S26: The top five nsp14 residues with the greatest energy contributions (the lowest energy) in the interactions of OLP-13, OLP-16, OLP-17, OLP-18 and nsp14 as target protein.
Figure S27: The top five nsp14 residues with the greatest energy contributions (the lowest energy) in the interactions of P-14-11, P-14-12, P-14-13, P-14-14, P-14-15 and nsp14 as target protein.
Figure S28: Two-dimensional residue interaction maps of OLP-16 and OLP-17 peptides and the targets (nsp16 and nsp14).
Figure S29: Two-dimensional residue interaction maps for P-16-11, P-16-12, P-16-13, P-16-14, P-16-15 and nsp16 as target protein.
Figure S30: Two-dimensional residue interaction maps for P-14-11, P-14-12, P-14-13 and nsp14 as target protein.
Figure S31: Two-dimensional residue interaction maps for P-14-14, P-14-15 and nsp14 as target protein.
Figure S32: RMSF plots of the target-peptide complexes.
Figure S33: Heat maps representing the *in silico* saturation mutagenesis of the designed peptides.
Figure S34: Conservation analysis of the nsp16 protein across coronaviruses.
Figure S35: Conservation analysis of the nsp14 protein across coronaviruses.
Figure S36: Conservation analysis of the nsp10 protein across coronaviruses.

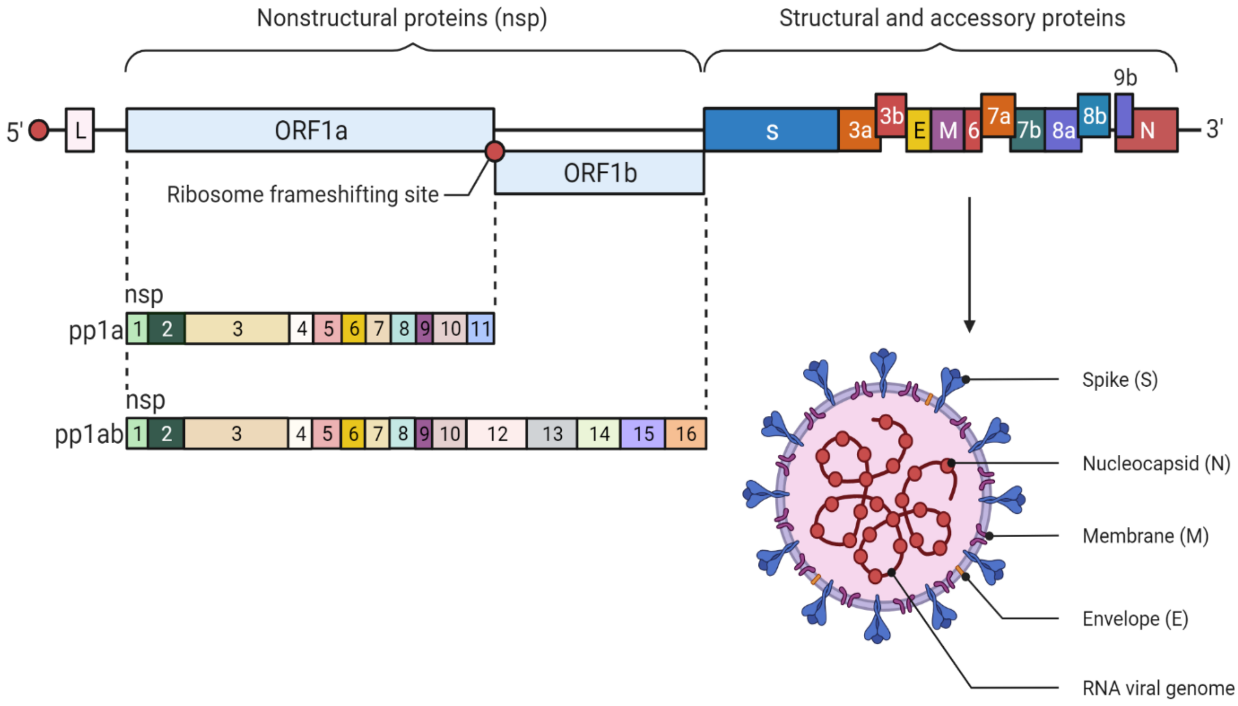

Figure S1: Schematic depiction of the genomic organization of SARS-CoV-2. SARS-CoV-2 is an enveloped spherical betacoronavirus with an approximately 30 kb single-stranded, positive-sense, non-segmented, 5′-capped, and 3′-polyadenylated genome. The open reading frames (ORFs) at the 5′ end of the genome encode nonstructural proteins (nsps) nsp1 through nsp16. The structural and accessory proteins are encoded by the ORFs at the 3′ end of the genome. Created with BioRender (https://biorender.com/). Adapted from "Coronavirus Replication Cycle", by BioRender.com (2022). Retrieved from https://app.biorender.com/biorender-templates.

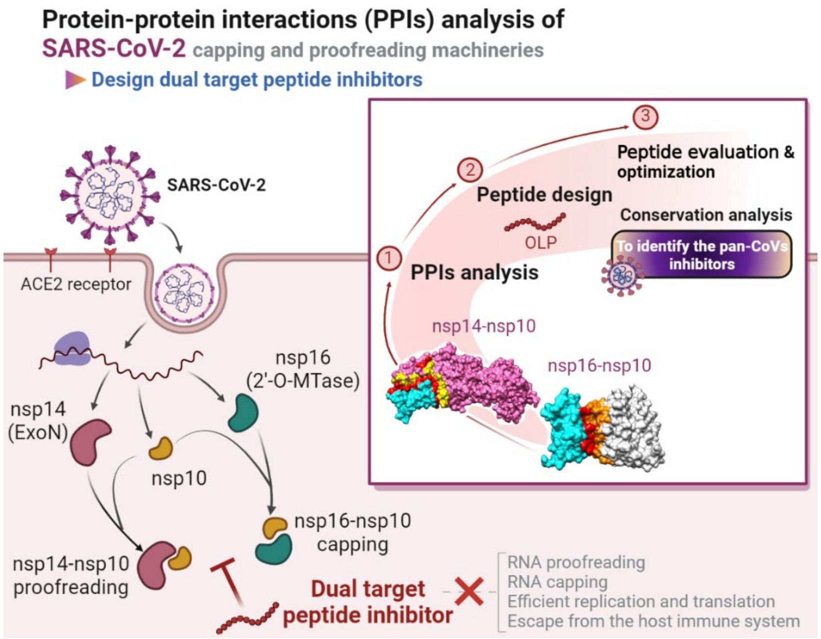

Figure S2: Schematic illustration of an overview of the study. Created with BioRender (https://biorender.com/). Adapted from "Remdesivir: Potential Repurposed Drug Candidate for COVID-19 (Portrait)" by BioRender.com (2022). Retrieved from https://app.biorender.com/biorender-templates.

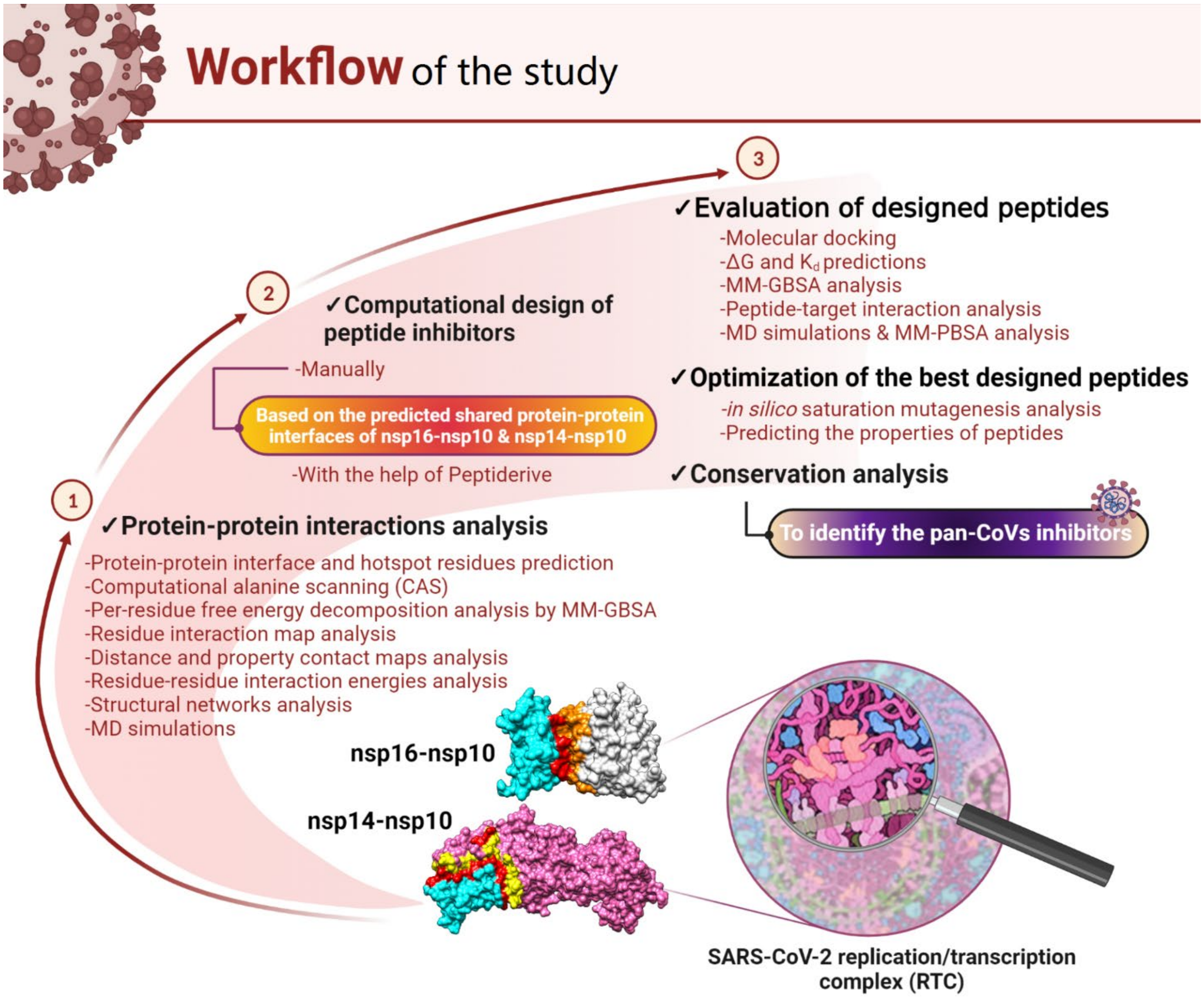

Figure S3: Workflow of the study. The methods used in this study for protein-protein-interactions (PPIs) analysis of the nsp16-nsp10 and nsp14-nsp10 interactions and then peptide inhibitor design, evaluation, and optimization. Created with BioRender (https://biorender.com/). Adapted from "From the Human Body to Microfluidics", by BioRender.com (2022). Retrieved from https://app.biorender.com/biorender-templates. Acknowledgment: David S. Goodsell, RCSB Protein Data Bank; doi: 10.2210/rcsb_pdb/goodsell-gallery-023. Integrative illustration for coronavirus outreach (2020) PLoS Biol 18: e3000815 doi: 10.1371/journal.pbio.3000815.

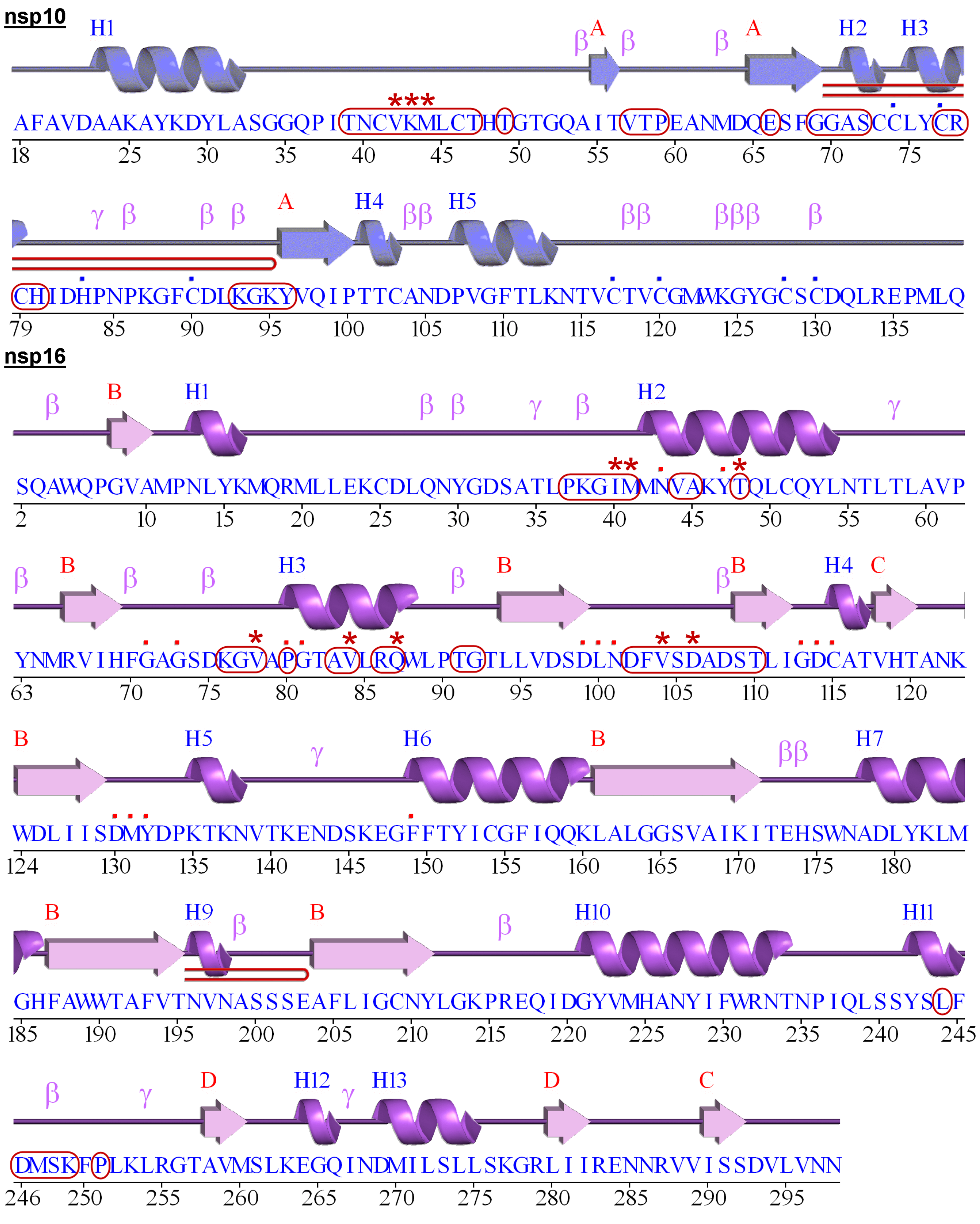

Figure S4: Secondary structure plot of the SARS-CoV-2 nsp16-nsp10 complex. The red boxes denote the interfacial residues. The hotspot residues are indicated with red asterisks.

nsp10

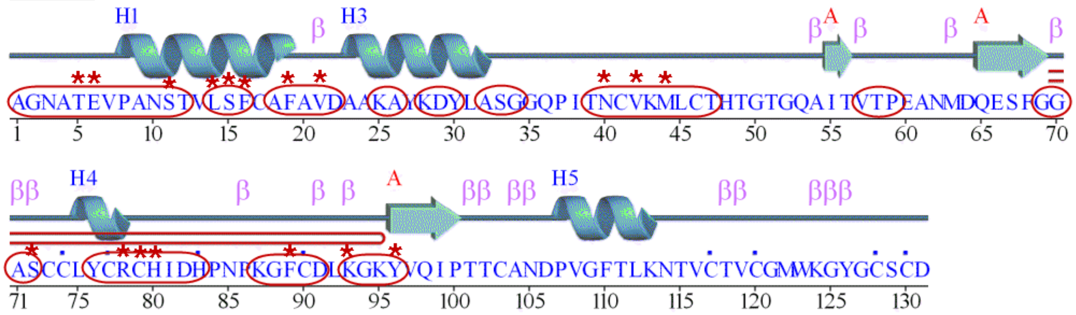
AGNATEVPANSTVLSFCAFAVDAAKAYKDYLASGGQPITNCVKMLCTHTGTGQAITVTPEANMDQESFGG
ASCCLYCRCHIDHPNPKGFCDLKGKYVQIPTTCANDPVGFTLKNTVCTVCGMWKGYGCSCD

nsp14

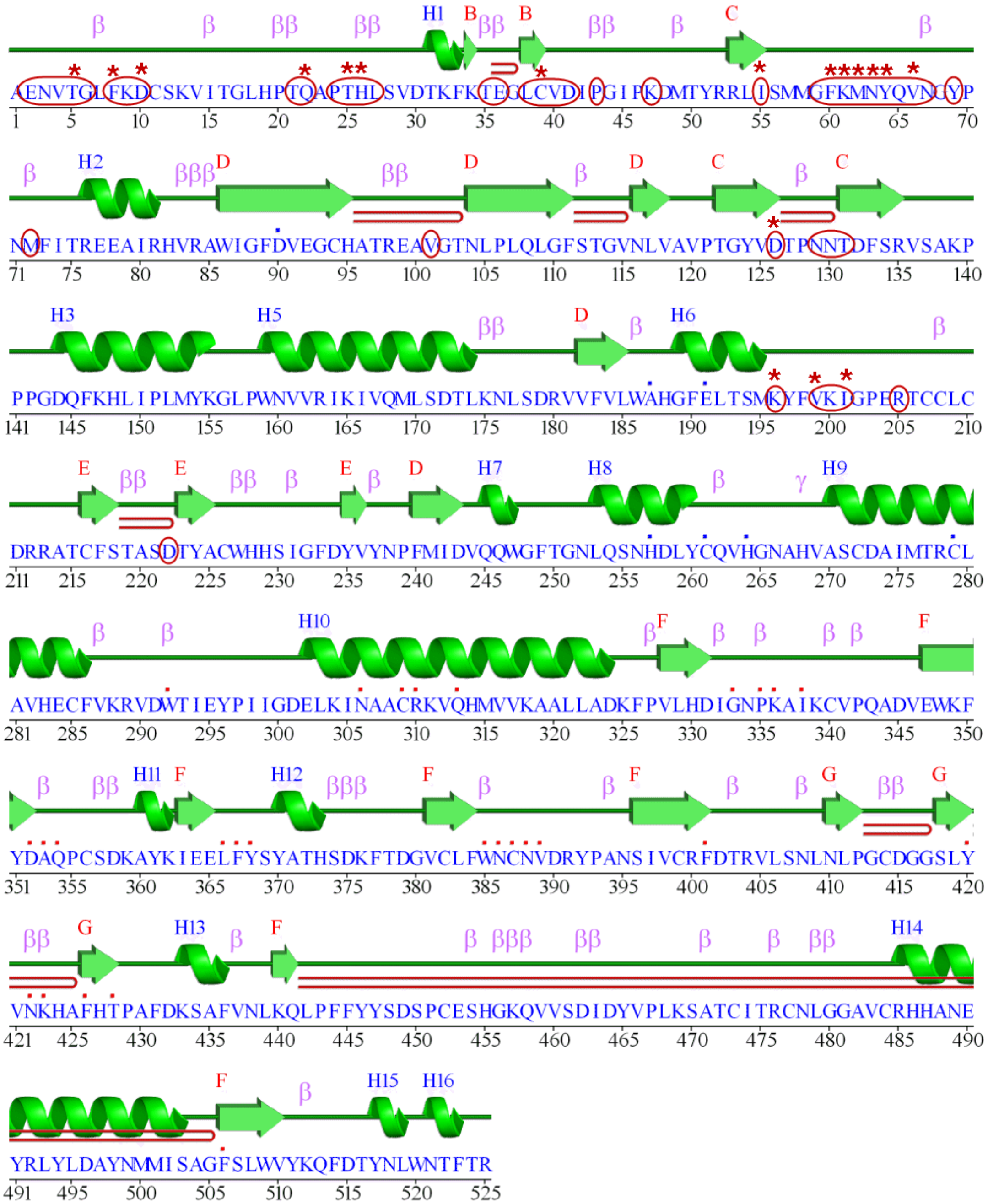
AENVTGLFKDCSKVITGLHPTQAPTHLSVDTKFKTEGLCVDIPGIPKDMTYRRLISMMGFKMNYQVNGYP
NMFITREEAIRHVRAWIGFDVEGCHATREAVGTNLPLQLGFSTGVNLVAVPTGYVDTPNNTDFSRVSAKP
PPGDQFKHLIPLMYKGLPWNVVRIKIVQMLSDTLKNLSDRVVFVLWAHGFELTSMKYFVKIGPERTCCLC
DRRATCFSTASDTYACWHHSIGFDYVYNPFMIDVQQWGFTGNLQSNHDLYCQVHGNAHVASCDAIMTRCL
AVHECFVKRVDWTIEYPIIGDELKINAACRKVQHMVVKAALLADKFPVLHDIGNPKAIKCVPQADVEWKF
YDAQPCSDKAYKIEELFYSYATHSDKFTDGVCLFWNCNVDRYPANSIVCRFDTRVLSNLNLPGCDGGSLY
VNKHAFHTPAFDKSAFVNLKQLPFFYYSDSPCESHGKQVVSDIDYVPLKSATCITRCNLGGAVCRHHANE
YRLYLDAYNMMISAGFSLWVYKQFDTYNLWNTFTR

Figure S5: Secondary structure plot of the SARS-CoV-2 nsp14-nsp10 complex. The red boxes denote the predicted interfacial residues. The predicted hotspot residues are indicated with red asterisks.

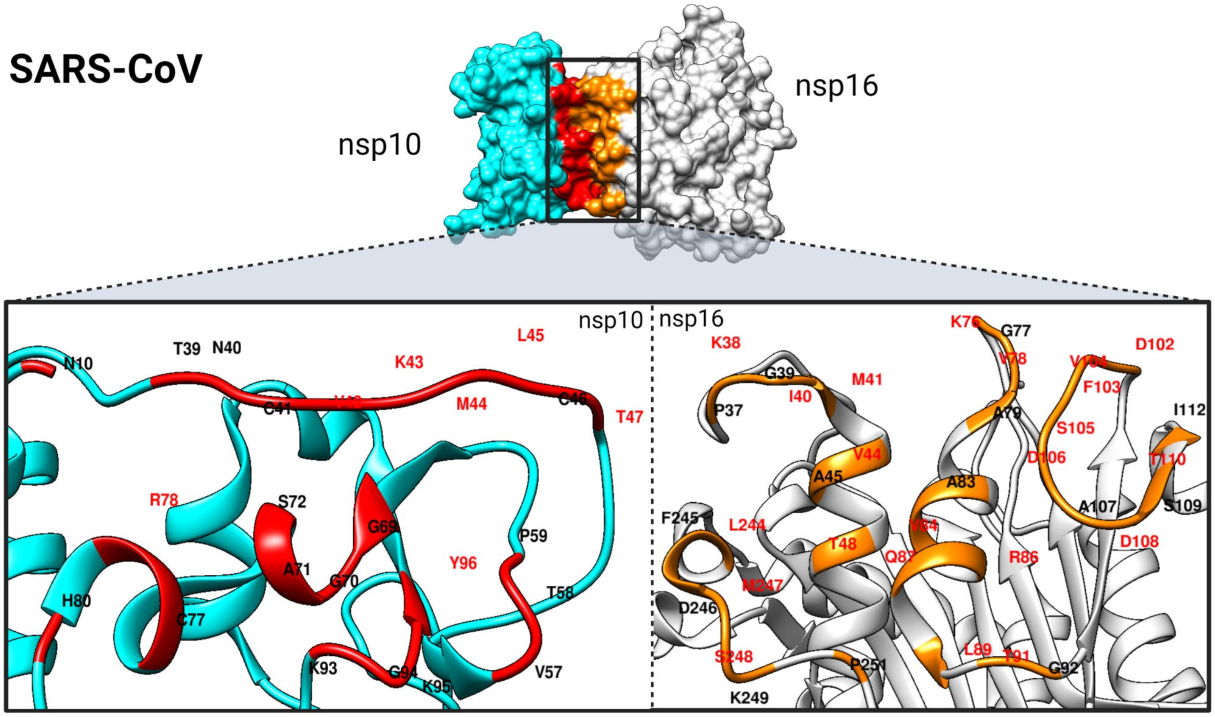

Figure S6: Analysis of the SARS-CoV nsp16-nsp10 protein-protein interface and hotspot residues. The interfaces and hotspots were mapped to the structures at the protein-protein interface of nsp16 (gray) and nsp10 (cyan), which are colored orange and red, respectively. The hotspot residues are shown in red labels.

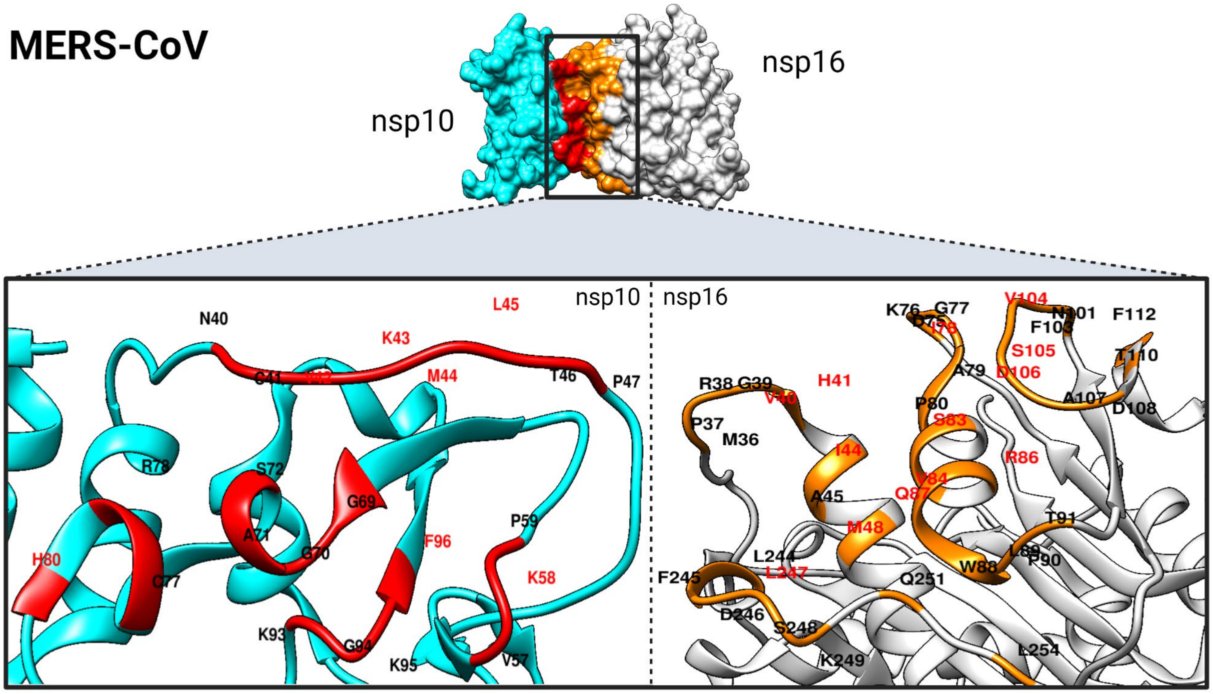

Figure S7: Analysis of the MERS-CoV nsp16-nsp10 protein-protein interface and hotspot residues. The interfaces and hotspots were mapped to the structures at the protein-protein interface of nsp16 (gray) and nsp10 (cyan), which are colored orange and red, respectively. The hotspot residues are shown in red labels.

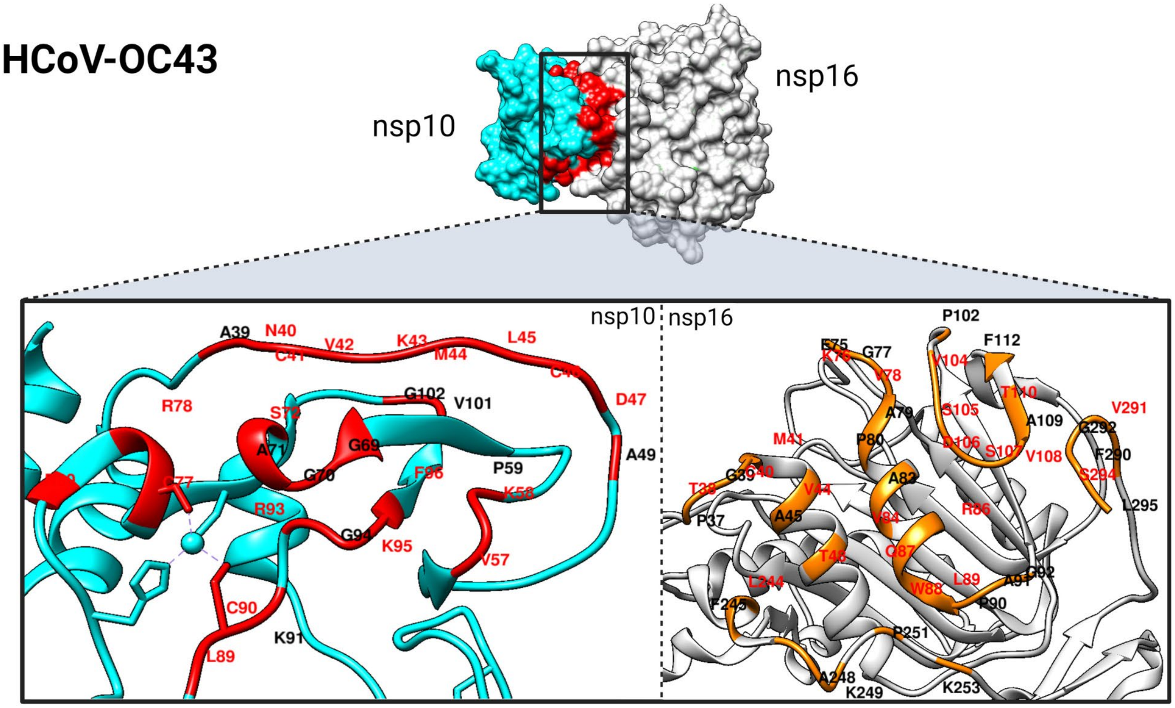

Figure S8: Analysis of the HCoV-OC43 nsp16-nsp10 protein-protein interface and hotspot residues. The interfaces and hotspots were mapped to the structures at the protein-protein interface of nsp16 (gray) and

nsp10 (cyan), which are colored orange and red, respectively. The hotspot residues are shown in red labels.

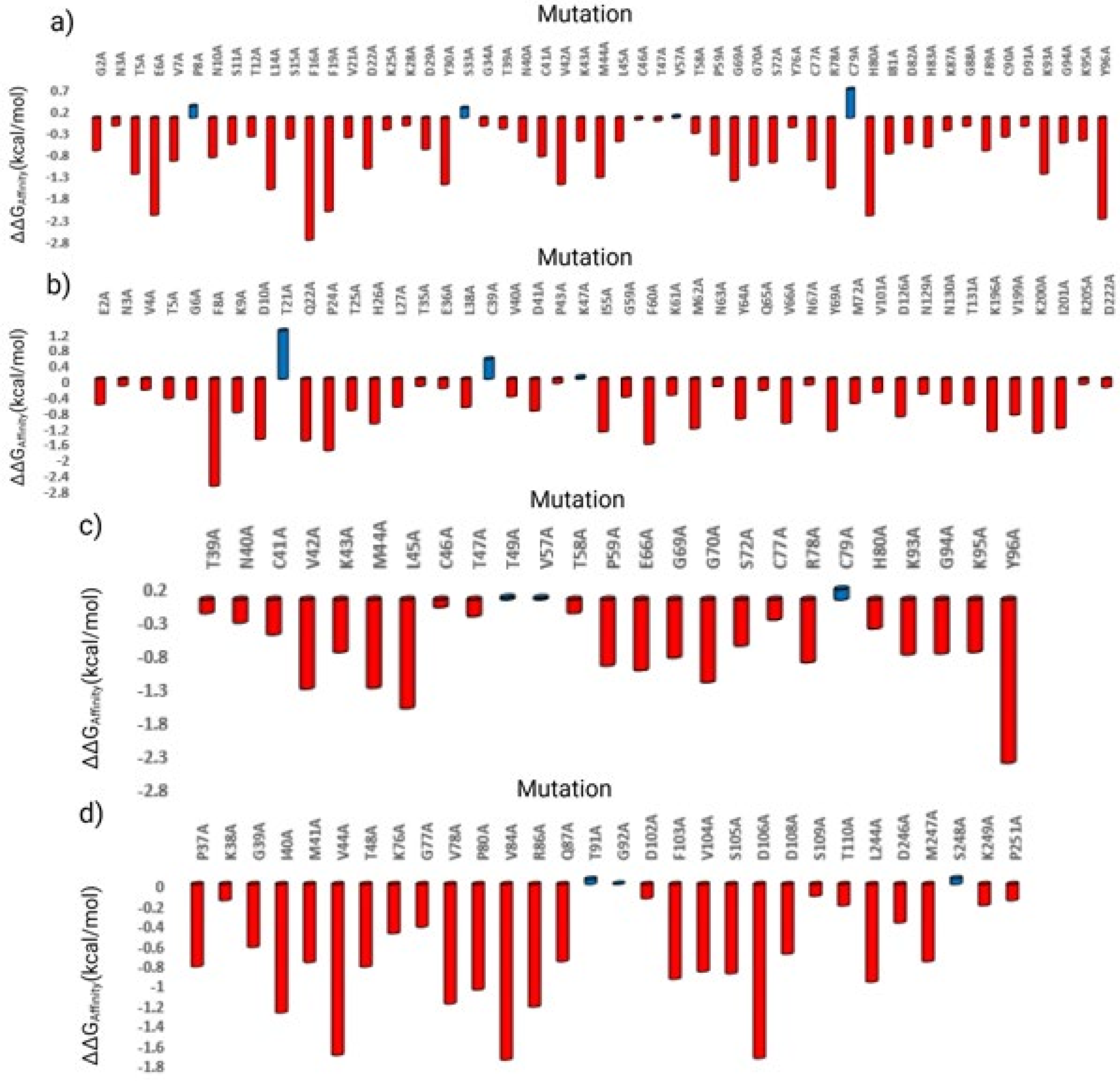

Figure S9: The results of computational alanine scanning (CAS) calculations for the predicted key interacting residues of the SARS-CoV-2 nsp16-nsp10 and nsp14-nsp10 complexes. (a) the bar chart summarized the changes in binding affinity upon mutation of nsp10 key residues to alanine in the nsp14-nsp10 complex, (b) the bar chart summarized the changes in binding affinity upon mutation of nsp14 key residues to alanine in the nsp14-nsp10 complex, (c) the bar chart summarized the changes in binding affinity upon mutation of nsp10 key residues to alanine in the nsp16-nsp10 complex, and (d) the bar chart

summarized the changes in binding affinity upon mutation of nsp16 key residues to alanine in the nsp16-nsp10 complex. The red and blue bars show the decreasing (negative $\Delta\Delta G_{Affinity}$) and increasing (positive $\Delta\Delta G_{Affinity}$) impacts on PPIs, respectively.

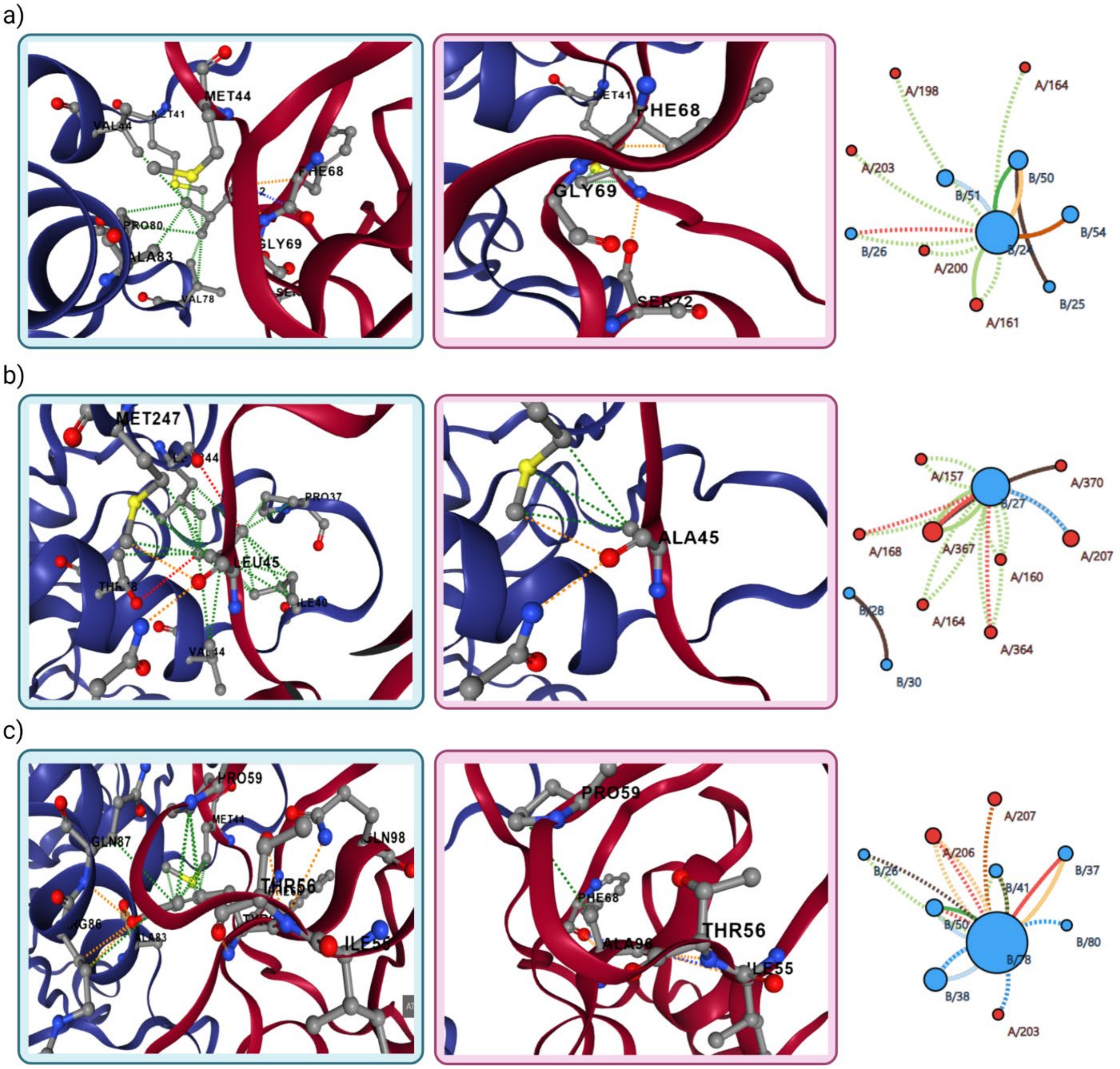

Figure S10: Interactions between wild-type (V42, L45, and Y96 of nsp10) and mutant (alanine) residues and their nearby residues in the nsp16-nsp10 complex. The interactions between the wild-type and mutant residues and their nearby residues on the left, and the inter-residue interactions of the wild-type and mutant on the right for: (a) V42 of nsp10 mutated to alanine in the nsp16-nsp10 complex, (b) L45 of nsp10 mutated to alanine in the nsp16-nsp10 complex, and (c) Y96 of nsp10 mutated to alanine in the nsp16-nsp10 complex. The interactions of wild type and mutant residues are represented by blue and

purple boxes, respectively. The nsp16 protein is chain A and is represented in cartoon and in blue. The nsp10 protein is chain B depicted in cartoon and red. The dashed lines and straight lines represent the wild type and mutant inter-residues interactions, respectively. The aromatic, carbonyl, hydrophobic, hydrogen bond, ionic, VDW, ring-ring, and polar-Hbond interactions are colored in light green, dark blue, dark green, red, yellow, light blue, black, and orange, respectively.

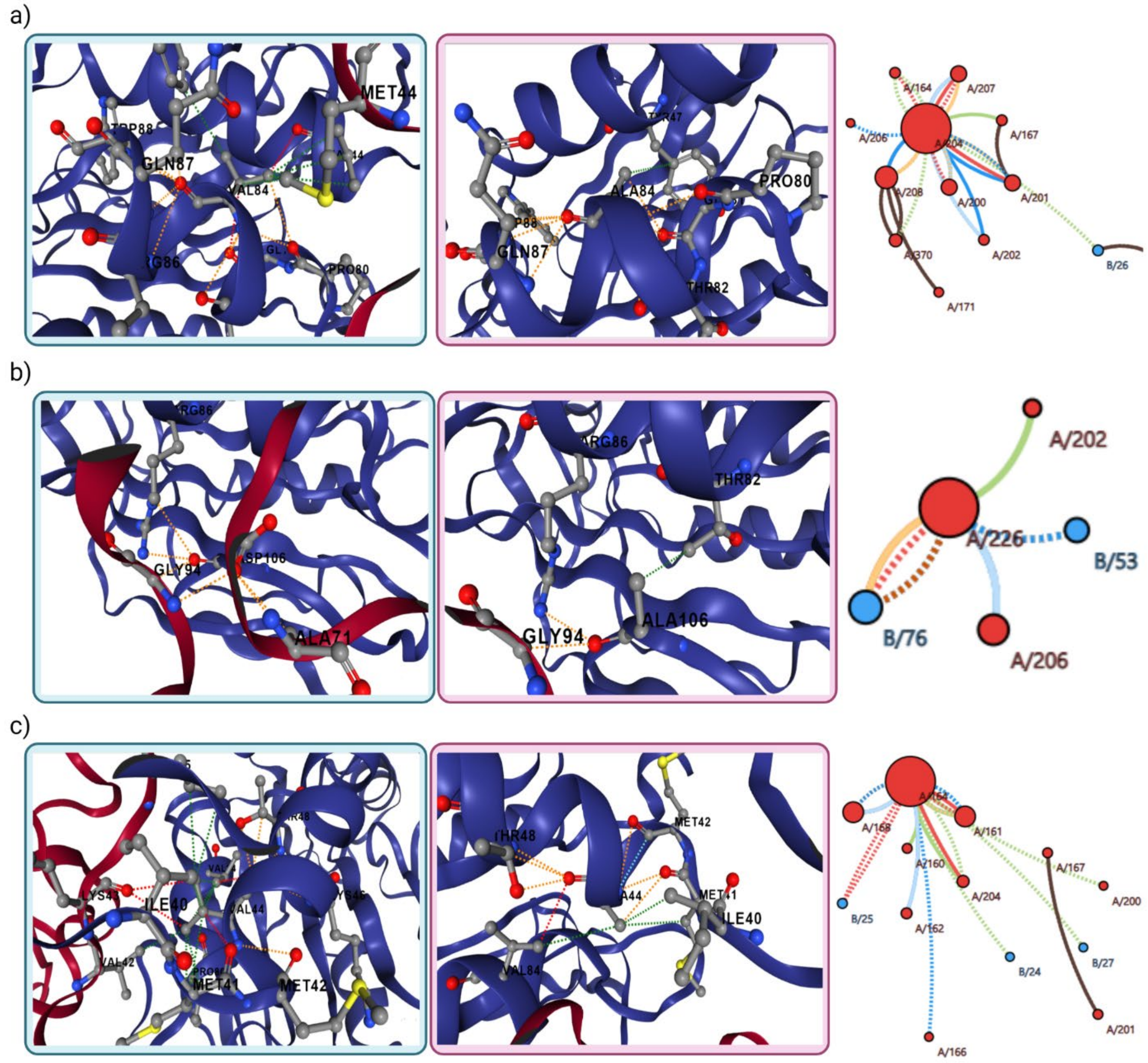

Figure S11: Interactions between wild-type (V84, D106, and V44 of nsp16) and mutant (alanine) residues and their nearby residues in the nsp16-nsp10 complex. The interactions between the wild-type and mutant residues and their nearby residues on the left, and the inter-residue interactions of the wild-type and mutant on the right for: (a) V84 of nsp16 mutated to alanine in the nsp16-nsp10 complex, (b) D106 of nsp16 mutated to alanine in the nsp16-nsp10 complex, (c) V44 of nsp16 mutated to alanine in the nsp16-nsp10 complex. The interactions of wild type and mutant residues are represented by blue and

purple boxes, respectively. The nsp16 protein is chain A and is represented in cartoon and in blue. The nsp10 protein is chain B depicted in cartoon and red. The dashed lines and straight lines represent the wild type and mutant inter-residues interactions, respectively. The aromatic, carbonyl, hydrophobic, hydrogen bond, ionic, VDW, ring-ring, and polar-Hbond interactions are colored in light green, dark blue, dark green, red, yellow, light blue, black, and orange, respectively.

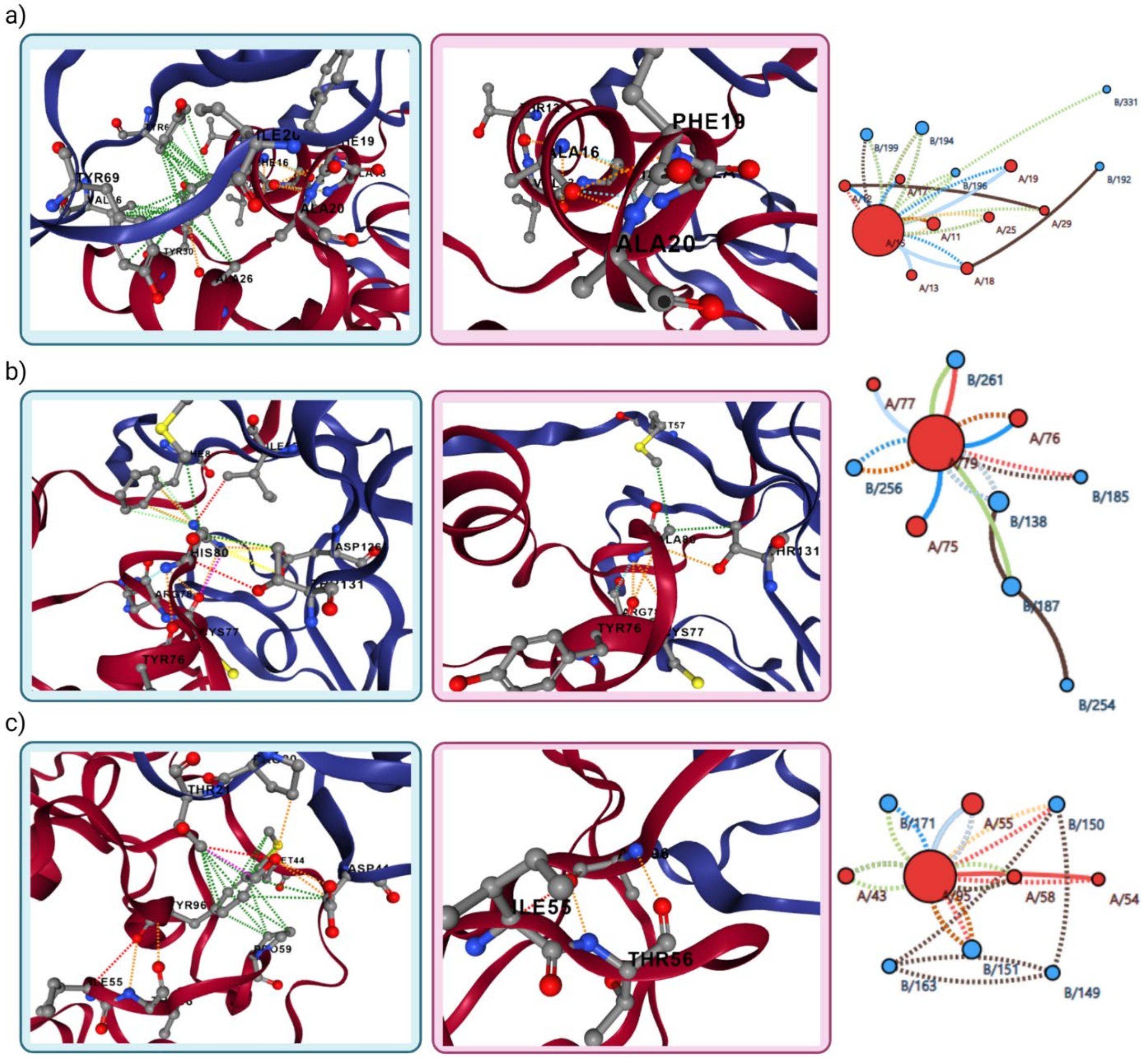

Figure S12: Interactions between wild-type (F16, H80, and Y96 of nsp10) and mutant (alanine) residues and their nearby residues in the nsp14-nsp10 complex. The interactions between the wild-type and mutant residues and their nearby residues on the left, and the inter-residue interactions of the wild-type and mutant on the right for: (a) F16 of nsp10 mutated to alanine in the nsp14-nsp10 complex, (b) H80 of nsp10 mutated to alanine in the nsp14-nsp10 complex, and (c) Y96 of nsp10 mutated to alanine in the nsp14-nsp10 complex. The interactions of wild type and mutant residues are represented by blue and

purple boxes, respectively. The nsp14 protein is chain B and is represented in cartoon and blue. The nsp10 protein is chain A depicted in cartoon and red. The dashed lines and straight lines represent the wild type and mutant inter-residues interactions, respectively. The aromatic, carbonyl, hydrophobic, hydrogen bond, ionic, VDW, ring-ring, and polar-Hbond interactions are colored in light green, dark blue, dark green, red, yellow, light blue, black, and orange, respectively.

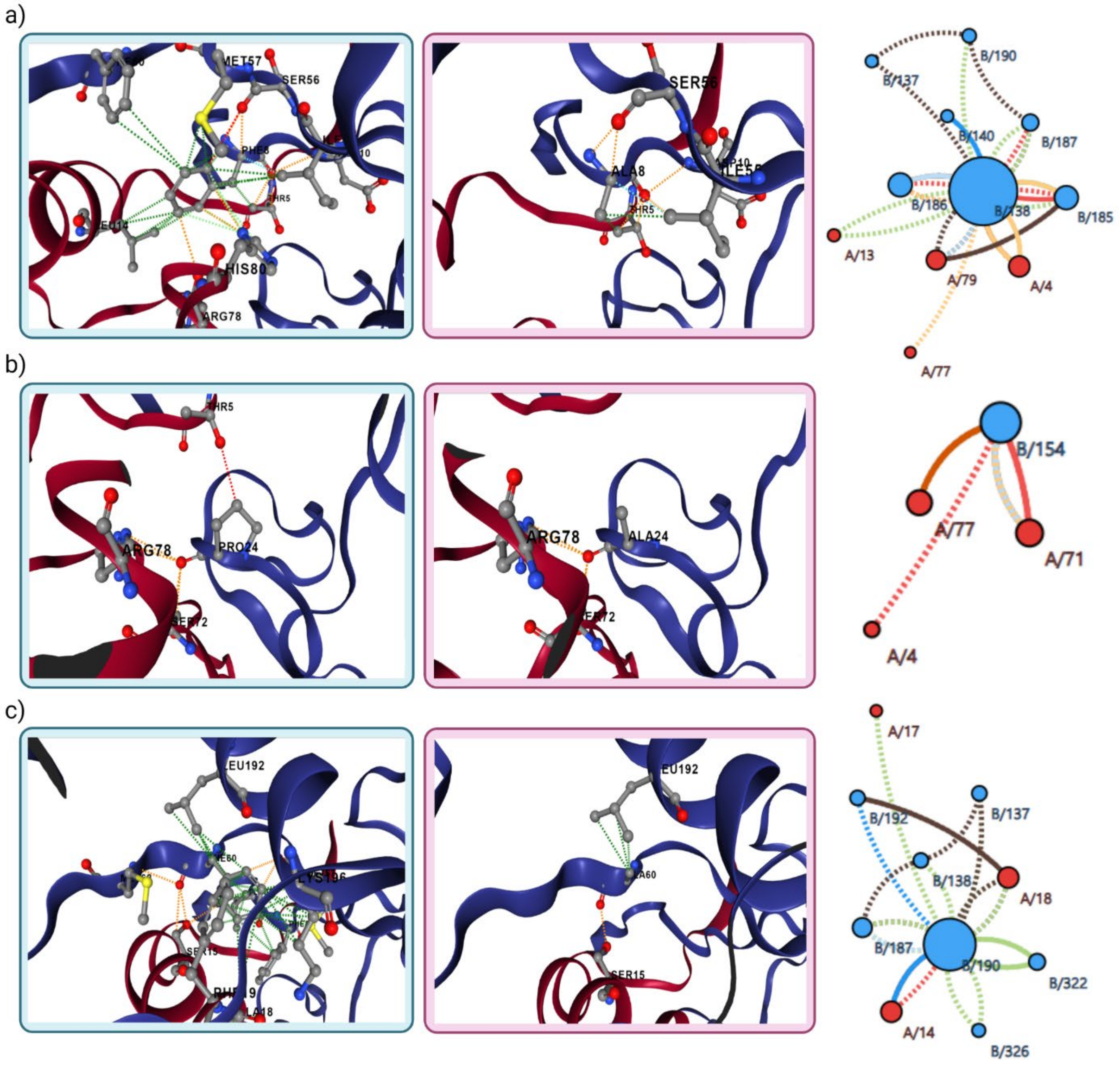

Figure S13: Interactions between wild-type (F8, P24, and F60 of nsp14) and mutant (alanine) residues and their nearby residues in the nsp14-nsp10 complex. The interactions between the wild-type and mutant residues and their nearby residues on the left, and the inter-residue interactions of the wild-type and mutant on the right for: (a) F8 of nsp14 mutated to alanine in the nsp14-nsp10 complex, (b) P24 of nsp14 mutated to alanine in the nsp14-nsp10 complex, and (c) F60 of nsp14 mutated to alanine in the nsp14-nsp10 complex. The interactions of wild type and mutant residues are represented by blue and purple

boxes, respectively. The nsp14 protein is chain B and is represented in cartoon and blue. The nsp10 protein is chain A depicted in cartoon and red. The dashed lines and straight lines represent the wild type and mutant inter-residues interactions, respectively. The aromatic, carbonyl, hydrophobic, hydrogen bond, ionic, VDW, ring-ring, and polar-Hbond interactions are colored in light green, dark blue, dark green, red, yellow, light blue, black, and orange, respectively.

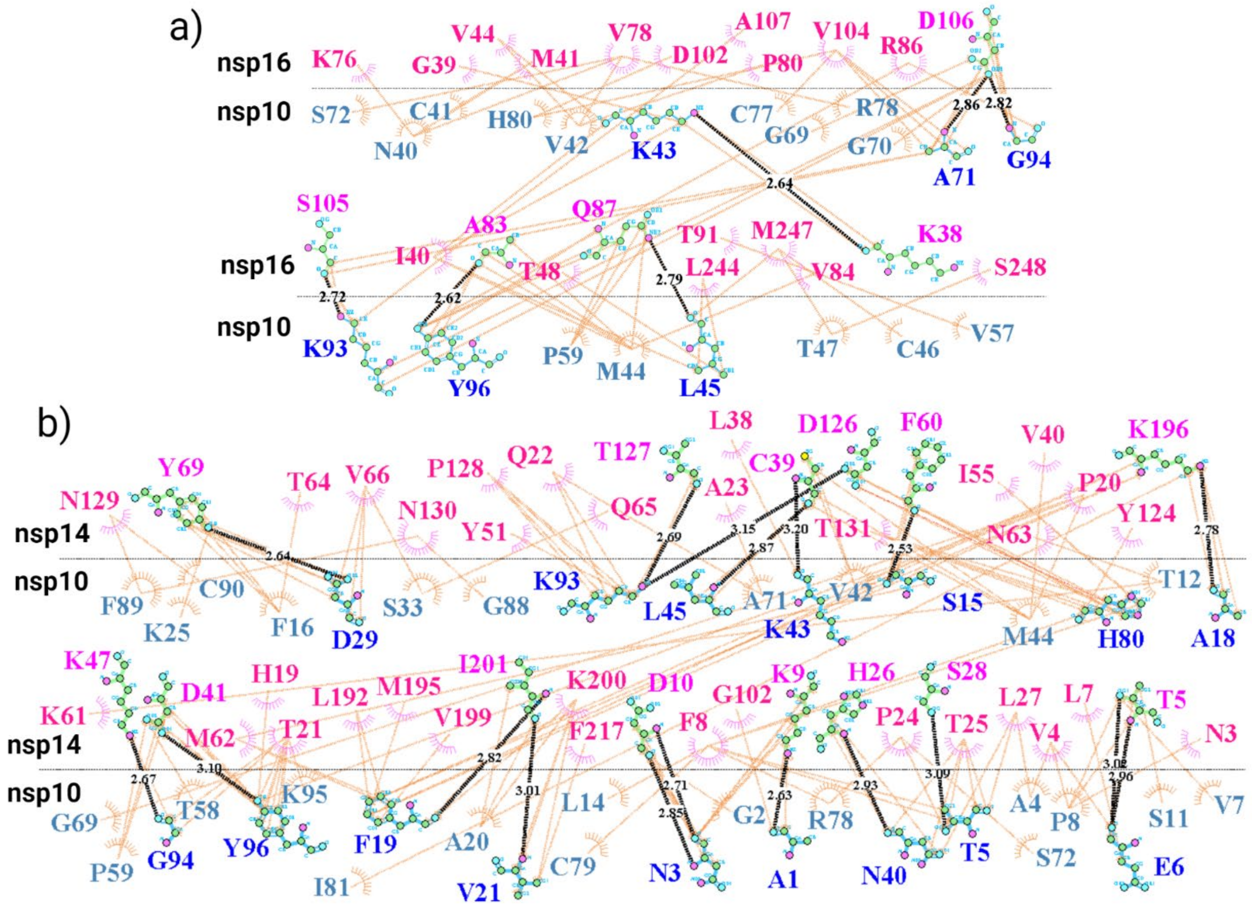

Figure S14: Two-dimensional residue interaction maps for the SARS-CoV-2 nsp16-nsp10 and nsp14-nsp10 complexes. (a) Two-dimensional residue interaction map for the nsp16-nsp10 complex. (b) Two-dimensional residue interaction map for the nsp14-nsp10 complex. The horizontal dashed line represents the complex interface. The black dashed lines and pink and brown arcs with spokes represent the hydrogen bonds and hydrophobic contacts, respectively.

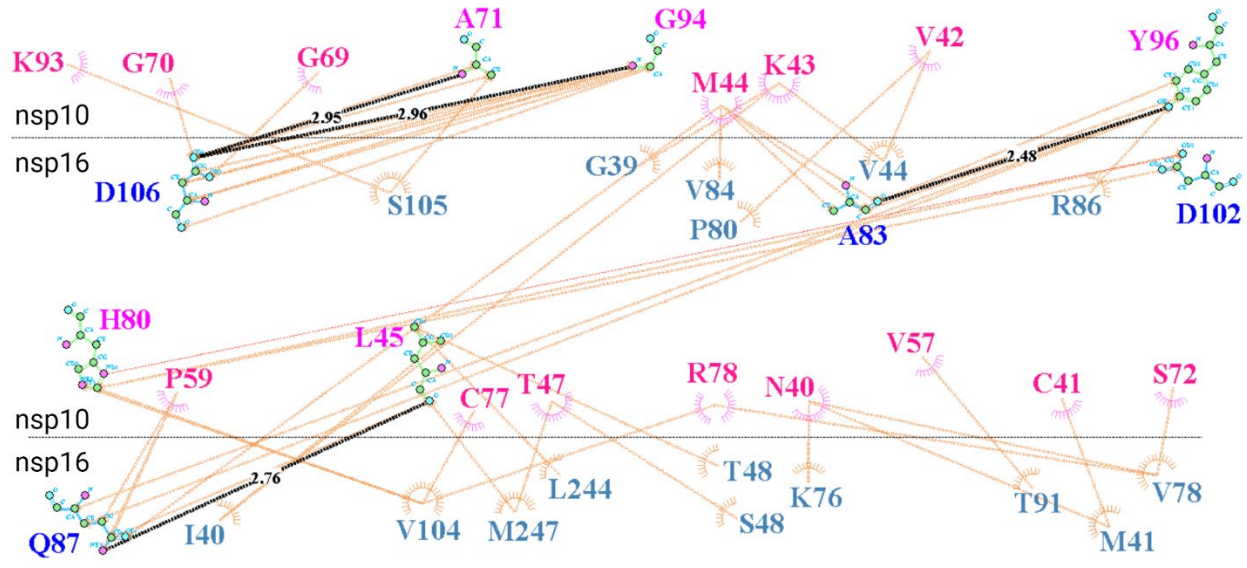

Figure S15: Two-dimensional residue interaction map for the SARS-CoV nsp16-nsp10 complex. The horizontal dashed line represents the complex interface. The black dashed lines and pink and brown arcs with spokes represent the hydrogen bonds and the hydrophobic contacts, respectively.

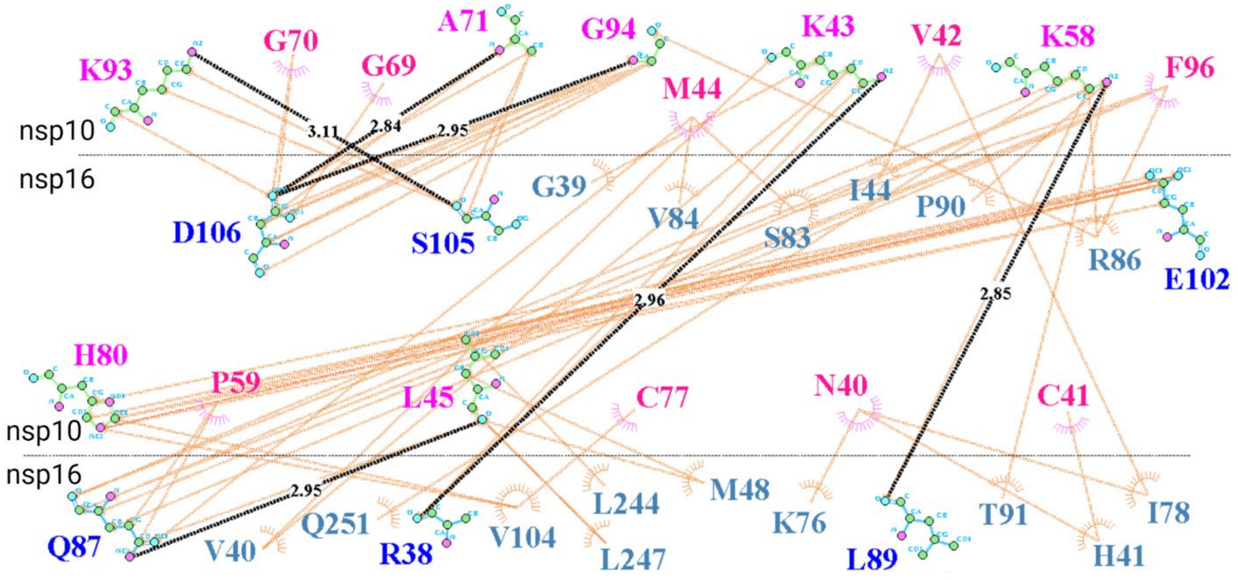

Figure S16: Two-dimensional residue interaction map for the MERS-CoV nsp16-nsp10 complex. The horizontal dashed line represents the complex interface. The black dashed lines and pink and brown arcs with spokes represent the hydrogen bonds and the hydrophobic contacts, respectively.

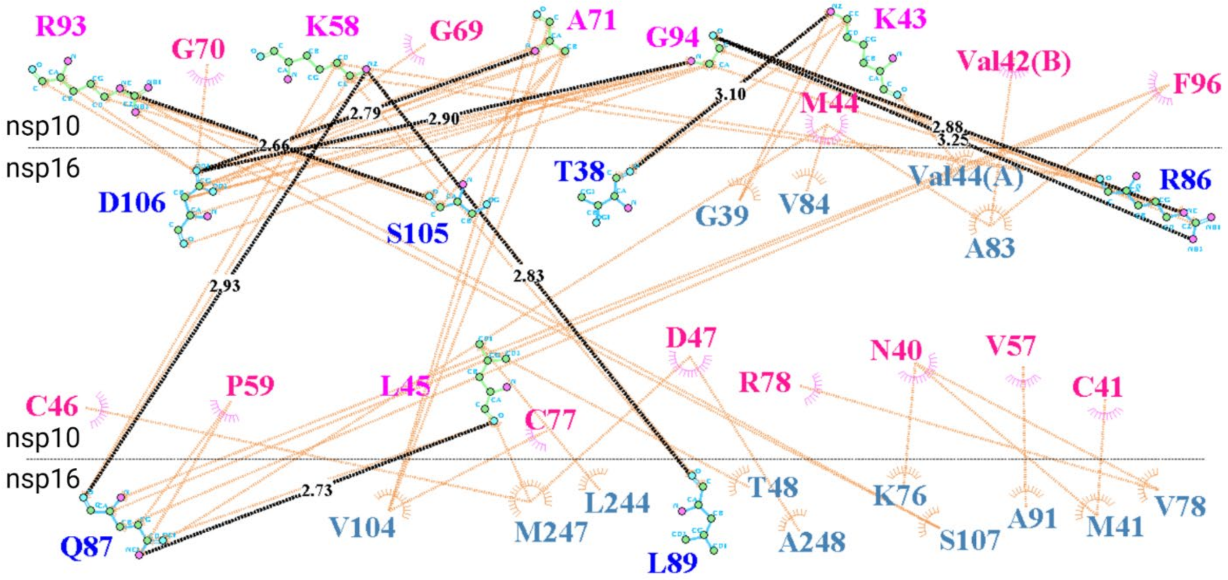

Figure S17: Two-dimensional residue interaction map for the HCoV-OC43 nsp16-nsp10 complex. The horizontal dashed line represents the complex interface. The black dashed lines and pink and brown arcs with spokes represent the hydrogen bonds and the hydrophobic contacts, respectively.

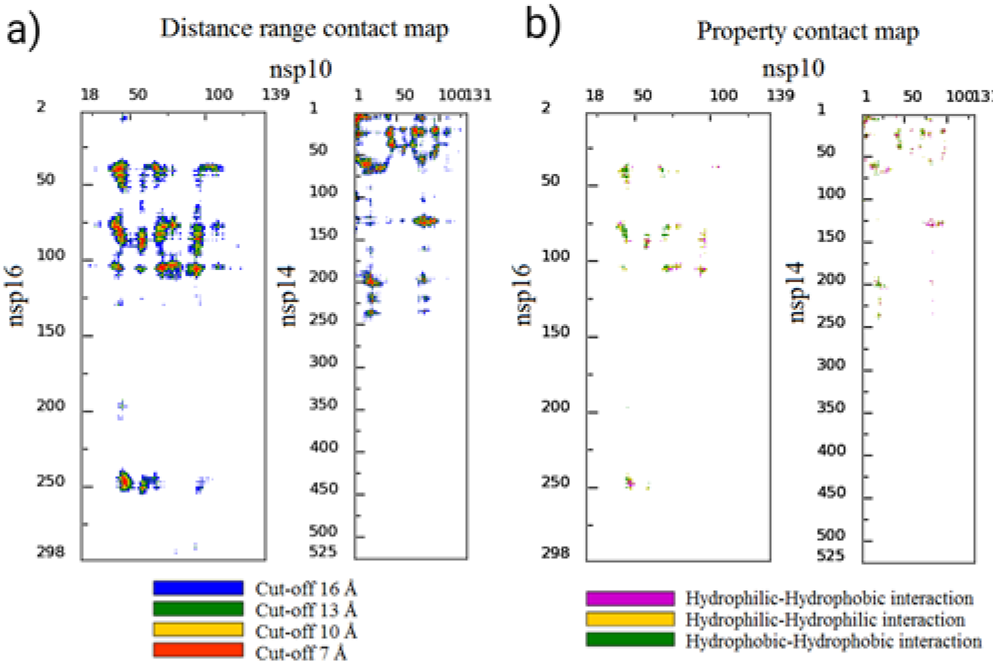

Figure S18: Distance range and property contact maps for the SARS-CoV-2 nsp16-nsp10 and nsp14-nsp10 complexes. (a) Distance range contact maps, and (b) property contact maps.

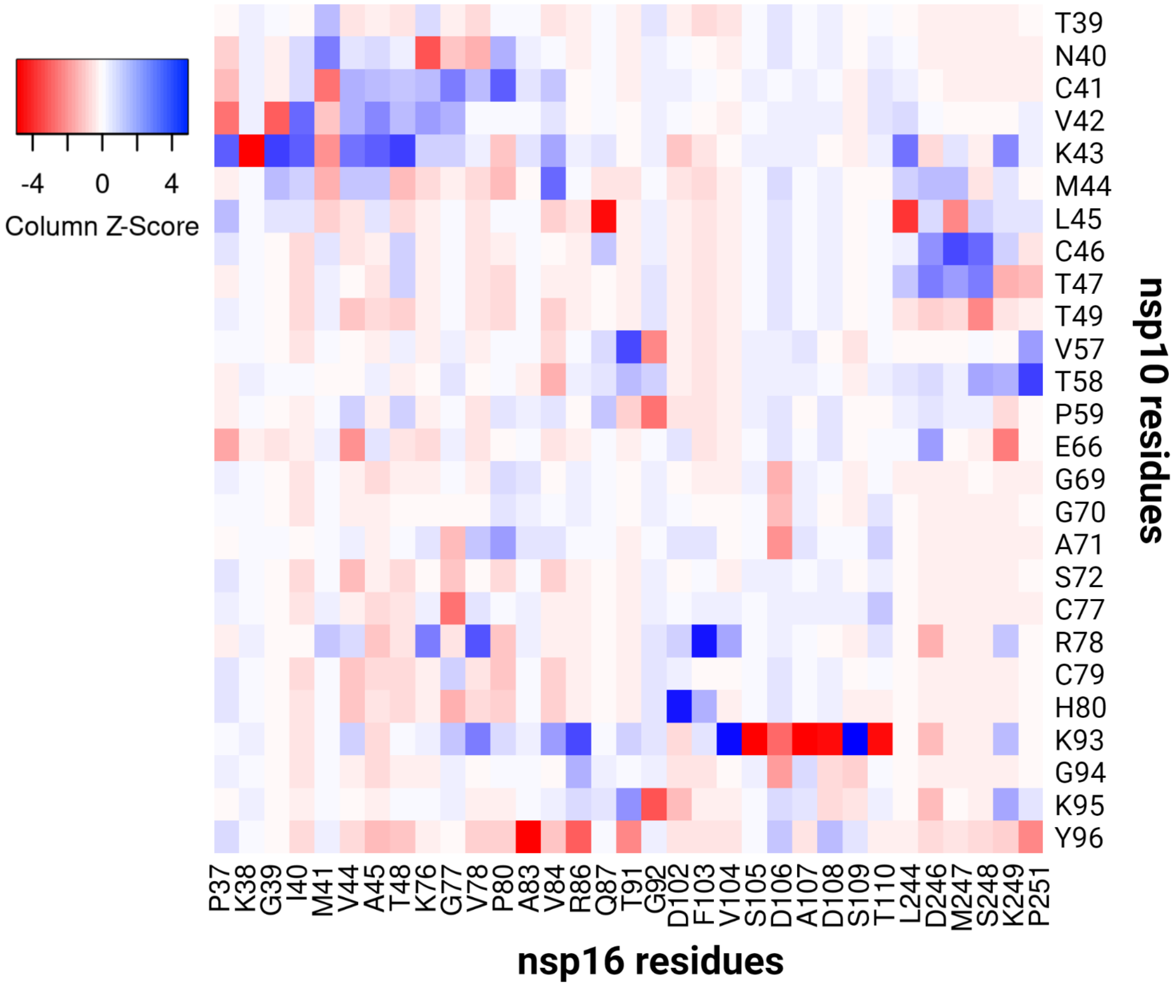

Figure S19: The heat map for residue-residue pairwise interaction energies (IEs) between the predicted key interacting residues of nsp10 and nsp16 in the SAR-CoV-2 nsp16-nsp10 complex. The negative (with the stabilizing roles in PPIs) and positive (with the destabilizing roles in PPIs) pairwise IEs are colored in red and blue, respectively.

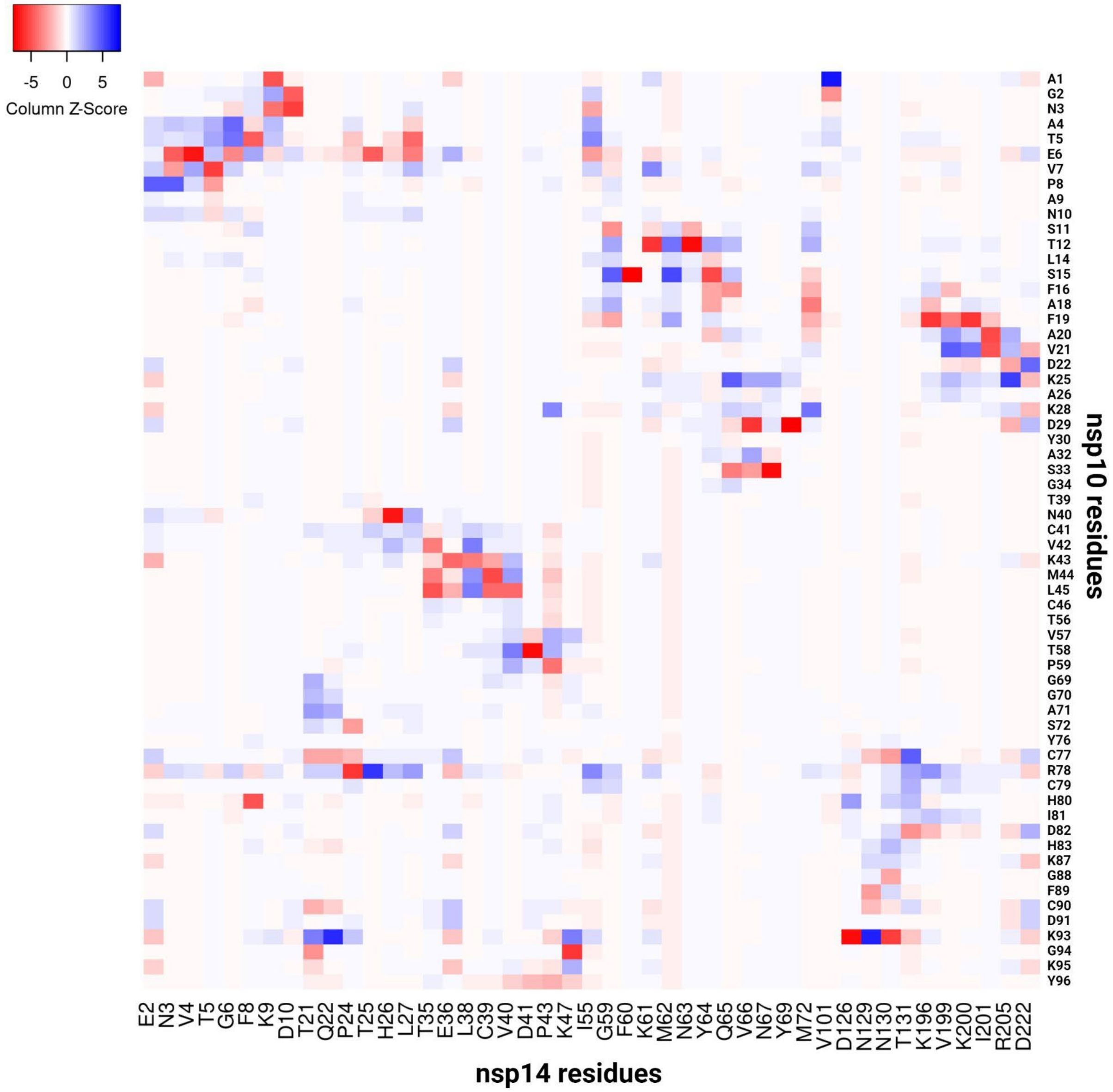

Figure S20: The heat map for residue-residue pairwise interaction energies (IEs) between the predicted key interacting residues of nsp10 and nsp14 in the SARS-CoV-2 nsp14-nsp10 complex. The negative (with the stabilizing roles in PPIs) and positive (with the destabilizing roles in PPIs) pairwise IEs are colored in red and blue, respectively.

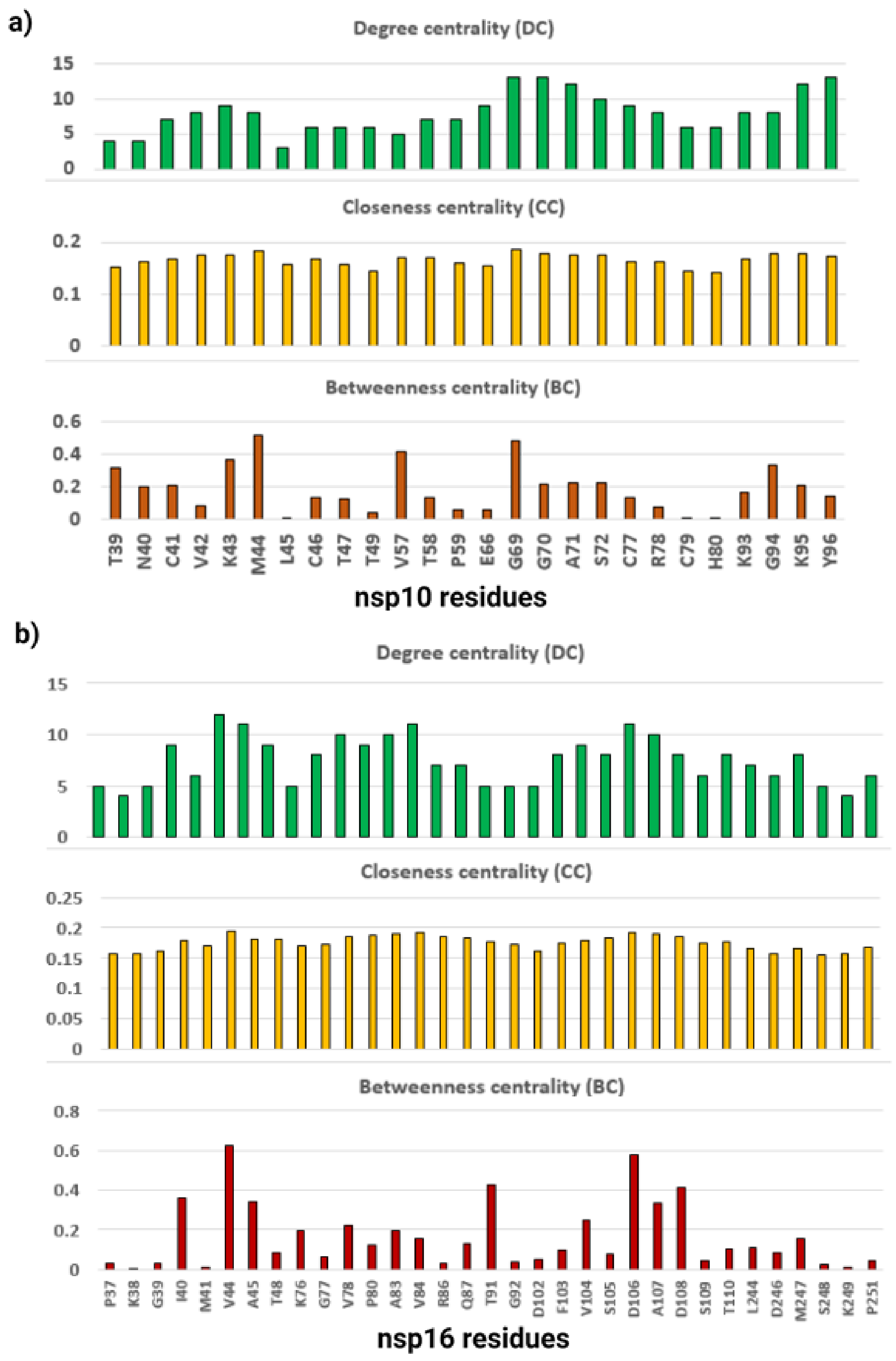

Figure S21: Node centrality parameters in the network, including degree centrality (DC), betweenness centrality (BC), and closeness centrality (CC) of the predicted key interacting residues of the SARS-CoV-

2 nsp16-nsp10 complex. (a) Node centrality parameters for the predicted key interacting residues of nsp10, and (b) the node centrality parameters for the predicted key interacting residues of nsp16.

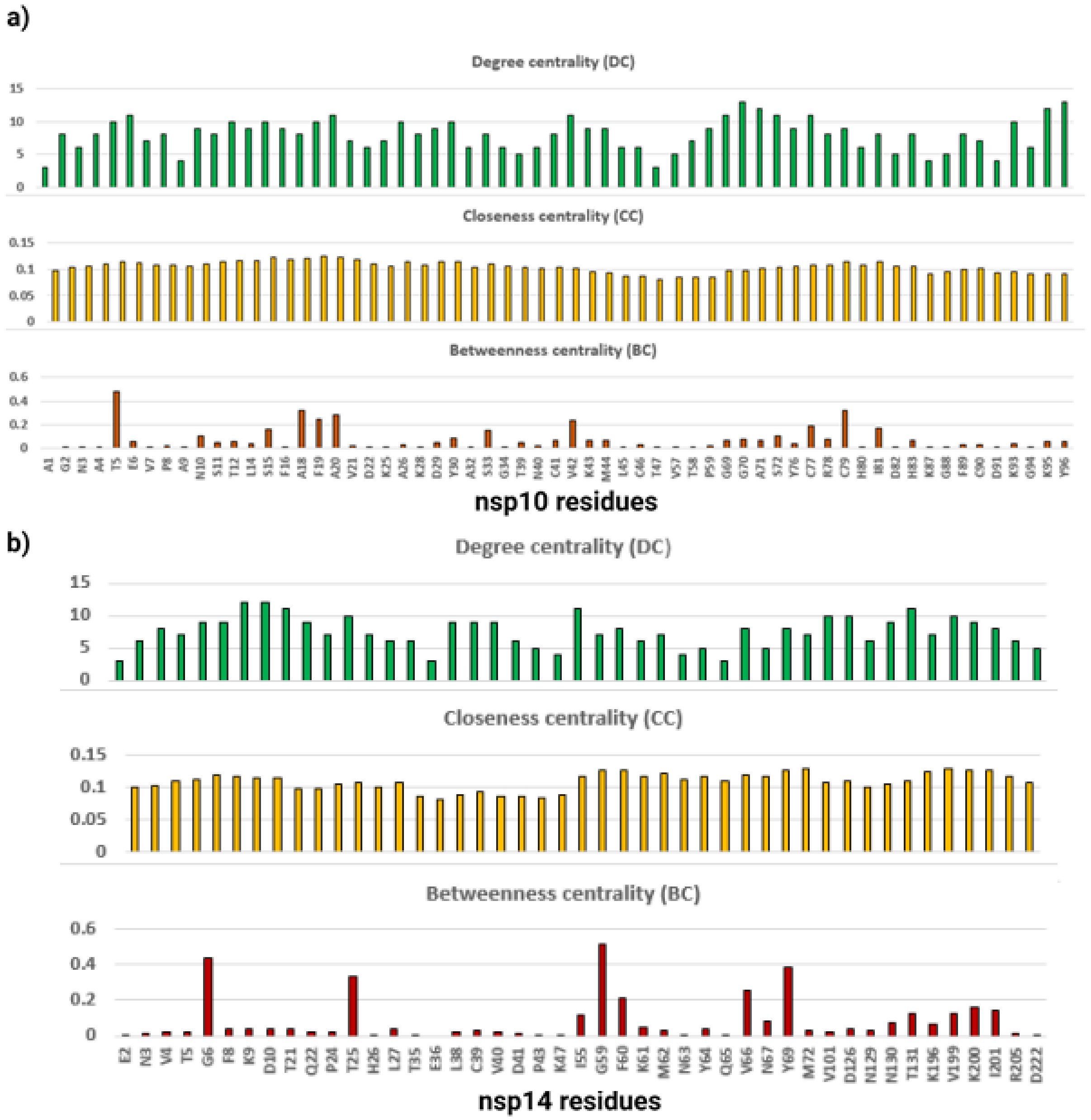

Figure S22: Node centrality parameters in the network, including degree centrality (DC), betweenness centrality (BC), and closeness centrality (CC) of the predicted key interacting residues of the SARS-CoV-2 nsp14-nsp10 complex. (a) Node centrality parameters for the predicted key interacting residues of nsp10, and (b) the node centrality parameters for the predicted key interacting residues of nsp14.

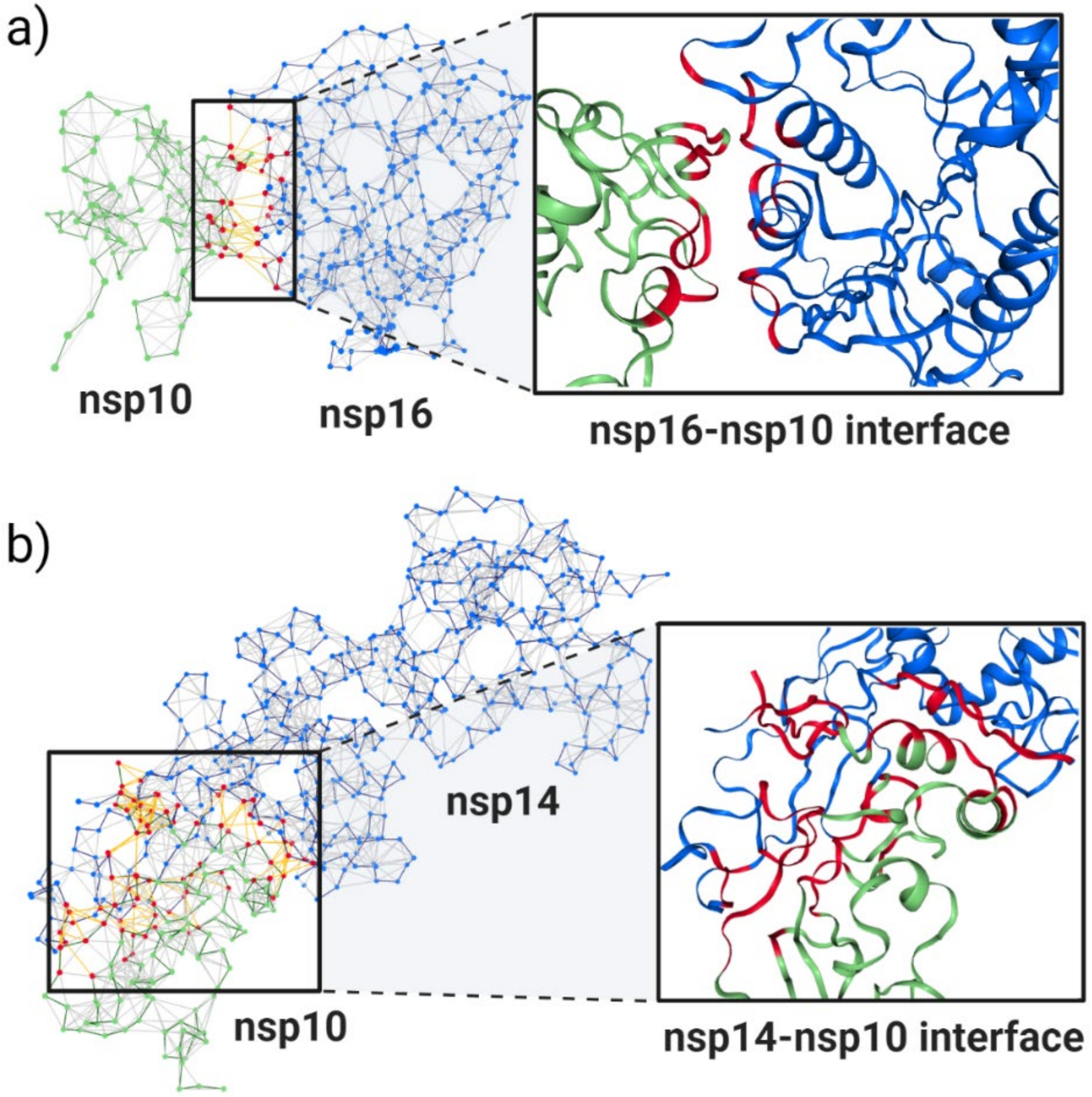

Figure S23: 3D networks of residue–residue interactions for the SARS-CoV-2 nsp16-nsp10 and nsp14-nsp10 complexes. (a) 3D network of the nsp16-nsp10 complex, and (b) 3D network of the SARS-CoV-2 nsp14-nsp10 complex. The nodes and edges at the PPIs interface are colored in red and yellow, respectively. The protein-protein interfaces are colored in red and are shown in the box.

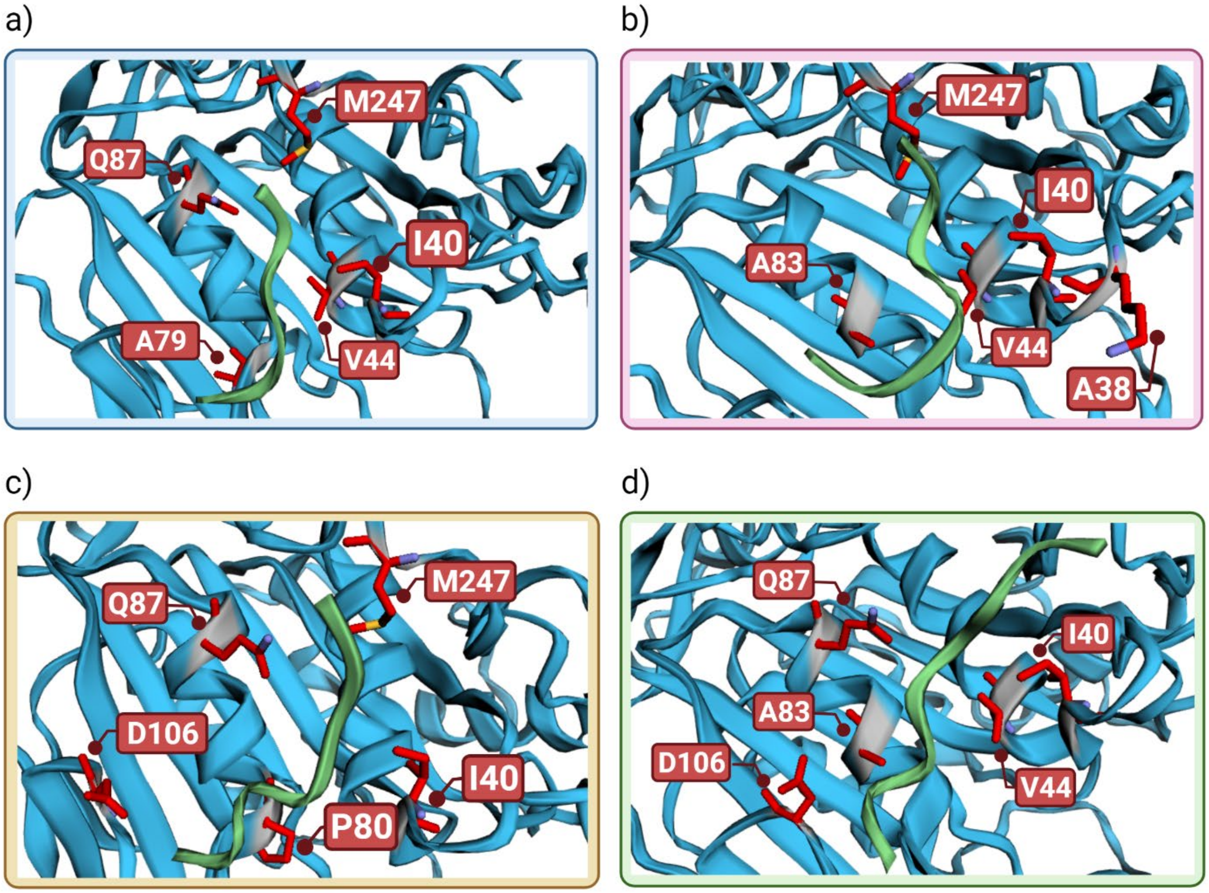

Figure S24: The top five nsp16 residues with the greatest energy contributions (the lowest energy) in the interactions of OLP-13, OLP-16, OLP-17, OLP-18 and nsp16 as target protein. (a) OLP-13 and nsp16 complex, (b) OLP-16 and nsp16 complex, (c) OLP-17 and nsp16 complex, and (d) OLP-18 and nsp16 complex. Nsp16 and the peptides are shown in blue and green, respectively.

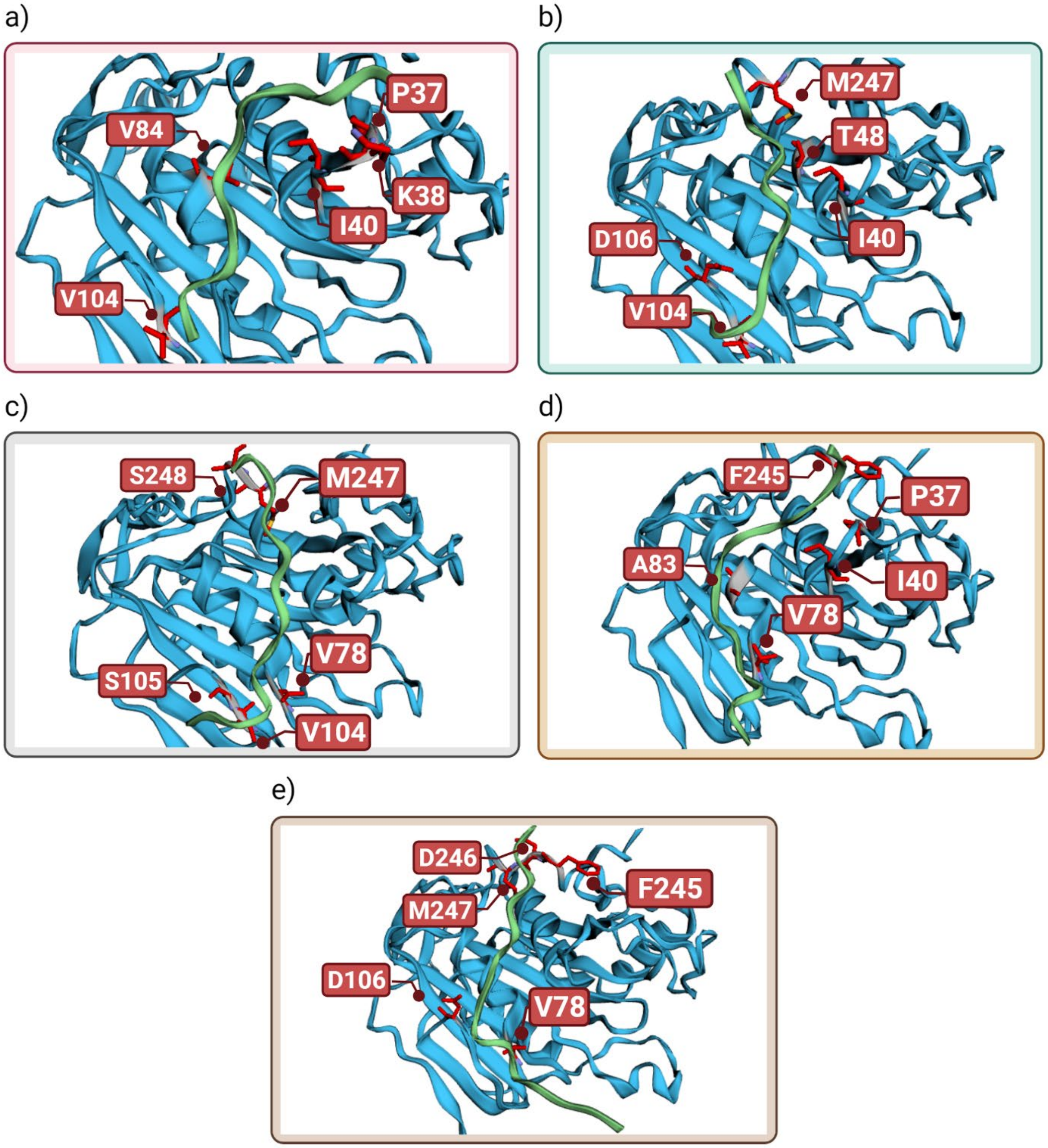

Figure S25: The top five nsp16 residues with the greatest energy contributions (the lowest energy) in the interactions of P-16-11, P-16-12, P-16-13, P-16-14, P-16-15 and nsp16 as target protein. (a) P-16-11 and nsp16 complex, (b) P-16-12 and nsp16 complex, (c) P-16-13 and nsp16 complex, (d) P-16-14 and nsp16 complex, and (e) P-16-15 and nsp16. Nsp16 and the peptides are shown in blue and green, respectively.

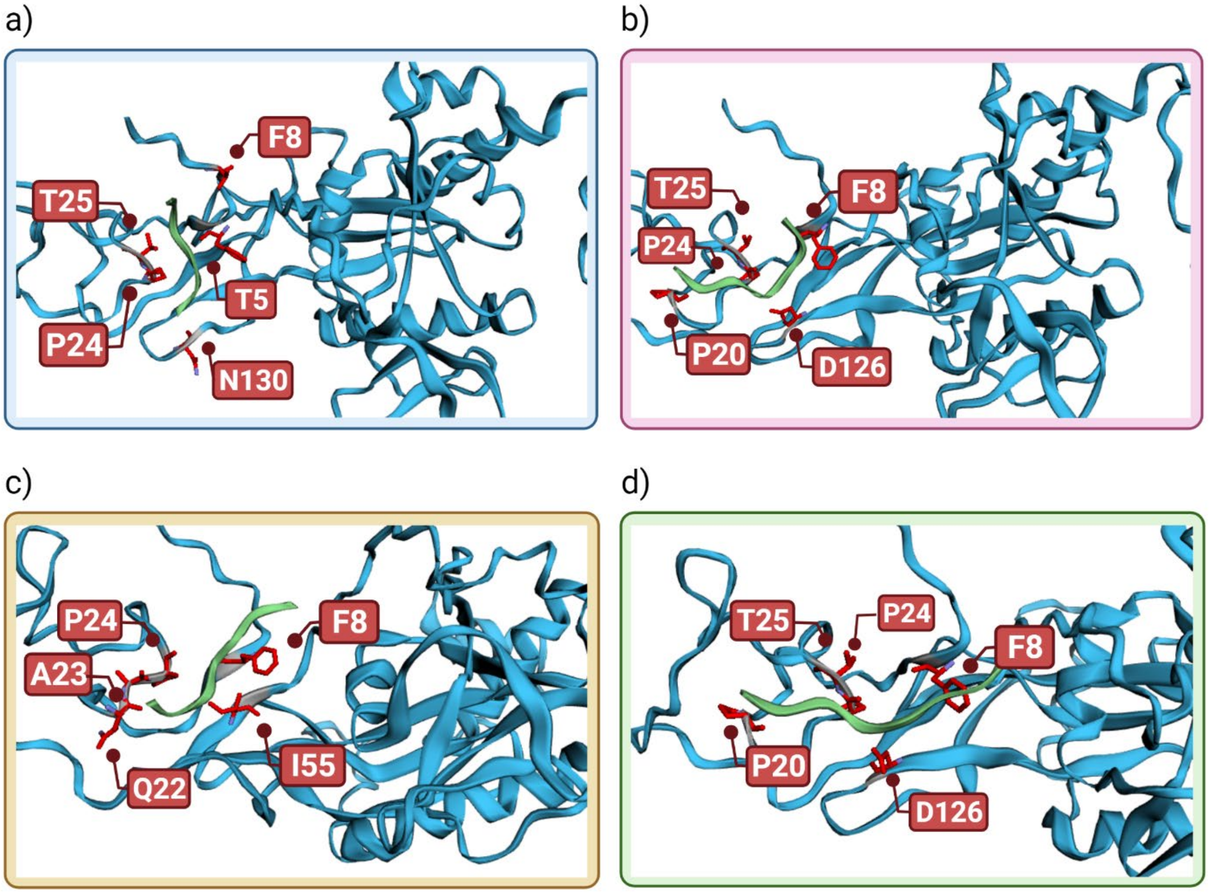

Figure S26: The top five nsp14 residues with the greatest energy contributions (the lowest energy) in the interactions of OLP-13, OLP-16, OLP-17, OLP-18 and nsp14 as target protein. (a) OLP-13 and nsp14 complex, (b) OLP-16 and nsp14 complex, (c) OLP-17 and nsp14 complex, and (d) OLP-18 and nsp14 complex. Nsp14 and the peptides are shown in blue and green, respectively.

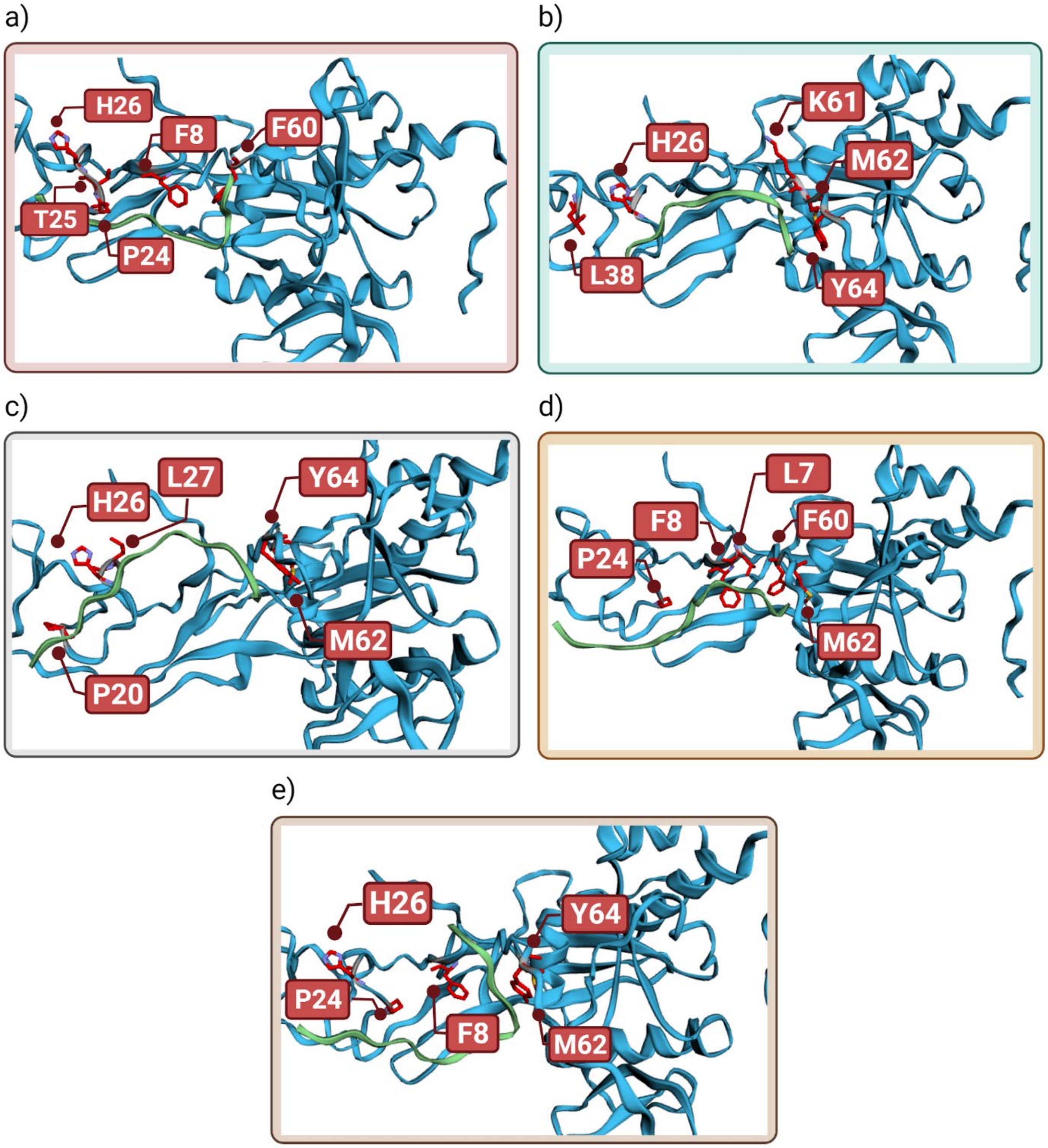

Figure S27: The top five nsp14 residues with the greatest energy contributions (the lowest energy) in the interactions of P-14-11, P-14-12, P-14-13, P-14-14, P-14-15 and nsp14 as target protein. (a) P-14-11 and nsp14 complex, (b) P-14-12 and nsp14 complex, (c) P-14-13 and nsp14 complex, (d) P-14-14 and nsp14 complex, and (e) P-14-15 and nsp14 complex. Nsp14 and the peptides are shown in blue and green, respectively.

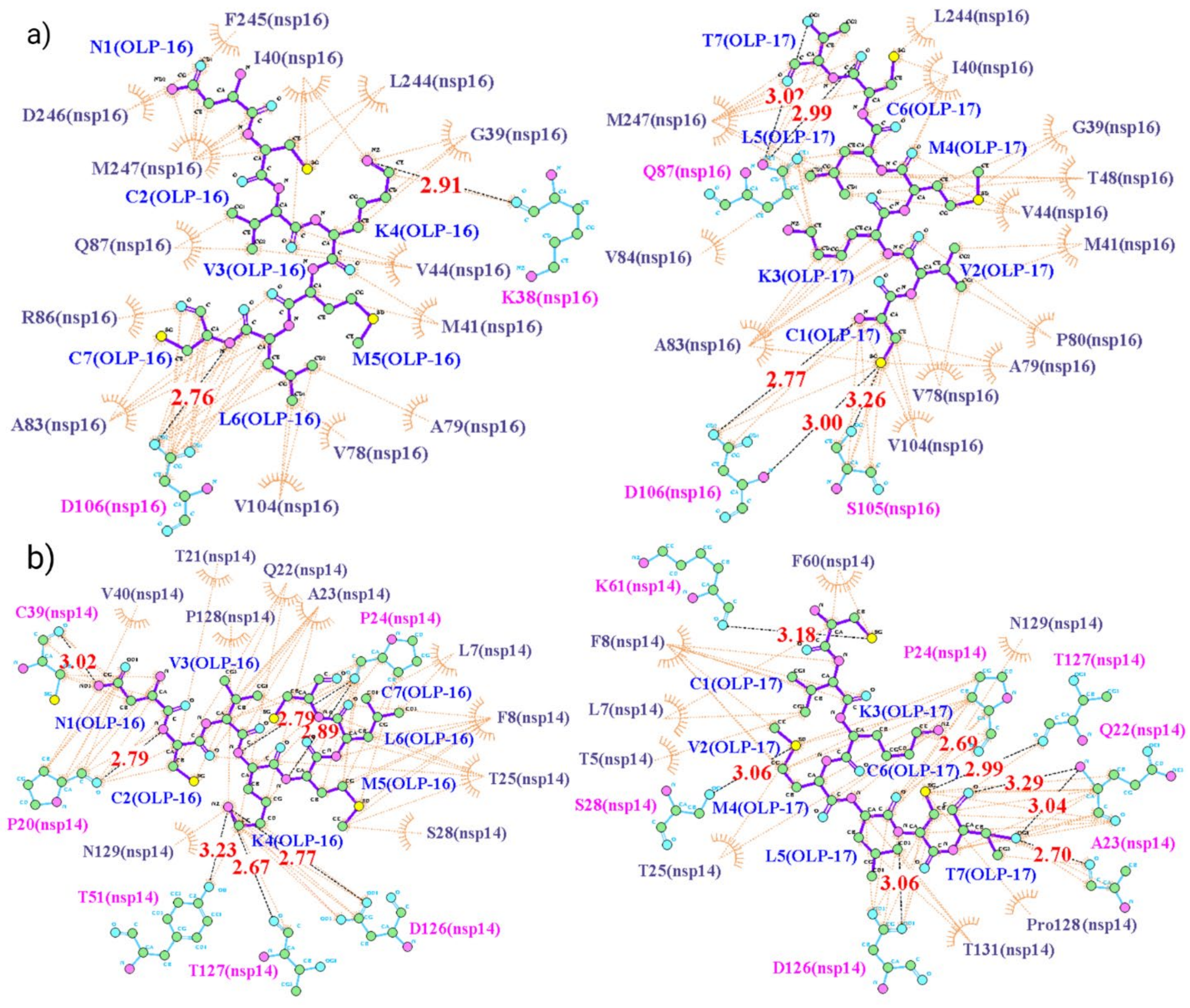

Figure S28: Two-dimensional residue interaction maps of OLP-16 and OLP-17 peptides and the targets. (a) OLP-16 and OLP-17 peptide-protein interactions when target was nsp16, and (b) OLP-16 and OLP-17 peptide-protein interactions when target was nsp14. The black dashed lines and brown arcs with spokes represent the hydrogen bonds and the hydrophobic contacts, respectively.

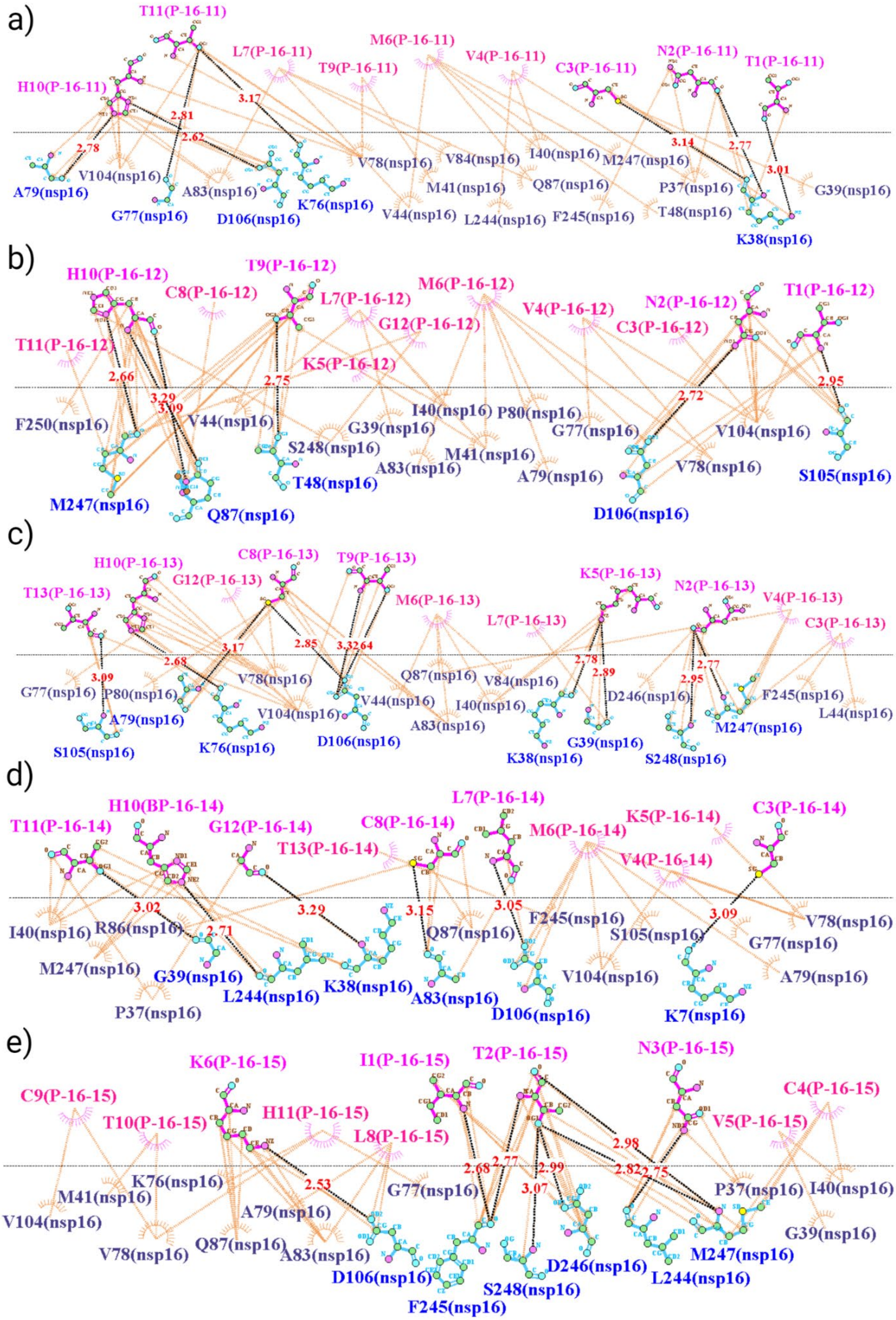

a)
T11(P-16-11)
L7(P-16-11)
M6(P-16-11)
T9(P-16-11)
V4(P-16-11)
C3(P-16-11)
N2(P-16-11)
T1(P-16-11)
H10(P-16-11)
3.17
2.81
2.78
2.62
3.14
2.77
3.01
A79(nsp16)
V104(nsp16)
G77(nsp16)
A83(nsp16)
D106(nsp16)
K76(nsp16)
V78(nsp16)
V84(nsp16)
M41(nsp16)
V44(nsp16)
L244(nsp16)
I40(nsp16)
Q87(nsp16)
F245(nsp16)
M247(nsp16)
P37(nsp16)
T48(nsp16)
G39(nsp16)
K38(nsp16)
b)
H10(P-16-12)
C8(P-16-12)
T9(P-16-12)
L7(P-16-12)
M6(P-16-12)
G12(P-16-12)
V4(P-16-12)
N2(P-16-12)
T1(P-16-12)
C3(P-16-12)
T11(P-16-12)
K5(P-16-12)
2.66
3.29
3.09
2.75
2.72
2.95
F250(nsp16)
V44(nsp16)
I40(nsp16)
P80(nsp16)
G39(nsp16)
S248(nsp16)
G77(nsp16)
V104(nsp16)
M41(nsp16)
A83(nsp16)
A79(nsp16)
V78(nsp16)
S105(nsp16)
T48(nsp16)
M247(nsp16)
Q87(nsp16)
D106(nsp16)
c)
H10(P-16-13)
C8(P-16-13)
T9(P-16-13)
G12(P-16-13)
T13(P-16-13)
M6(P-16-13)
K5(P-16-13)
N2(P-16-13)
V4(P-16-13)
L7(P-16-13)
C3(P-16-13)
3.17
2.85
3.32
2.64
3.09
2.68
2.78
2.89
2.95
2.77
G77(nsp16)
P80(nsp16)
V78(nsp16)
Q87(nsp16)
V84(nsp16)
D246(nsp16)
F245(nsp16)
L44(nsp16)
I40(nsp16)
V44(nsp16)
V104(nsp16)
A79(nsp16)
A83(nsp16)
S105(nsp16)
K76(nsp16)
D106(nsp16)
K38(nsp16)
G39(nsp16)
S248(nsp16)
M247(nsp16)
d)
H10(BP-16-14)
L7(P-16-14)
T11(P-16-14)
G12(P-16-14)
C8(P-16-14)
M6(P-16-14)
K5(P-16-14)
C3(P-16-14)
T13(P-16-14)
V4(P-16-14)
3.02
2.71
3.29
3.15
3.05
3.09
I40(nsp16)
R86(nsp16)
M247(nsp16)
G39(nsp16)
P37(nsp16)
L244(nsp16)
K38(nsp16)
A83(nsp16)
Q87(nsp16)
F245(nsp16)
S105(nsp16)
V104(nsp16)
D106(nsp16)
V78(nsp16)
G77(nsp16)
A79(nsp16)
K7(nsp16)
e)
K6(P-16-15)
I1(P-16-15)
T2(P-16-15)
N3(P-16-15)
C9(P-16-15)
T10(P-16-15)
H11(P-16-15)
L8(P-16-15)
C4(P-16-15)
V5(P-16-15)
2.98
2.53
2.68
2.77
2.99
3.07
2.82
2.75
K76(nsp16)
M41(nsp16)
V104(nsp16)
G77(nsp16)
A79(nsp16)
V78(nsp16)
Q87(nsp16)
A83(nsp16)
P37(nsp16)
I40(nsp16)
G39(nsp16)
D106(nsp16)
F245(nsp16)
S248(nsp16)
D246(nsp16)
L244(nsp16)
M247(nsp16)

Figure S29: Two-dimensional residue interaction maps for P-16-11, P-16-12, P-16-13, P-16-14, P-16-15 and nsp16 as target protein. (a) P-16-11-nsp16 complex, (b) P-16-12-nsp16 complex, (c) P-16-13-nsp16 complex, (d) P-16-14-nsp16 complex, and (e) P-16-15-nsp16 complex. The horizontal dashed line represents the interface. The black dashed lines and pink and brown arcs with spokes represent the hydrogen bonds and the hydrophobic contacts, respectively.

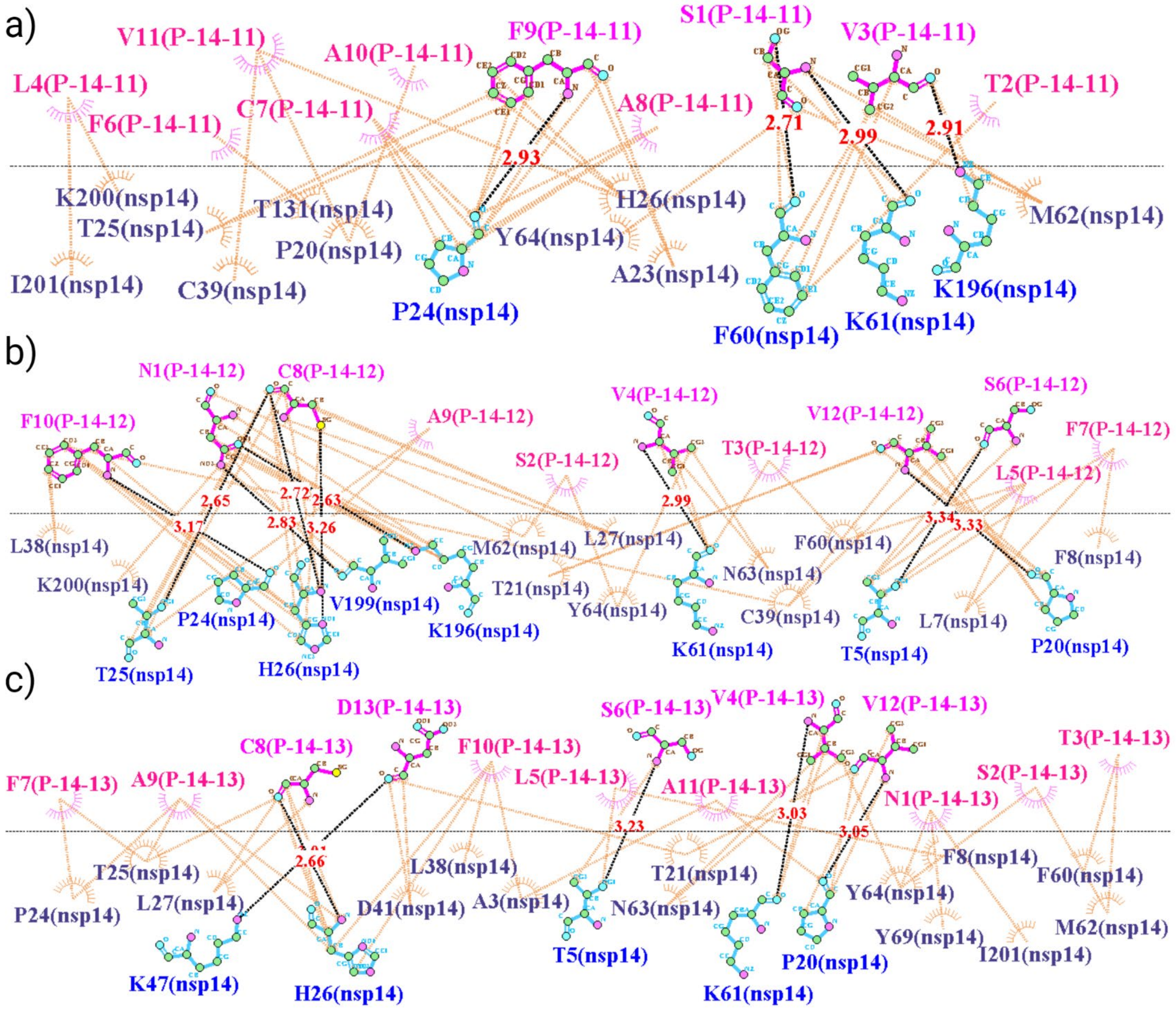

Figure S30: Two-dimensional residue interaction maps for P-14-11, P-14-12, P-14-13 and nsp14 as target protein. (a) P-14-11-nsp14 complex, (b) P-14-12-nsp14 complex, and (c) P-14-13-nsp14 complex. The horizontal dashed line represents the interface. The black dashed lines and pink and brown arcs with spokes represent the hydrogen bonds and the hydrophobic contacts, respectively.

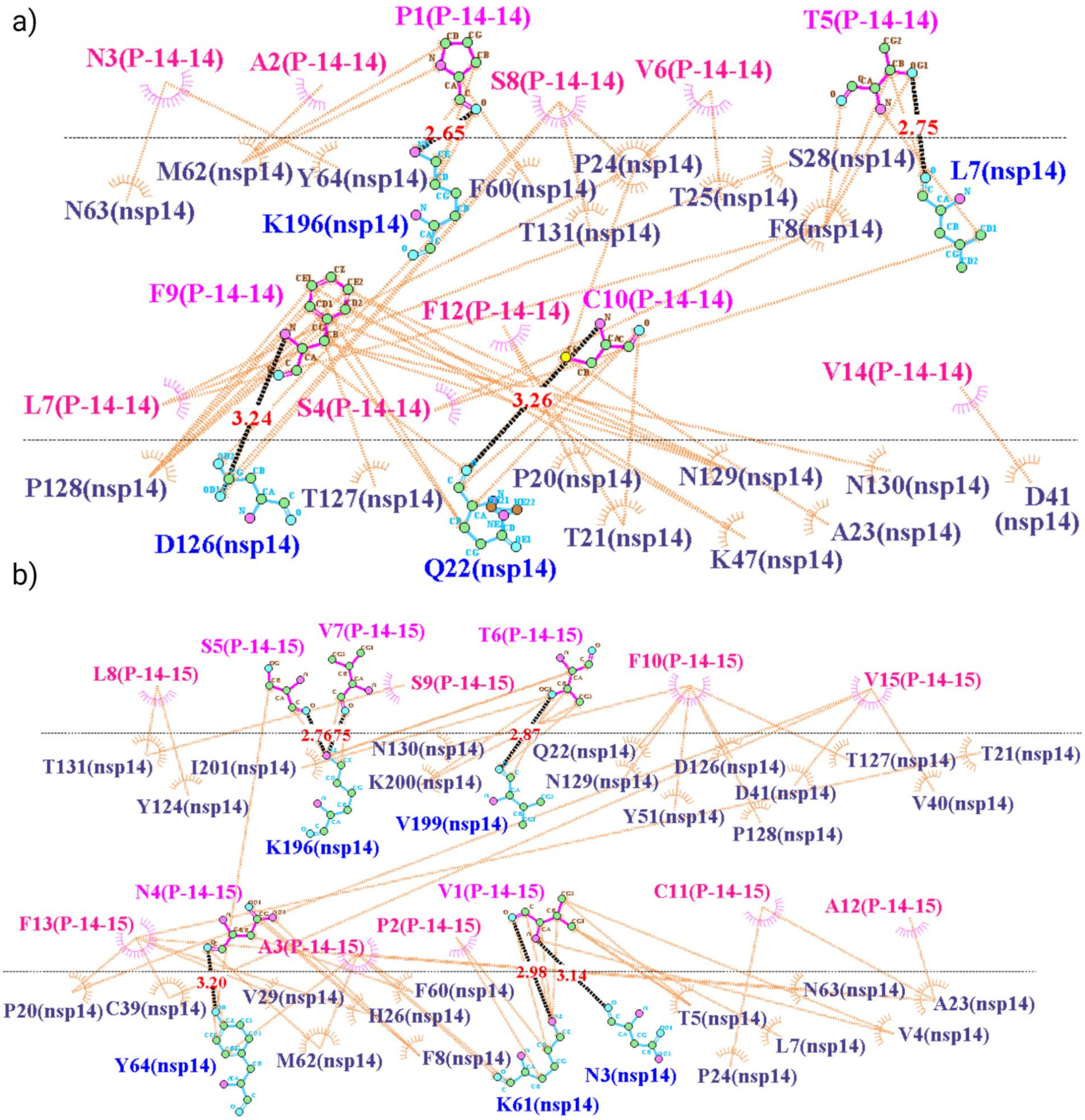

Figure S31: Two-dimensional residue interaction maps for P-14-14 and P-14-15 and nsp14 as target protein. (a) P-14-14-nsp14 complex, and (b) P-14-15-nsp14 complex. The horizontal dashed line represents the interface. The black dashed lines and pink and brown arcs with spokes represent the hydrogen bonds and the hydrophobic contacts, respectively.

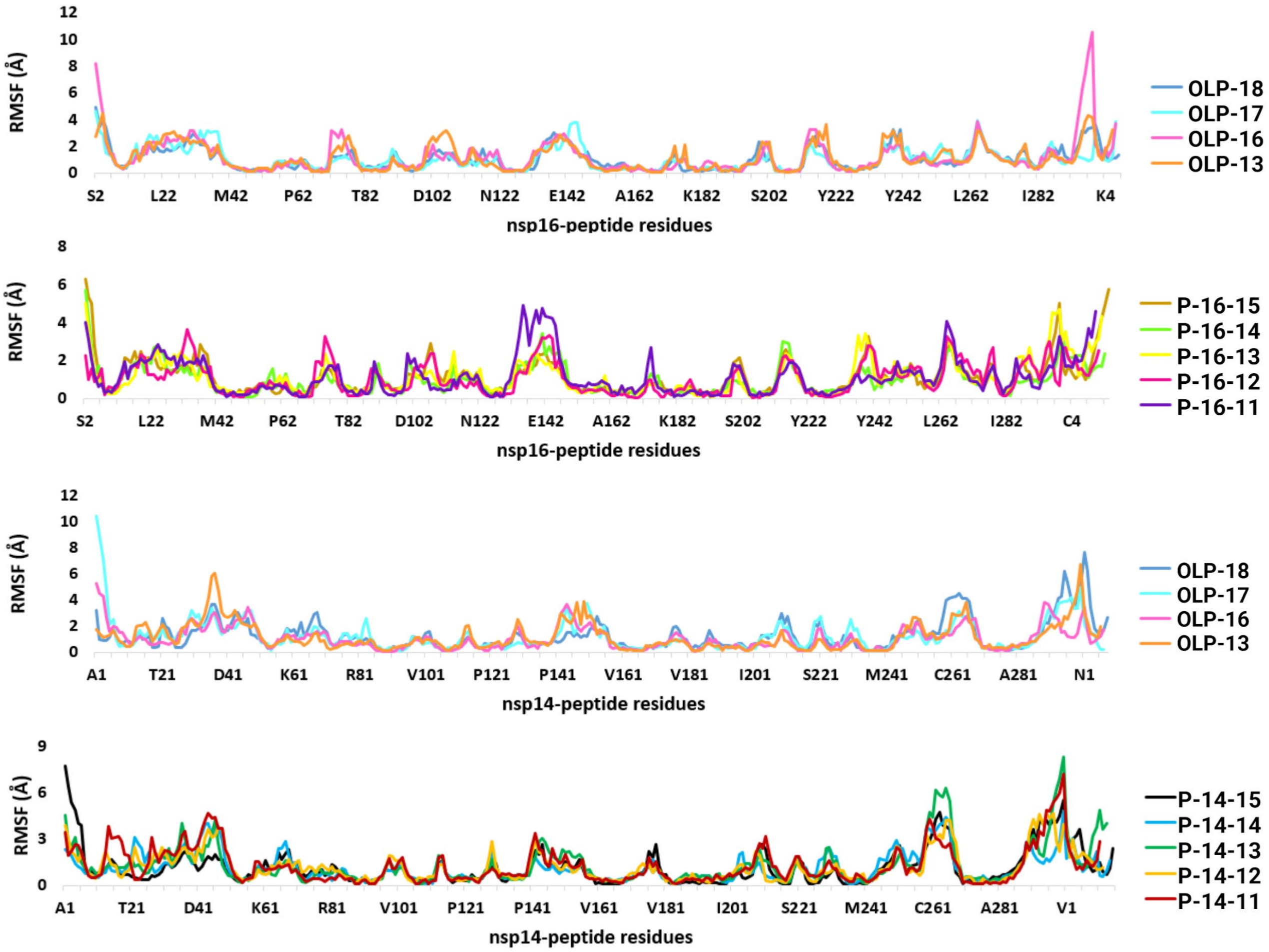

Figure S32: RMSF plots of the target-peptide complexes. The plots represent the fluctuations of the residues of the target protein (nsp16 and nsp14) and the designed peptides (OLP-13, OLP-16, OLP-17, OLP-18, P-16-11, P-16-12, P-16-13, P-16-14, P-16-15, P-14-11, P-14-12, P-14-13, P-14-14, and P-14-15) during the simulations.

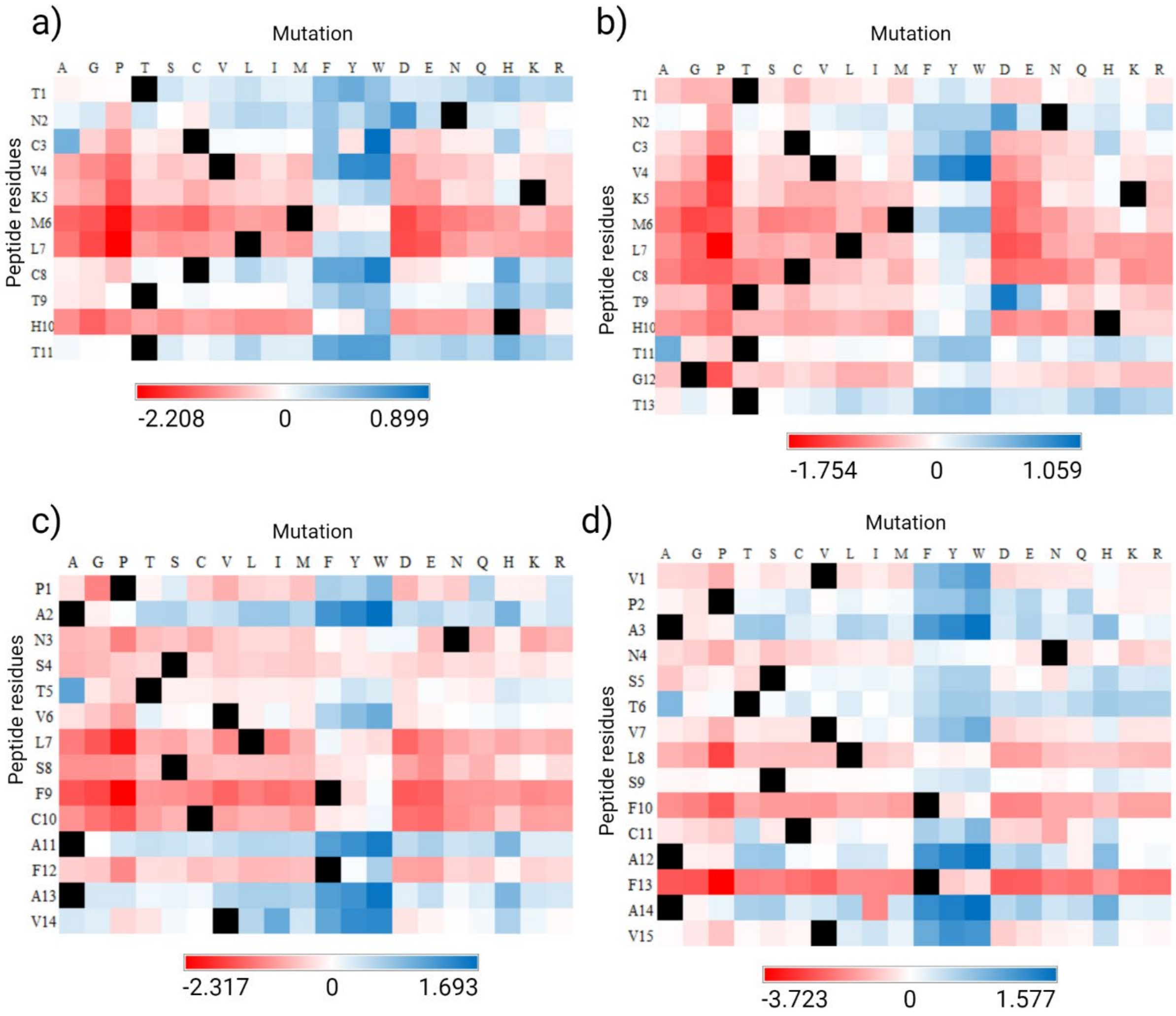

Figure S33: Heat maps representing *in silico* saturation mutagenesis of the designed peptides. (a) P-16-11 and nsp16 interaction, (b) P-16-13 and nsp16 interaction, (c) P-14-14 and nsp14 interaction, and (d) P-14-15 and nsp14 interaction. Mutations with the improving (positive $\Delta\Delta G_{Affinity}$) and decreasing (negative $\Delta\Delta G_{Affinity}$) impacts on peptide-target binding affinity are shown in blue and red, respectively.

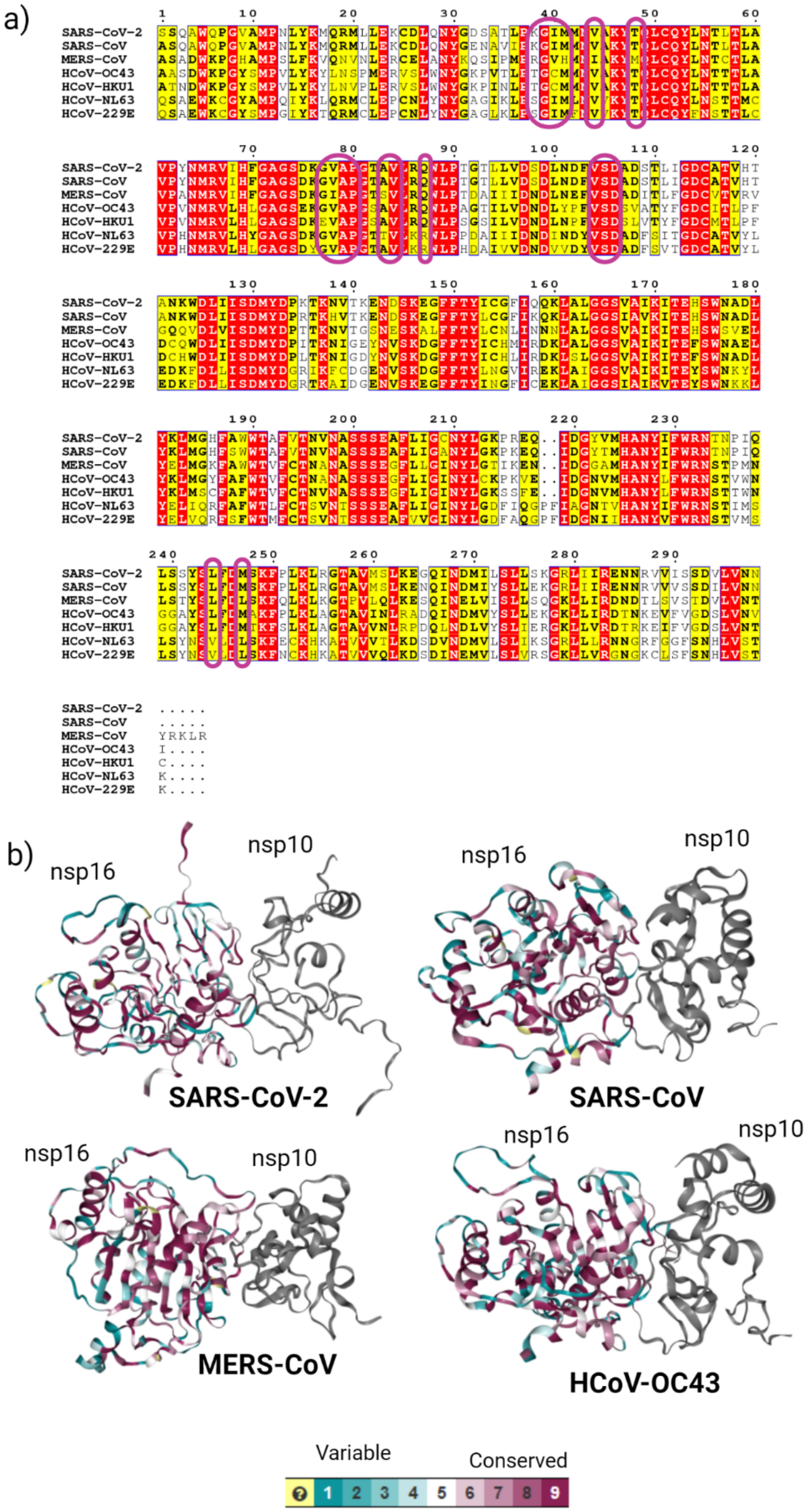
a)
SARS-CoV-2
SARS-CoV
MERS-CoV
HCoV-OC43
HCoV-HKU1
HCoV-NL63
HCoV-229E
b)
nsp16
nsp10
SARS-CoV-2
SARS-CoV
MERS-CoV
HCoV-OC43
Variable
Conserved
1 2 3 4 5 6 7 8 9

Figure S34: Conservation analysis of the nsp16 protein across coronaviruses. (a) Multiple sequence alignment of nsp16 sequences from seven human CoVs, including SARS-CoV-2 (UniProt ID P0DTD1), SARS-CoV (UniProt ID P0C6X7), MERS-CoV (UniProt ID K9N7C7), HCoV-OC43 (UniProt ID P0C6X6), HCoV-HKU1 (UniProt ID P0C6X2), HCoV-NL63 (UniProt IDP0C6X5), and HCoV-229E (UniProt ID P0C6X1). The purple boxes denote the residues of nsp16 targeted by nsp16 designed peptides. Identical residues are shown in red boxes with white characters. Similar residues are shown in bold black characters and boxed in yellow. (b) Analysis of nsp16 conservation across four CoVs: SARS-CoV-2, SARS-CoV, MERS-CoV, and HCoV-OC43. The structures were colored from the most conserved in dark magenta (with a conservation score of 9) to the most variable in turquoise (with a conservation score of 1).

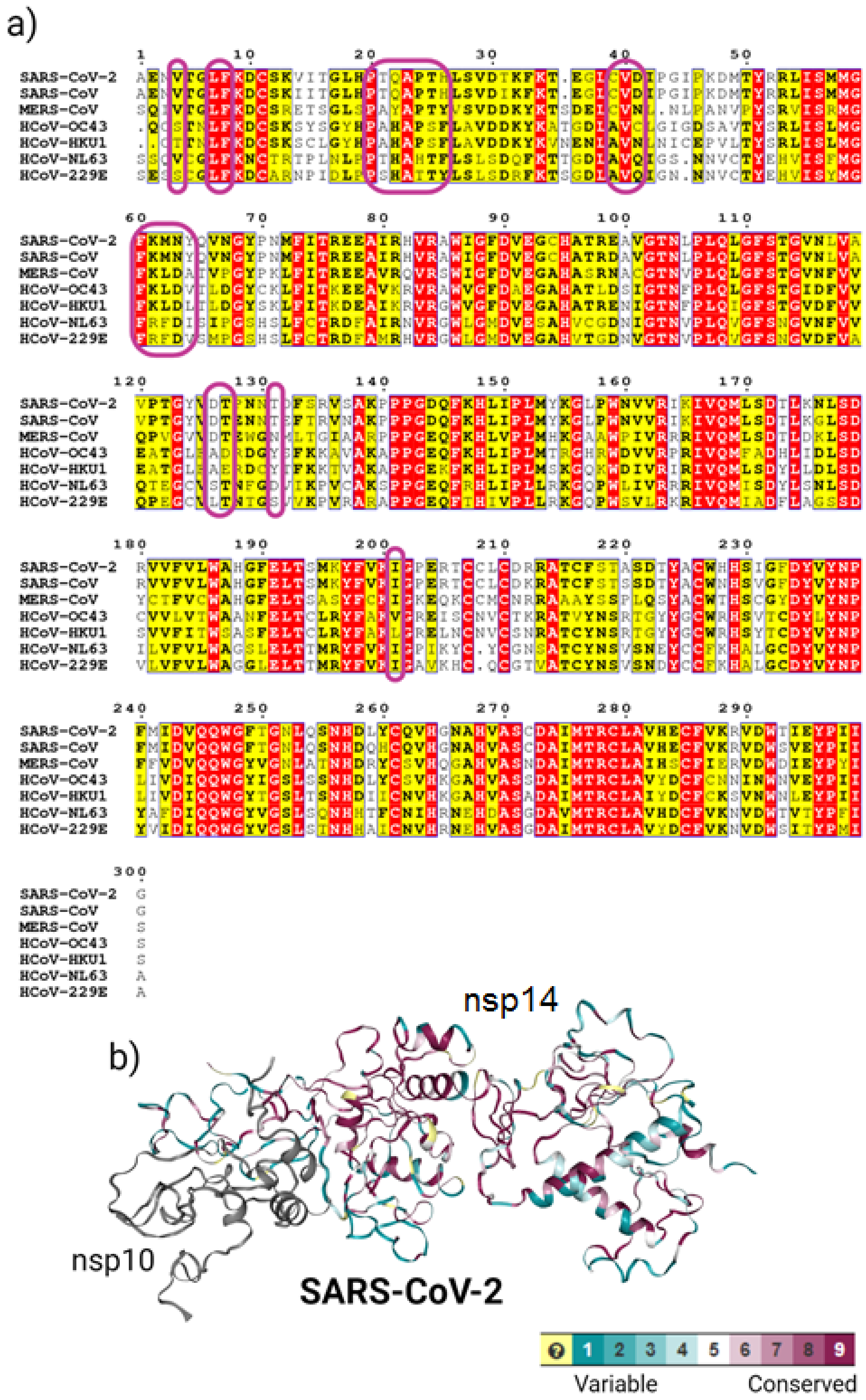

Figure S35: Conservation analysis of the nsp14 protein across coronaviruses. (a) Multiple sequence alignment of nsp14 sequences from seven human CoVs, including SARS-CoV-2 (UniProt ID P0DTD1),

SARS-CoV (UniProt ID P0C6X7), MERS-CoV (UniProt ID K9N7C7), HCoV-OC43 (UniProt ID P0C6X6), HCoV-HKU1 (UniProt ID P0C6X2), HCoV-NL63 (UniProt IDP0C6X5), and HCoV-229E (UniProt ID P0C6X1). The purple boxes denote the residues of nsp14 targeted by nsp14 designed peptides. Identical residues are shown in red boxes with white characters. Similar residues are shown in bold black characters and boxed in yellow. (b) Conservation analysis of SARS-CoV-2 nsp14 protein model. The structure was colored from the most conserved in dark magenta (with a conservation score of 9) to the most variable in turquoise (with a conservation score of 1).

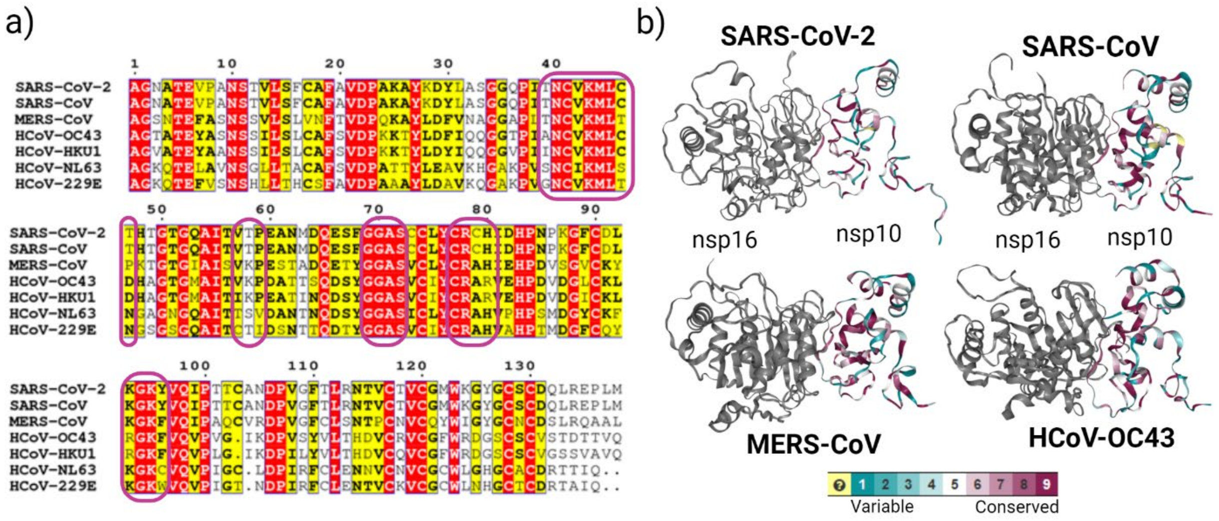

Figure S36: Conservation analysis of the nsp10 protein across coronaviruses. (a) Multiple sequence alignment of nsp10 sequences from seven human CoVs, including SARS-CoV-2 (UniProt ID P0DTD1), SARS-CoV (UniProt ID P0C6X7), MERS-CoV (UniProt ID K9N7C7), HCoV-OC43 (UniProt ID P0C6X6), HCoV-HKU1 (UniProt ID P0C6X2), HCoV-NL63 (UniProt IDP0C6X5), and HCoV-229E (UniProt ID P0C6X1). The purple boxes denote the overlapping key interacting residues of nsp10, which interact with both nsp16 and nsp14. Identical residues (N40, C41, K43, M44, L45, G69, G70, A71, S72, C77, R78, G94, and K95) are shown in red boxes with white characters. Similar residues (V42, C46, T47, V57, P59, C79, H80, K93, and Y96) are shown in bold black characters and boxed in yellow. (b) Analysis of nsp10 conservation across four CoVs: SARS-CoV-2, SARS-CoV, MERS-CoV, and HCoV-OC43. The structures were colored from the most conserved in dark magenta (with a conservation score of 9) to the most variable in turquoise (with a conservation score of 1). G69, G70, A71, S72, C77, and R78 are the most highly conserved interface residues with a conservation score of 9 (dark magenta). N40, C41, V42, K43, M44, L45, P59, C79, H80, K93, G94, K95, and Y96, are well conserved with conservation scores of 6-8 (pink). T39, C46, T47, V57, T58, are intermediately conserved or variable (from white to turquoise).
